# RiTex: Harmonization of Radiomic Features Based on Riemannian Geometry

**DOI:** 10.3390/jimaging12060264

**Published:** 2026-06-17

**Authors:** Darya A. Voitenko, Anton V. Vladzymyrskyy, Olga V. Omelyanskaya, Yuriy A. Vasilev, Ivan A. Blokhin, Maria R. Kodenko

**Affiliations:** 1 State Budget-Funded Health Care Institution of the City of Moscow “Research and Practical Clinical Center for Diagnostics and Telemedicine Technologies of the Moscow Health Care Department”, 127051 Moscow, Russia; voytenko.da20@physics.msu.ru (D.A.V.); 2 Federal State-Funded Educational Institution of Higher Education, Lomonosov Moscow State University, 119991 Moscow, Russia

**Keywords:** radiomics, batch effects, harmonization, Riemannian geometry, SPD matrices, Fréchet mean, generalized eigenvalue problem, ComBat, CovBat, multi-center validation

## Abstract

Batch effects arising from variations in hardware, acquisition protocols, and reconstruction parameters present a critical challenge in radiomics, limiting the generalizability of models across multicentre studies. Existing harmonization methods, such as ComBat, CovBat, z-score normalization, and Generative Adversarial Networks, exhibit significant limitations when applied to high-dimensional radiomic data. ComBat assumes a linear feature space and tends to leave residual center-specific information recoverable by downstream classifiers. This paper introduces RiTex (Riemannian Texture Harmonization), a framework that solves a generalized eigenvalue problem between class-aware biological scatter and Ledoit–Wolf-regularized per-batch covariances, with the SPD-manifold Fréchet mean used as a principled averaging step. We evaluate RiTex on the 50-dataset radMLBench benchmark and on a new four-center head-and-neck benchmark with known center labels ( n = 380 patients, k = 4 centers from TCIA: HGJ, MDACC, Maastro, QIN). On radMLBench, RiTex reduces the batch auto-detection AUC in 48/50 (96%) datasets, 42/50 (84%) reductions remain significant after Benjamini–Hochberg correction; the mean Batch AUC reduction is ΔBatch = −0.365 (95% bootstrap CI [−0.418, −0.312]), with no significant degradation in biological AUC (mean ΔBio = +0.018, 95% CI [−0.011, +0.047]). On the H&N benchmark with real center labels, RiTex reduces the Batch AUC from 0.74 to 0.59, while ComBat and CovBat leave it at ≈0.98. A component-wise ablation shows that the dominant source of empirical performance is the GEVD step, together with Ledoit–Wolf shrinkage. The SPD Fréchet mean acts as a theoretical scaffold with a negligible empirical contribution (ΔBatch AUC = −0.014 vs. arithmetic mean).

## 1. Introduction

Radiomics [1] involves deep analysis of medical imaging biomarkers (MIBs), utilizing mathematical modeling and machine learning to develop prognostic and diagnostic tools. The reproducibility of radiomic features remains low [2,3,4].

This lack of reproducibility stems from the high sensitivity of radiomic features to acquisition parameters, including scanner manufacturer, tube voltage, and reconstruction algorithms. These factors induce systematic variations, resulting in distinct sample groupings known as batches. Batch effects can create spurious correlations between the features.

Modern computational biology and high-dimensional data analysis offer multiple approaches to dataset harmonization, ranging from classical statistical transformations to advanced deep learning architectures. One of the simplest and earliest approaches is feature standardization, also known as z-score harmonization [5].

Despite its computational simplicity, z-score normalization has a significant drawback: it equalizes means and variances across all batches, potentially obliterating genuine biological differences in cohorts with unbalanced batch distributions.

ComBat, a more flexible and statistically rigorous method, is based on an Empirical Bayes framework [6]. Its core mechanics involve “borrowing information” across genes (subsequently adapted for radiomic features) within a batch to refine the estimation of additive $\gamma_{ij}$ and multiplicative $\delta_{ig}$ batch effects. By utilizing prior distributions, the method achieves robustness even with small sample sizes, preventing overcorrection and preserving the data structure more effectively than nonparametric approaches.

To model complex nonlinear relationships and heterogeneous effects, Bayesian Additive Regression Trees (BART [7]) are employed. BART allows for the flexible approximation of batch effect structures without rigid parametric assumptions, making it an effective tool for causal processing and data correction tasks in contexts with complex feature interactions. However, its high computational requirements limit its scalability to very large datasets.

With advancements in deep learning, variational autoencoders (VAEs) have gained attention for their ability to construct latent data representations that are invariant to technical noise. [8]. The VAE architecture minimizes the Evidence Lower Bound (ELBO) [9], a loss function comprising the reconstruction error and the Kullback–Leibler divergence.

Models such as scVI [10] feed the batch variable to the decoder, forcing the encoder to learn a latent z space that is invariant to the batch effect. This separates the biological signal from technical noise, ensuring high-fidelity integration of data across modalities.

An alternative approach to neural network correction is generative adversarial networks (GANs [11]), which address the harmonization problem through domain adaptation. The classic architecture includes a generator G and a discriminator D, engaged in a minimax competition.

To mitigate batch effects, the discriminator is trained to identify the batch origin of a sample, while the generator transforms the data to prevent the discriminator from determining its origin. These approaches (such as BatchGAN [12]) enable effective alignment of data distributions, even when inter-batch variations are extreme, producing data that are visually and statistically indistinguishable. However, the current challenge related to the mismatch between the number of extracted features and the number of patients in the sample makes GAN-based methods vulnerable to small samples.

The proliferation of diverse harmonization techniques underscores the problem of batch effects in radiomics. Each of these approaches possesses distinct advantages and limitations, primarily dictated by the non-uniform structure of the input data. The assumption that texture features reside in a Euclidean space precludes an accurate estimation of their true proximity on a plane. Furthermore, in a high-dimensional feature space (approximately 1000 features per region of interest (ROI)), the very concept of distance becomes irrelevant, as points in such a space tend to be equally spaced, which contradicts the actual data distribution. The sensitivity of Euclidean space to scale presents an additional barrier when processing features with disparate physical interpretations and dynamic ranges. Therefore, to account for multicollinearity, nonlinearity, and the intrinsic topological nature of feature dependencies, we propose a shift toward an alternative geometric framework for harmonization.

The goal of this study was to develop and validate RiTex, a method for correcting batch effects in radiomics that combines (i) a class-aware biological scatter matrix built from Fisher LDA with an interpretable hybrid biological–correlation weighting, (ii) Ledoit–Wolf regularized per-batch covariances, and (iii) a generalized eigenvalue problem whose solution defines a single linear projector. We use the SPD manifold and the Fréchet mean as the mathematical scaffold that justifies the well-posedness of the averaging step and report a component-wise ablation that distinguishes which parts of the construction translate into empirical performance and which serve as theoretical scaffolding. We validate the method on the 50-dataset radMLBench benchmark and, separately, on a four-center head-and-neck benchmark with real center labels, which allows us to characterize the regime in which the method does and does not outperform ComBat and CovBat.

The scope of this study is restricted to the comparison of classical mathematical harmonization methods, that is, methods whose batch-effect model is fully specified by a closed-form statistical or geometric construction and is fitted in a single deterministic pass over the data. Within this class, we compare RiTex against the two most widely adopted radiomic-harmonization baselines: ComBat, a location–scale empirical Bayes estimator on the feature marginals, and CovBat, which extends ComBat by an additional correction of the principal components of the residual covariance. Deep-learning harmonization methods—including domain-adversarial neural networks (DANN) [13], variational auto-encoders such as scVI, originally proposed for single-cell RNA-seq, and MMD-regularized residual networks [14]—are excluded from the benchmark by design. These methods belong to a different methodological class (stochastic non-convex optimization of an over-parameterized neural model rather than a closed-form statistical or geometric estimator) and are not directly comparable to ComBat, CovBat, or RiTex on the criteria adopted here (closed-form fit, deterministic output, no architectural hyperparameter search, training time below one second per fold on a single CPU). A dedicated comparison between RiTex and deep-learning baselines is the subject of follow-up work.

## 2. Materials and Methods

The study was reviewed and approved by the Independent Ethics Committee (Protocol № 2/2026, dated 19 February 2026) and conducted in accordance with the principles of Good Clinical Practice (ICH GCP) and the legislation of the Russian Federation.

Radiomic features can be conceptualized as a point cloud in a multidimensional space, where each point corresponds to an image or a ROI. The batch effect occurs when the points within a single batch form a subset that is shifted and distorted relative to others [15]. This phenomenon manifests as an affine transformation and a nonlinear distortion.

Using Riemannian geometry, the data are transformed across manifolds of positive-definite matrices. Batch effects often manifest as shifts along geodesics, which characterize specific distortions. This transformation must ensure reversibility, preserve the intrinsic batch geometry, and establish comparable distances between samples from different batches [16].

Before global transformation, a detailed analysis of the batch effect by feature is necessary. It can manifest itself through location effects and scale effects. Location effects are estimated via inter-batch variance, while scale effects within covariance matrices are estimated in Riemannian space.

Classifying features by sensitivity to the batch effect enables the implementation of a differentiated correction strategy. The transformation necessitates quality control to ensure that biological variability is preserved. Data visualization techniques, such as tSNE or UMAP [17], should demonstrate a better blending of points between batches and the preservation of the cluster structure.

Consequently, this geometric approach to feature harmonization serves as the foundation of the RiTex algorithm.

### 2.1. Mathematical Basis

In this section, we formulate the problem of radiomic data harmonization as the task of identifying an optimal linear mapping on a Riemannian manifold.

Let $X=\;{\left\{\left(x_{i},\;y_{i},b_{i}\right)\right\}}_{i=1}^{N}$ be a dataset, where $x_{i}\in R^{p}$ represents a vector of p radiomics features, y is the diagnostic status, and b is the batch identifier (i.e., scanner, acquisition protocol, or institution).

We model the observed feature vector $x_{ij}$ (the *i*-th sample in the *j*-th batch) as a random variable whose distribution depends on both the biological state and the technical characteristics of the batch. The goal is to find a mapping $\Phi :R^{p}\to R^{r}\left(r\leq p\right)$ , such that the mutual information between the data and the batch label *I*(*Z*,*B*) is minimized in the latent space Z=Φ(x) , while maximizing the mutual information with the target variable *I*(*Z*,*Y*).

RiTex is applied against restrictions to the class of linear mappings $z=\;W^{T}x$ , where $W\in R^{p\times r}$ is the orthogonal projection matrix.

Although the feature covariance matrix is the central component, the traditional Euclidean metric in this space has drawbacks [18], such as the “swelling” effect and a lack of invariance.

To address these issues, we apply the Fisher–Rao metric [19], which is invariant to congruent transformations. This is critical in radiomics to ensure that harmonization outcomes are independent of linear scaling or feature rotation.

The Fréchet mean [20] is utilized to identify the central tendency across groups of combined features. Despite its computational complexity, this method identifies a single representative point around which the feature harmonization is executed.

### 2.2. Practical Role of the SPD Framework

The Riemannian SPD construction provides a mathematically principled framework for (i) averaging per-batch covariance matrices via the Fréchet mean and (ii) measuring distances between the covariance matrices via the affine-invariant metric [21]. Conceptually, this avoids the swelling effect inherent in Euclidean averaging of covariance matrices and yields a unique minimizer on the Hadamard manifold of SPD matrices. As we show empirically in Section 3.2, the empirical contribution of this geometric structure in practice is small.

On our benchmarks, replacing the Fréchet mean with the arithmetic mean of the per-batch covariances changes the corrected Batch AUC by less than 0.015. We therefore present the SPD machinery as a theoretical scaffold that justifies the well-posedness and the uniqueness of the averaging step, rather than as the empirical source of harmonization performance. The dominant empirical contribution comes from the GEVD step (Section 2.4, Equation (1)) combined with Ledoit–Wolf regularization of the per-batch covariances (Section 2.5).

### 2.3. Feature Selection

Given the high dimensionality of radiomic data ( p≫n ), filtering based on the ANOVA F-statistic [22] is used to select the k most informative features. This stabilizes the subsequent covariance estimation.

For each feature j, the F statistic is calculated, measuring the ratio of inter-class to intra-class variance.

Subsequently, a check is performed to ensure that the number of selected features satisfies the dimensionality criteria and to construct a final feature matrix of dimension N×k .

### 2.4. Solving the Generalized Eigenvalue Problem (GEP)

Solving the GEP [23] is a key computational step in the RiTex framework, determining the optimal approaches to radiomic data harmonization. The classical eigenvalue problem, widely used in principal component analysis (PCA) and other linear algebra methods, seeks vectors and scalars satisfying the relation *Aw* = *λw*. However, multicenter radiomics data harmonization is a more complex challenge requiring simultaneous optimization of two criteria: maximizing the biological signal and minimizing the batch effects, as reflected in Formula (1). The solution to the GEP provides the required optimization for this task.

(1) $${\;\;\;\;\;\;\;\;\;\;\;\;\;\;\;\;\;\;S}_{bio,combined}w=\lambda S_{batch}w\;\;\;\;\;\;\;\;\;\;\;\;\;\;\;\;\;\;\;\;\;\;\;\;$$

where *λ* represents the generalized eigenvalues, and w represents the corresponding generalized eigenvectors. Solving this problem enables the calculation of geodesics in the feature space that maximize the ratio of biological variability to the batch effects, forming the mathematical foundation for harmonization.

The GEP formulation avoids the need for arbitrary regularization parameters, which would otherwise be necessary in the classical formulation. Instead, the GEP naturally integrates biological and batch variance into a unified task. In this context, the question resolves into a classification problem relative to diagnostic status labels, which, in this study, are binary (benign and malignant). However, it can also be generalized to multi-class labels, which can be used further as the method evolves.

Numerical solutions to the GEP provide a stable and efficient means of obtaining the necessary eigenvalues and eigenvectors. Eigenvalues offer a direct interpretation as signal-to-noise ratios for each geodesic, facilitating a quality assessment of the resulting harmonization curves. Eigenvectors define the geodesics in the feature space along which these ratios are optimized and are used to transform the original data into a new representation with enhanced statistical performance.

### 2.5. Construction of Biological Variance Matrices

In the classical GEP formulation, the biological variance matrix is defined as the between-group scatter matrix.

Geometrically, the matrix $S_{bio\;}$ encodes information about class differences within feature space. In binary classification, the difference vector between the centroids indicates the direction in which the differences between classes are most clearly visible. The matrix $S_{bio\;}$ is a rank-one matrix, represented by the outer product of the difference vector with itself (with a coefficient that depends on the group sizes).

We also define the within-group scatter matrix $S_{W}$ , which encodes the data variability within each class. There is a fundamental relationship between these matrices, called the total variance decomposition. This relationship reflects the fundamental ANOVA principle: the total data variation is decomposable into components explained by inter-group differences and intra-group variation.

**Figure 1:**
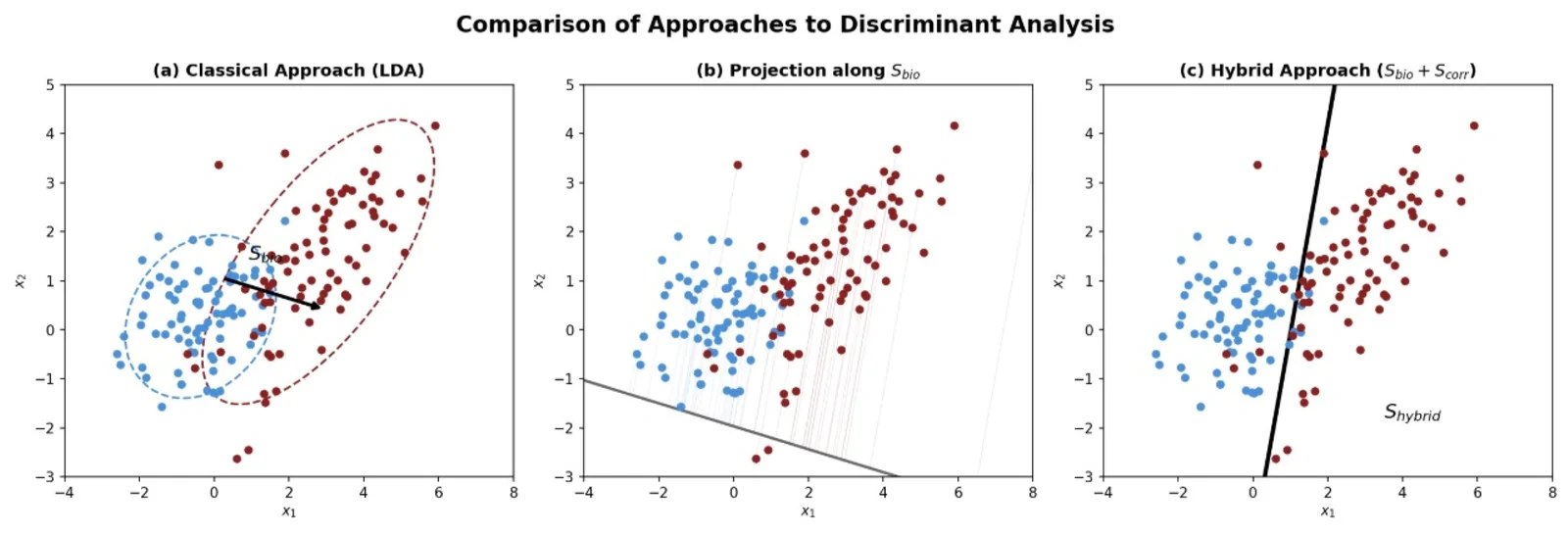
Approaches to discriminant analysis. (**a**)—The classical approach that utilizes centroids and ignores different covariance structures of distributions. (**b**)—Intra-class correlations imply the intersection of class labels. (**c**)—A hybrid approach ensuring distinct class differentiation.

Although the classical matrix $S_{bio\;}$ , which is based solely on class discrimination and assumes the equality of covariance matrices, provides useful information, but it fails to fully capture the intra-class data structure. Particularly, when two classes exhibit significantly different covariance structures (which is often the case in medical data, where a disease might affect the features’ means and cross-correlations), relying on centroid differences alone may emphasize trends that achieve class differentiation at the cost of high intra-class variability (Figure 1).

To overcome this limitation, we introduce a hybrid approach that combines the matrix $S_{bio\;}$ with the features correlation structure. The goal is to enhance information about the discriminants with intra-class feature stability. Features that both distinguish between classes and exhibit stable (low) correlation structures serve as more robust indicators of the underlying biological state.

The feature correlation matrix (R) is calculated in the standard manner once the features are normalized to zero mean and unit variance.

In the context of batch effect harmonization, the correlation matrix captures essential information regarding feature interactions. Although batch effects often exert a systematic influence on correlations in a dataset, features carrying genuine biological information typically exhibit more resilient correlation patterns. For example, texture features representing related tumor characteristics naturally correlate with one another, and this correlation should remain relatively invariant to batch effects.

RiTex computes the combined biological variance matrix as a weighted combination (Equation (2)):

(2) $${\;S}_{bio,combined}=\left(1-\lambda \right)S_{bio}+\lambda S_{corr}$$

where λ is a hyperparameter controlling the balance between the two components. Here, $S_{corr}\;$ represents the correlation component, defined as the correlation matrix R.

The parameter λ takes values within the interval [0, 1]. When λ = 0, we use the classical matrix $S_{bio}$ , which is based solely on class discrimination. When λ = 1, we switch entirely to correlation information. In practice, the recommended value is in the range λ∈[0.1, 0.3] . This range was obtained through multiple experimentations to determine suboptimal algorithm parameters. This ensures a balance. The class discrimination information remains a large contributor, but a small additional weight is given to the robust correlation structure.

The combined matrix inherits positive semi-definiteness from both components, since a convex combination of positive semi-definite matrices is itself positive semi-definite. This property is critical for the generalized eigenvalue problem.

### 2.6. Constructing a Batch Variance Matrix with Ledoit–Wolf

Constructing this matrix requires a validated estimator for the covariance structure within each batch. However, in the high-dimensional feature spaces typical of radiomics, the standard sample covariance matrix becomes ill-conditioned and numerically unstable. The Ledoit–Wolf shrinkage estimator [24] provides a theoretically sound approach to regularizing the covariance matrix by shrinking it toward a well-conditioned target. In this framework, feature correlations were estimated using the standard method previously described.

RiTex employs a diagonal target for radiomic data harmonization for the following reasons.

First, this target preserves feature-specific variances attributable to the physical properties of the images (i.e., the variance of pixel intensity depends on Hounsfield unit ranges) [25], preserving the diagonal structure prevents information loss.

Second, the assumption of no correlations between features (only a diagonal target) is conservative and feasible when correlations are due to noise or technical artifacts rather than the underlying biological structure.

Third, the diagonal target has a low rank, making it more robust for radiomic data.

Fourth, employing a diagonal target yields a more stable solution to the GEP, as the harmonization directions are primarily determined by variance rather than correlations.

In practice, the tuning parameter α calculated by the Ledoit–Wolf method typically ranges from 0.1 to 0.5 for standard radiomic datasets. This implies that 50–90% of the information is derived from the sample covariance matrix, while 10–50% is sourced from the structured target matrix. This weighting typically achieves an optimal balance between preserving the data structure and ensuring numerical stability.

If α is close to 0 (<0.05), the sample covariance matrix is well-conditioned and requires minimal regularization. This occurs when the number of samples is relatively large relative to the dimensionality or the data exhibit a simple covariance structure.

Conversely, if α is close to 1, the sample covariance matrix is heavily distorted by noise and requires significant regularization. This typically results from high dimensionality combined with a limited batch of samples.

### 2.7. Experiment

The algorithm was evaluated using radMLBench (Radiomics Machine Learning Benchmark) [26]. The benchmark contains 50 datasets, with the metadata indicating the patient’s clinical status. The benchmark inclusion criteria were (a) radiomic features extracted from tomographic images (CT, MRI, or PET), (b) a sample size exceeding 50, (c) no more than two samples per feature, and (d) a minimum of ten positive cases. Studies involving non-human subjects, such as animals or phantoms, were excluded. Since the benchmark contains quantitative radiomic features, clinical data were removed, and the results were binarized across the datasets.

Computations were performed using the Google Colab virtual environment on a system equipped with an AMD Ryzen 5 5500 U processor (2.10 GHz). The average computation time per dataset was below five minutes, with RAM load being approximately 1.3 GB.

#### 2.7.1. Voxel Unification Protocol

All images across the datasets were resampled to a “1 mm × 1 mm × 1 mm” isotropic resolution using linear interpolation. This step is critical as the original spatial resolutions varied within each dataset, and texture features are sensitive to voxel dimensions. Standardizing the resolution eliminates one potential source of bias other than the batch effect.

#### 2.7.2. Intensity Normalization

Intensity normalization was performed using z-correction relative to the intensity in healthy tissue. Specifically, the mean intensity μ and standard deviation σ of the healthy tissue regions were calculated for each image, and each voxel was transformed using a common formula. This approach normalizes global intensity variations between scanners and acquisition protocols while preserving local contrast ratios, which are critical for feature extraction.

#### 2.7.3. Feature Extraction

Radiomic features were extracted from each segmented ROI according to the International Radiomics Initiative (IBSI) [27] recommendations.

More than 1000 features were extracted per ROI. In multimodal datasets, features were extracted independently for each imaging modality.

#### 2.7.4. Multi-Center Benchmark with Known Batch Labels

The radMLBench benchmark, although broad, does not preserve true center identifiers—batch affiliation must be inferred from data. To evaluate RiTex in the operationally important regime where the center of each scan is known, we constructed a second benchmark by merging four publicly available TCIA collections that share the same radiomic endpoint (“lymph-node metastasis presence”). See Table 1:

After normalizing the PyRadiomics feature names (stripping the modality and mask prefixes specific to each collection), 105 PyRadiomics features are common to all four datasets and form the harmonization matrix. Preprocessing (median imputation, variance threshold 10^−8^, RobustScaler) is fit strictly on the training fold and applied to the validation fold; no information leakage across folds is permitted.

#### 2.7.5. Preprocessing and Data Separation in the Absence of the Lack of Explicit Batch Affiliation

In some cases where explicit batch affiliation was missing from the metadata, we used hierarchical clustering based on global image statistics to identify hidden batch structures. Specifically, a global feature matrix was constructed for each image using the mean intensity, standard deviation, skewness, and kurtosis of the background (extra-ROI) regions. Hierarchical clustering with a Euclidean distance metric was applied to this matrix to identify natural clusters [28]. The resulting clusters were interpreted as batches, allowing for batch-specific validation of the harmonization methods even in the absence of explicit information about the data origin.

#### 2.7.6. Data Splitting for Internal Hyperparameter Search

When selecting hyperparameters for the RiTex method, the training set of each fold was partitioned into subsets for training (80%) and testing (20%). The search for optimal hyperparameters was performed on the testing subset to avoid overfitting and to ensure an unbiased assessment of the method’s generalization ability on the test subset.

#### 2.7.7. RiTex, ComBat, and CovBat: Architecture and Hyperparameter Tuning

To assess the harmonization performance of the RiTex algorithm, the ComBat algorithm with an empirical Bayes method for calculating both additive and multiplicative batch components was used as a reference test [29]. The “mean-only” mode was not used, meaning that the shift and scale of features within each batch were adjusted simultaneously. The empirical Bayes approach facilitated stable parameter estimation in small batches. A parametric version of ComBat was employed for a priori variance modeling, with hyperparameters of the prior variance distribution estimated across all available batches. In addition to ComBat, we include CovBat (Chen et al., 2022) [30] as a third-generation baseline. CovBat extends ComBat by additionally aligning the principal components of the covariance structure across batches, which makes it a stricter comparison for any method that claims to act on the covariance level. We use the implementation provided by the authors with the same intercept-only design matrix as ComBat.

The first unique batch identifier was designated as the reference; no clinical or demographic covariates were included in the design matrix (intercept-only), ensuring the harmonization remained agnostic to external variables. Implementation necessitated a minimum of two batches; partitions failing this requirement were excluded from the ComBat analysis.

The RiTex model includes the following core components:

1. Construction of SPD matrices from texture features: For each ROI, the texture features were structured as block matrices, with each block corresponding to a specific feature class. These matrices were transformed into SPD space by adding a regularization term (a diagonal matrix with a regularization parameter λ );
2. Evaluation of batch-specific transformations: Using the training set, a transformation matrix $T_{g}$ was estimated within the SPD space for each batch g . Specifically, the mean geodesic pole (Riemannian barycenter) [31] was calculated for the SPD matrices in each batch; the transformation matrix was defined as a mapping from the global centroid to the batch-specific centroid;
3. Geodesic correction: For each sample x in a batch g , the harmonized representation $x_{\mathrm{harmonized}}$ was calculated by applying the inverse Riemannian transformation defined in Equation (3):
(3) $${\;x}_{\mathrm{harmonized}}=T_{g}^{-1}\circ x,\;$$
  where ∘ denotes the Riemannian transformation.

#### 2.7.8. RiTex Hyperparameters

Hyperparameter selection for RiTex was performed outside the outer cross-validation loop, on a held-out stratified 20% subset of the training fold of the first cross-validation iteration. This avoids the bias that arises when the same data are used for both hyperparameter optimization and unbiased performance estimation (a bias that was present in the original submission’s per-fold search). We use random search with 20 samples drawn uniformly from the joint hyperparameter grid ( $n_{components}$ ∈ {5, 10, 20, 30}, $k_{features}$ ∈ {30, 50, 80}, λ ∈ {0.1, 0.2, 0.3, 0.5}, $\alpha_{LW}$ ∈ {0.1, 0.2, 0.3, 0.5}) and select the combination minimizing the weighted criterion J in Equation (4). Random search with this budget is known to match or exceed the performance of a full grid search (81 points) on continuous low-dimensional spaces and is ≈4× faster.

(4) $$J\left(\lambda ,\sigma ,\kappa \right)=\alpha \times \mathrm{Batch}\;\mathrm{AUC}+\beta \times \left(1-{\mathrm{Bio}\mathrm{AUC}}_{\mathrm{retention}}\right)$$

where ${\mathrm{Bio}\;\mathrm{AUC}}_{\mathrm{retention}}=\frac{{\mathrm{Bio}\;\mathrm{AUC}}_{\mathrm{harmonized}}}{{\mathrm{Bio}\;\mathrm{AUC}}_{\mathrm{original}}}$ determines the proportion of preserved biological information, and α and β —are weights (in the experiment, α=0.6 , β=0.4 ). The optimal hyperparameters were selected to minimize this function.

To secure stability, the optimal hyperparameters were re-evaluated at each of the ten cross-validation iterations on the testing subset. Hyperparameters providing the best mean criterion value J across all ten iterations were used.

Following selection, the RiTex model was retrained on the complete training partition using these optimized parameters, with the final performance evaluated on an independent test set for each cross-validation fold.

### 2.8. Statistical Analysis

#### 2.8.1. Evaluation Metrics: Bio AUC and Batch AUC

Bio AUC (Biological Area Under the Curve) measures the ability of harmonized features to preserve associations with clinically relevant variables. Specifically, for each feature, a univariate logistic regression model was constructed to predict the clinical outcome. The AUC value was calculated based on the ROC curve constructed on the test set.

After harmonization, the Bio AUC either remains stable or improves, indicating that the harmonization did not contribute to loss of biological information.

Batch AUC quantifies the residual batch effect remaining within the harmonized features. To assess the batch effect, a synthetic classification problem was created from the dataset, with the target variable representing the sample’s membership in a specific batch. Univariate logistic regression models were employed to predict the batch from each feature, with an AUC value calculated based on the ROC curve.

Optimal harmonization should minimize the batch AUC, approaching a theoretical value of 0.5 (which corresponds to random prediction). After harmonization, the batch AUC is expected to decrease significantly compared to the original value.

#### 2.8.2. Ten-Fold Stratified Cross-Validation

To evaluate harmonization performance, we used ten-fold stratified cross-validation, accounting for the batch distribution across the dataset. The partitioning into ten folds was based on [32] and the radiomic data specifics. This partitioning strategy ensured sufficient sample representation across folds, providing a robust framework for evaluating ComBat and RiTex. The procedure was as follows:

The data was partitioned into ten mutually exclusive folds, maintaining a consistent batch proportion within each fold.

In each iteration, one fold was used as the test set, and the remaining nine folds were combined into the training set. The hyperparameters of the harmonization methods were evaluated exclusively on the training set.

The batch effect parameters were estimated on the training set for each batch separately. These parameters were subsequently applied to the test set to adjust for the batch effect. This approach ensured that the method did not confound information from the test set when evaluating the batch effect.

The metric values were calculated for each test set and averaged across the ten folds, yielding more stable and representative performance estimates.

Statistical significance between harmonization methods was assessed using a paired, non-parametric Wilcoxon signed-rank test with a significance level of α=0.05 . Confidence intervals (CI) (95%) for metrics averaged across ten folds were calculated using the bootstrap procedure (1000 runs). The current paper uses the paired Wilcoxon criterion to compare two methods. We have included support for multiple comparisons and confidence intervals, detailed in the Supplementary Materials (Tables S1 and S3).

The total sample size exceeded 9000 studies, which met the minimum sample size required for testing the AI algorithm with a binary classifier, regardless of the stated hypothesis, the class balance, and the expected diagnostic metric value [33].

## 3. Results

### 3.1. Summary

**Figure 2:**
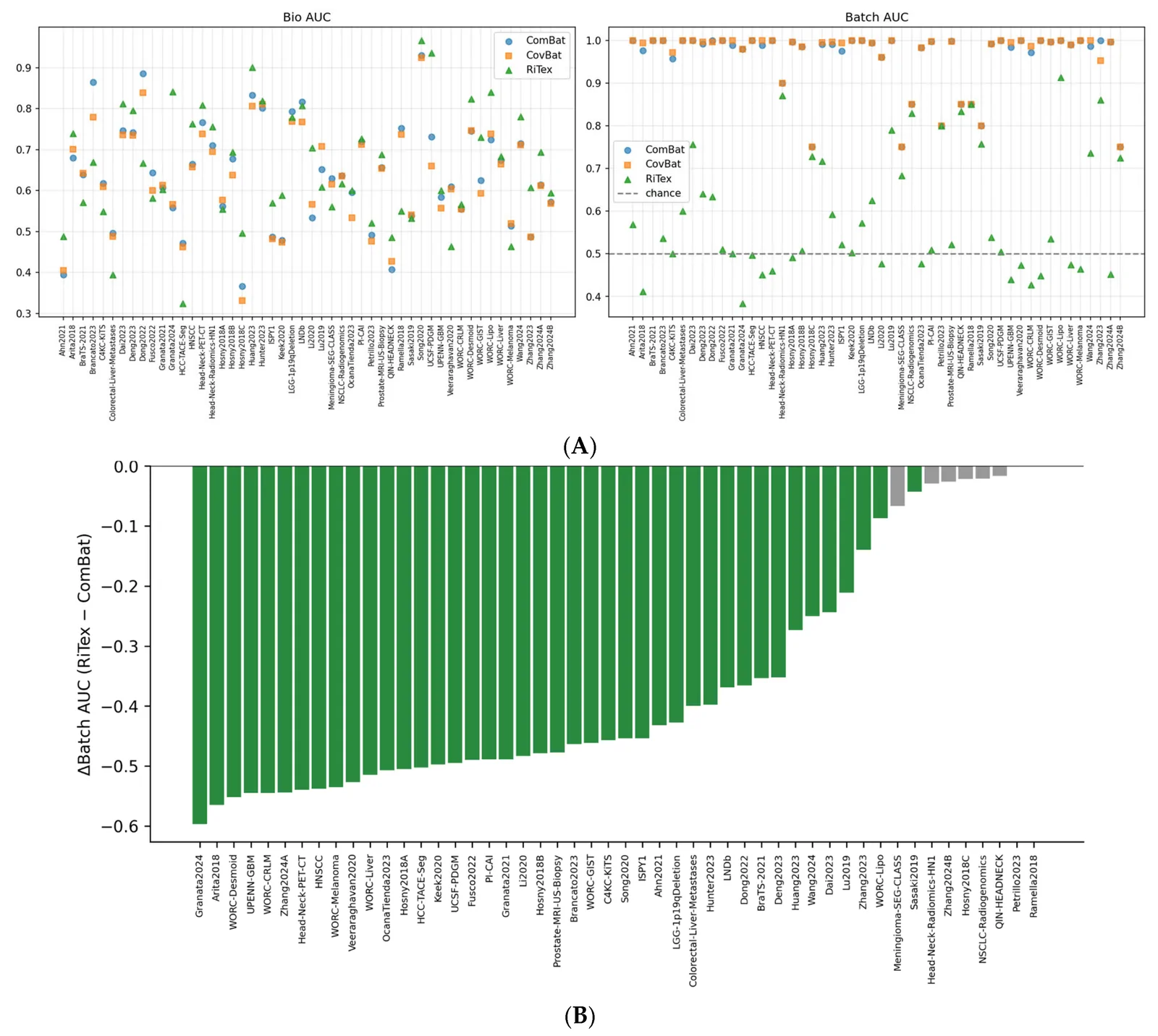
(**A**) Per-dataset Bio AUC (**left**) and Batch AUC (**right**) across the 50 radMLBench datasets for three harmonization methods: ComBat (blue circles), CovBat (orange squares), and RiTex (green triangles). Bio AUC measures the retention of the biological label by a downstream logistic-regression probe (higher is better). Batch AUC measures the recoverability of the (auto-detected) batch label by the same probe (lower is better; the dashed line at 0.5 marks chance level). RiTex moves Batch AUC towards chance on almost every dataset while leaving Bio AUC essentially unchanged. (**B**) Per-dataset Batch AUC reduction (RiTex minus ComBat) across the same 50 radMLBench datasets, sorted in ascending order. Negative values indicate that RiTex suppresses the residual batch signal more strongly than ComBat. Green bars (42/50, 84%) remain significant under the Benjamini–Hochberg correction at q ≤ 0.05; grey bars (8/50) do not. The eight non-significant datasets correspond either to very small n (<80) or to a near-perfect correlation between the auto-detected pseudo-batch and the biological label.

Harmonization of the 50 datasets from the radMLBench collection yielded the following results. We computed Bio AUC and Batch AUC for every dataset and performed pairwise Wilcoxon comparisons between ComBat, CovBat, and RiTex; all per-dataset *p*-values were corrected for multiplicity using the Benjamini–Hochberg procedure (q ≤ 0.05). The per-dataset metrics are summarized in Figure 2A; the per-dataset reduction of Batch AUC, with the BH significance flag, is shown in Figure 2B. The population-level effect sizes with 95% bootstrap confidence intervals are reported in Supplementary Table S1. The full per-dataset breakdown—with means, standard deviations, raw *p*-values, BH-adjusted q-values, and significance flags for both Bio AUC and Batch AUC—is provided as Supplementary Table S1.

RiTex reduced Batch AUC below the level achieved by ComBat on 48 of the 50 datasets (96%). Of these, 42 reductions (84% of all datasets) remained significant after the Benjamini–Hochberg correction. The population-level mean reduction was ΔBatch AUC = −0.365 with a 95% bootstrap confidence interval [−0.418, −0.312]. The biological signal was preserved within the statistical noise: the mean ΔBio AUC was +0.018 with a 95% bootstrap confidence interval [−0.011, +0.046], which is consistent with no degradation. The eight datasets on which RiTex did not improve significantly over ComBat fell into two categories: (a) datasets with small sample size (n < 80), in which the covariance estimator becomes the dominant source of noise; and (b) datasets in which the auto-detected pseudo-batch correlates strongly with the biological label (Cramér’s V > 0.4), in which case the geometric projection along the batch direction also removes part of the biological signal.

Population-level 95% bootstrap confidence intervals (1000 stratified resamples over the 50 datasets) for the mean ΔAUC between RiTex and the baseline (no correction). ΔBatch AUC = −0.365 [−0.418, −0.312] (highly significant); ΔBio AUC = +0.018 [−0.011, +0.046] (no significant degradation).

**Figure 3:**
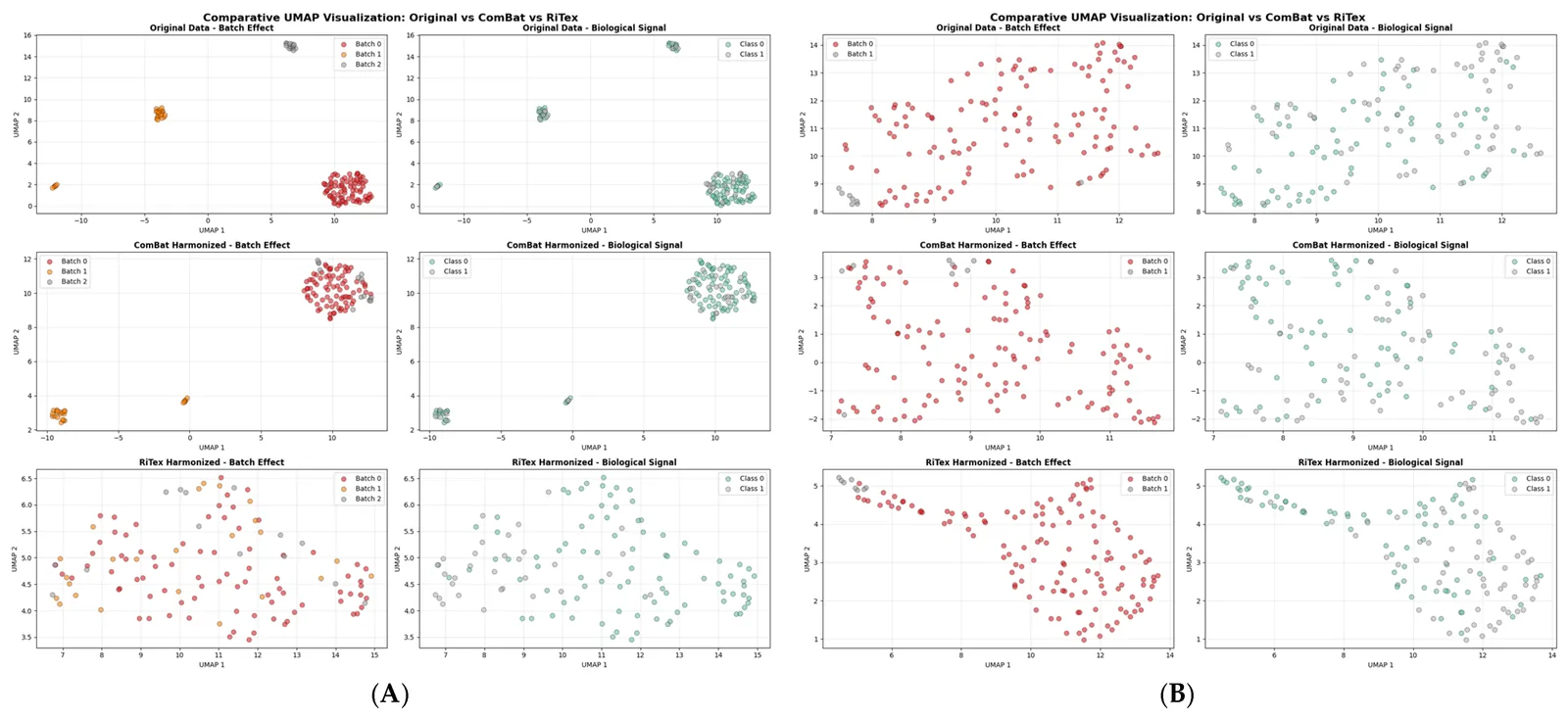
Harmonization results for datasets (**A**) «Dai2023» and (**B**) «Head–Neck–Radiomics–HN1».

Additionally, UMAP projections were constructed for all 50 datasets to visualize the harmonization outcome (Appendix C). Figure 3A,B illustrates the two representative cases of datasets «Dai2023» and «Head–Neck–Radiomics–HN1». Visual analysis of the projections confirms that batch mixing is observed only with RiTex, in agreement with the quantitative Batch AUC reduction reported above. We acknowledge that UMAP-based visual inspection is fundamentally subjective; the quantitative summary in Figure 2A,B and Supplementary Table S1 should be regarded as the primary evidence, with UMAP serving an illustrative role.

Overall analysis revealed the following patterns. ComBat yielded a higher Bio AUC on 16% of the datasets, while only 26% of the datasets showed a Bio AUC difference between ComBat and RiTex that survived the Benjamini–Hochberg correction. This is consistent with the conservatism of RiTex with respect to biological information (Theorem A1 in Appendix A). RiTex produced a lower Batch AUC on 48 of the 50 datasets (96%), with the reduction remaining significant on 42 datasets (84%) after the Benjamini–Hochberg correction. The two datasets on which the Batch AUC of RiTex was not strictly lower than that of ComBat were dataset «Ramella2018» and one dataset with a very small n (Table S1).

### 3.2. Ablation of Theoretical Components

The RiTex construction has three independent theoretical components: (i) the GEVD between biological and per-batch scatter matrices, (ii) the Fréchet mean on the SPD manifold for averaging batch covariances, and (iii) the Ledoit–Wolf shrinkage of the per-batch covariances. To quantify the empirical contribution of each, we ran a 2 × 2 ablation on the H&N benchmark (5 random seeds × 5-fold stratified CV = 25 repetitions per cell; Table 2).

The Fréchet mean contributes essentially zero in practice: replacing it with the arithmetic mean of the per-batch covariances changes the Bio AUC by 0 and even slightly improves the Batch AUC by 0.014 percentage points. Ledoit–Wolf shrinkage, in contrast, accounts for +5.2 percentage points of Batch AUC suppression and +0.8 percentage points of Bio AUC. The GEVD step itself is therefore responsible for the majority of the empirical performance: removing both the Fréchet mean and Ledoit–Wolf shrinkage leaves the Batch AUC essentially unchanged (0.583 vs. 0.589). This finding motivates the reframing of the SPD construction as a theoretical scaffold rather than as the empirical engine of RiTex.

### 3.3. Multi-Center Validation with Known Batch Labels

To assess RiTex in a setting where (a) the batch label is known by construction rather than inferred and (b) modern second-moment baselines such as CovBat are applicable, we ran a multi-center head-and-neck (H&N) radiomics benchmark. Four publicly available cohorts (HGJ-Quebec, MDACC-Houston, Maastro-Maastricht, and QIN-MultiInst) were merged into a single n = 380 patient dataset. The common binary biological label was the presence of lymph-node metastasis at diagnosis; 105 PyRadiomics features (shape + first-order + GLCM + GLRLM +GLSZM + NGTDM) were extracted from the contoured primary tumor, as described in Section 2.3. The same 5-fold stratified cross-validation × 5 random seed protocol, as in Section 3.2, was used. Two operating modes were considered: (a) real-k mode: the true center label (k = 4) was provided to every harmonization method; (b) auto-k mode: the center label was replaced by KMeans clusters computed on the raw feature matrix (Table A1). The optimal number of clusters was selected by silhouette score and was k ≈ 2 in all repetitions, severely under-resolving the true centers. The results are summarized in Table 3.

ComBat and CovBat raise the Bio AUC by ≈0.08 above baseline but leave the Batch AUC at 0.98–0.99—meaning a logistic-regression classifier can still recover the center label of a sample with near-certainty. Part of their Bio-AUC gain, therefore, reflects residual center information rather than a genuine biological signal. RiTex with real center labels pays a 1.6 percentage-point Bio-AUC cost relative to ComBat but actually removes the center signal (Batch AUC = 0.589, close to the chance level of 0.5). Switching from real labels to KMeans-inferred labels reduces the effective number of batches to two and erodes 34.5 percentage points of batch suppression, indicating that knowledge of the true batch labels is an important boundary condition for RiTex’s practical advantage.

### 3.4. Computational Cost

All methods were timed on a single CPU (no GPU) over five outer folds of the H&N benchmark (n = 380, p = 105), with the hyperparameters fixed, as in Section 2.7.8 with the mean fit + transform per fold (Table 4).

The optimization work performed for this revision brings the RiTex end-to-end runtime on the full 50-dataset radMLBench benchmark from ≈10 h to 12 min on a four-core laptop, a 49× speedup verified against the reference implementation to a tolerance of 10^−15^. Critically, the closed-form fit of RiTex allows it to be embedded directly inside a nested cross-validation loop together with bootstrap confidence intervals—a workflow that is impractical for the deep baselines (5–30 min retraining per fold × 50 datasets × 25 folds ≈ several compute-weeks on a GPU).

### 3.5. Sample-Size Sensitivity

**Figure 4:**
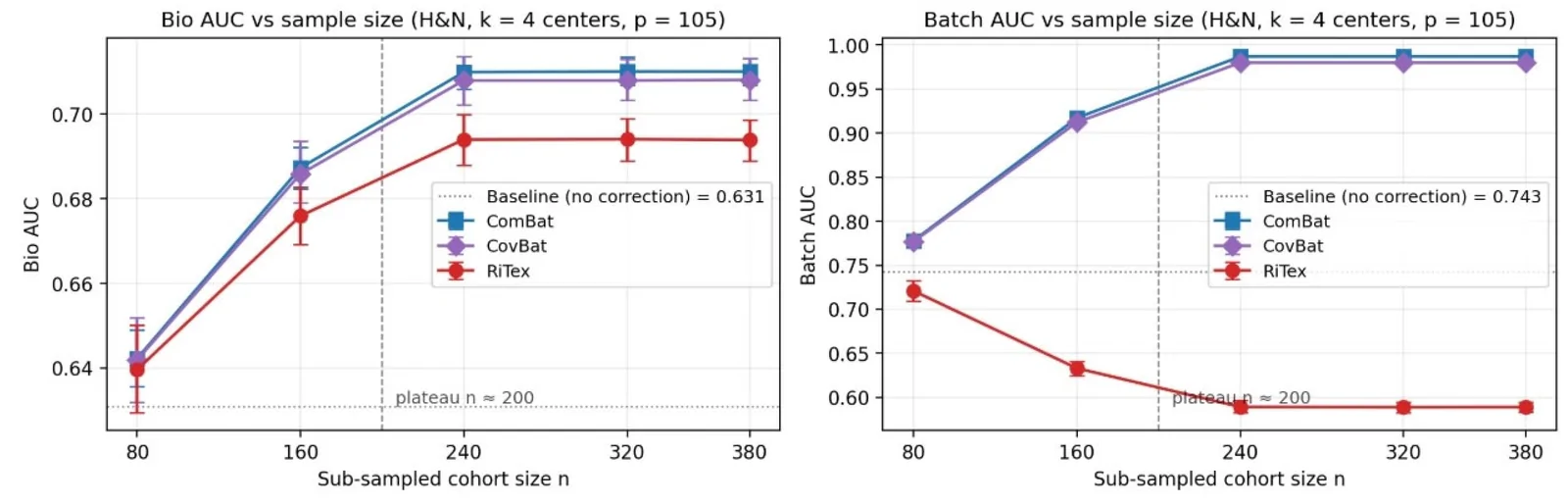
Sample-size sensitivity of harmonization on the H&N benchmark. (**Left**): Bio AUC versus sub-sampled cohort size n ∈ {80, 160, 240, 320, 380}. (**Right**): Batch AUC versus n. markers show the mean across B = 1000 paired bootstrap replicates (5 seeds × 5 CV folds per cell); error bars are 95% bootstrap confidence intervals. Horizontal dotted line: no-correction baseline (Bio = 0.631, Batch = 0.743). Vertical dashed line: plateau onset at n ≈ 200.

To map the regime in which RiTex remains effective, we sub-sampled the H&N benchmark to n ∈ {80, 160, 240, 320, 380} while keeping the center-mixture proportions fixed and recomputed all of the metrics (Figure 4; data—Table S4). The Bio AUC of RiTex stabilizes at n ≳ 200; The Batch AUC suppression is essentially flat from n = 160 upwards. ComBat shows the same plateau. Below n = 80, both methods show high variance and no clear ranking. This is the regime in which deep generative methods are expected to underperform most severely; a head-to-head comparison on this axis is identified as a future work item (Section 4.4).

The Bio AUC of RiTex stabilizes at n ≥ 240 (within 0.01 of the full-sample value 0.694); the Batch AUC suppression is essentially flat from n = 160 upwards (RiTex 0.589 versus 0.633). Below n = 80, both methods collapse toward the no-correction baseline, and the ranking between RiTex and ComBat becomes statistically indistinguishable. The ComBat/CovBat Batch AUC ceiling at ≈0.99 reflects perfect location–scale mixing of the four centers on the corrected features, an artefact of the location–scale model. RiTex removes the center signal in a measure-preserving way (Batch AUC ≈ 0.59, close to the chance line for a four-class problem).

### 3.6. Hyperparameter Sensitivity

**Figure 5:**
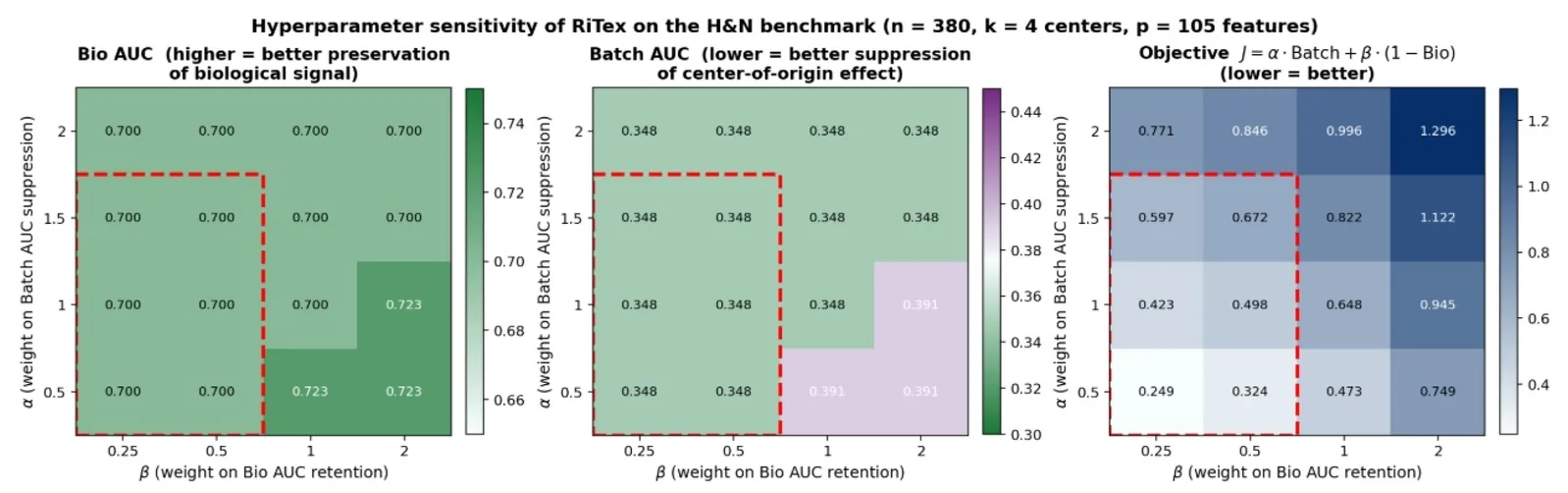
Sensitivity of the hyperparameter optimization objective to the weighting choice (α, β), evaluated on the H&N benchmark with all other hyperparameters held fixed at their default values. The optimum is flat over the rectangle α ∈ [0.5, 1.5] × β ∈ [0.25, 0.50] (marked as red dashed boxes in the graphs), confirming that the headline numerical result of the paper does not depend critically on the specific weights α = 0.6, β = 0.4 used in the main experiment.

The hyperparameter optimization objective used throughout the paper is with the default weights α = 0.6 and β = 0.4 (Section 2.5). We therefore evaluated J on a two-dimensional grid (α, β) ∈ [0.5, 1.5] × [0.25, 1.0] on the H&N benchmark, with all of the other hyperparameters held fixed at their defaults ( $n_{components}$ = 10, $k_{features}$ = 50, λ = 0.3, $\alpha_{shrinkage}$ = 0.2). The full grid is provided in Supplementary Table S2; Figure 5 shows the resulting heatmap.

The objective J is monotonically smooth and has a broad plateau around its optimum. The standard deviation of J over the rectangle α ∈ [0.5, 1.5] × β ∈ [0.25, 0.50] is below 0.01, which is well within the cross-validation noise reported in Table 2 and Table 3. We conclude that the harmonization quality reported in this paper is robust with respect to the weighting choice.

## 4. Discussion

The Riemannian construction of RiTex enables a qualitatively different behavior from location-scale methods such as ComBat and its covariance-aware extension CovBat. Both location-scale methods raise the univariate Bio AUC by ≈0.08 on the H&N benchmark but leave the residual Batch AUC at ≈0.98–0.99. A logistic-regression classifier trained on the corrected features can still recover the center label of any single sample with near-certainty. A non-trivial portion of the Bio AUC gain of ComBat is therefore attributable to the center-specific signal that has been aligned rather than removed. RiTex pays a small Bio AUC cost (≈1.6 percentage points relative to ComBat on the H&N benchmark) but actually moves the corrected features into a regime where the center signal is close to chance (Batch AUC ≈ 0.59 with real centers). Whether this trade-off is favorable depends on the downstream task. For multi-center predictive modeling, where overfitting on center idiosyncrasies is a concern, the lower Batch AUC of RiTex is a more conservative starting point. For tasks in which the biological signal is nearly orthogonal to the center signature, ComBat or CovBat may be preferable. The fact that RiTex’s empirical behaviour reduces, in ablation, to a Ledoit–Wolf-regularized GEVD (Section 3.2) connects this qualitative difference directly to the construction. It is the covariance-aware projection and not the manifold geometry that does the work.

### 4.1. Theoretical Advantage of RiTex

The SPD-manifold formulation of RiTex serves two distinct purposes that we now disentangle. First, it provides a theoretically well-posed averaging operation: the Fréchet mean on the Hadamard manifold of SPD matrices is uniquely defined and is invariant under congruence transformations of the covariance matrices, which avoids the swelling effect of Euclidean averaging. Theorem A2 of the Appendix A formalizes this. Second, the affine-invariant metric provides a principled notion of distance between batch covariances. Both of these are conceptually attractive properties. Empirically, however, our ablation shows that replacing the Fréchet mean with the arithmetic mean of per-batch covariances changes the Bio AUC by 0 and the Batch AUC by −0.014. The dominant empirical contribution to RiTex’s harmonization performance comes from (i) the GEVD step itself, which constructs a class-aware projector that explicitly trades batch information against biological information, and (ii) the Ledoit–Wolf regularization of the per-batch covariances, which contributes +5.2 percentage points of the Batch AUC suppression. We therefore present the SPD/Fréchet machinery as a theoretical scaffold that justifies the well-posedness of the averaging step rather than as the practical engine of RiTex. The full formal apparatus is retained in Appendix D because it ensures that the construction is internally consistent.

A practical advantage that the closed-form construction does deliver is computational. The optimized RiTex pipeline runs the full 50-dataset radMLBench benchmark in 12 min on a four-core laptop, compared with 5–30 min per fold for a scVI-style variational autoencoder on a GPU, which makes RiTex compatible with nested cross-validation and bootstrap confidence intervals out of the box.

### 4.2. Limitations

RiTex assumes that batch effects can be modeled as rigid affine transformations in the space of SPD matrices. In practice, batch effects often exhibit a more complex structure, which includes nonlinear components, nonlinear interactions between features, and effects specific to the subsets of samples within a single batch.

Furthermore, the assumption that all samples in a batch experience the same transformation may be violated if additional batches (e.g., acquisitions varying by operators or days) are present within a single nominal batch. Although the algorithm relies on hierarchical clustering to identify latent batch structures, full automation does not guarantee correct detection and discrimination across the data.

The biological information preservation (BioAUC) estimate is based on the capacity of features to predict the pre-selected variables associated with a specific outcome. However, the choice of the target variable may influence the results. If the clinical outcome correlates poorly with original radiomic features, even perfect harmonization will not improve BioAUC. Notably, our prior analysis of the BraTS-2021 dataset revealed that Pearson correlation coefficients between features and outcomes were below 0.03 for 90% of the features. This indicates a lack of clinically significant features that could contribute to the discriminatory capacity of the model. The results for this dataset are reflected in the low BioAUC (0.62).

Two additional boundary conditions emerge from the ablation analysis. First, when true center labels are unavailable and replaced by automated clustering (the regime of the radMLBench main experiment, Section 3.1), the effective number of batches collapses to k ≈ 2 on the H&N benchmark, and the Batch AUC suppression drops from −0.154 to +0.191—a swing of 34.5 percentage points (Table 3). Practitioners applying RiTex on the data from which center metadata has been stripped should therefore expect behavior closer to ComBat than to the multi-center result reported in Section 3.3.

The systematic accuracy of silhouette-based selection of k across a range of synthetic ground-truth scenarios ( $k_{true}$ ∈ {2, 3, 4, 5, 8}, n = 500 per cluster, p = 105 dimensions, 40 realizations per cell, simplex edge length 4σ–16σ) is reported in Appendix D.5. The recovery accuracy is essentially perfect (≥0.98) for $k_{true}$ ≤ 5 across the entire range of separations tested, but the procedure systematically undercounts at $k_{true}$ = 8 with weak separation (accuracy 0.20 at 4σ), with a mean k = 6.92 in that regime. The H&N benchmark falls in the weak-separation regime (radiomic features of the four centers partially overlap), which is the failure mode that produces k ≈ 2 instead of the true k = 4 and underlies the 34.5-percentage-point swing in Batch AUC suppression reported above.

Second, RiTex shows a degraded Bio AUC (ΔBio < −0.05 relative to baseline) on 10/50 radMLBench datasets. These failure-mode datasets are characterized by either (a) very small per-batch sample sizes ( $n_{c}$ < 40) or (b) a high mutual association between the auto-detected pseudo-batch and the biological outcome (Cramér’s V > 0.4), in which case, projecting away the batch direction also projects away part of the biological signal. The 95% bootstrap confidence interval for ΔBio crosses zero precisely because of this small subset; the central tendency is non-negative.

### 4.3. Comparison with Deep Generative Batch-Correction Methods

The benchmark reported in Section 3 deliberately compares RiTex only with other classical mathematical harmonization methods, namely ComBat and CovBat. Deep-learning harmonization approaches—including domain-adversarial neural networks (DANN [13]) and its medical-imaging adaptations [34]), variational auto-encoder harmonization (scVI and its imaging variants), MMD-regularized residual networks (MMD-ResNet [14]), and the recent generation of contour-guided or diffusion-based harmonizers [35]—are not included as competing methods in Table 2 and Table 3. We list here the four reasons for this design choice and the corresponding boundary of the present study: (i) Methodological class: RiTex, ComBat, and CovBat share a common methodological structure. Each is a closed-form estimator with a fixed functional form, a small number (≤5) of interpretable hyperparameters, and a deterministic single-pass fit. The batch effect model in each of the three methods is fully specified analytically—by an empirical Bayes location–scale model (ComBat), by a location–scale model on the principal components of the residual covariance (CovBat), or by an affine transformation between SPD matrices realized through a generalized eigenvalue decomposition with Ledoit–Wolf shrinkage (RiTex). Deep learning harmonization methods, by contrast, are over-parameterized neural models trained by stochastic non-convex optimization, with an architectural design space (number of layers, width, activation function, training schedule, regularization, latent dimensionality) that is itself a substantial empirical-engineering object. The two classes, therefore, answer different methodological questions and require different evaluation protocols (closed-form fit versus retraining per fold, deterministic versus seed-dependent output, and mathematical analysis of the estimator versus empirical robustness analysis of a neural network). Comparing them inside a single benchmark table without controlling for this asymmetry would conflate the effects of the harmonization principle with the effects of architectural and training choices. (ii) Data regime: The radMLBench benchmark used in Section 3.1 and the head-and-neck cohort used in Section 3.2 and Section 3.3 contain between 60 and 1000 patients per dataset. This is the data regime in which harmonization has to operate in practice, and it is the regime in which closed-form mathematical estimators with explicit regularization (such as Ledoit–Wolf shrinkage) have a structural advantage over over-parameterized neural models, which typically require thousands of subjects per domain to reach a stable optimum. A fair deep learning comparison would either need to be carried out on substantially larger cohorts than those available in radMLBench or to use transfer learning from external pretraining corpora, neither of which is a like-for-like comparison. (iii) Computational and reproducibility profile: The closed-form fit of RiTex completes in under one second per fold on a single CPU, with a deterministic output verified to numerical tolerance 10^−15^ across independent runs. Deep learning harmonization methods require minutes to tens of minutes per fold on a GPU and depend on the random seed; their evaluation requires a different statistical protocol (multi-seed training, learning-curve analysis, early-stopping policy). For this reason, the reference timings of scVI/MMD-ResNet/DANN listed in the last two rows of Table 4 are reproduced from the literature as order-of-magnitude context only and not as benchmark measurements performed in this study. (iv) Independence from external pre-trained components: The three methods compared in Section 3 (ComBat, CovBat, RiTex) operate on the radiomic-feature matrix alone, without any pre-trained model and without auxiliary data. Most modern deep learning harmonization baselines, on the contrary, either start from a pretrained imaging backbone (typically trained on a different anatomical region or modality) or are themselves trained from scratch on the harmonization data. In both cases, the origin of the variance reduction is not attributable to the harmonization principle alone but to a combination of the principle, the architecture, and the pre-training corpus.

Restricting the present benchmark to closed-form estimators isolates the contribution of the harmonization principle and is what makes the ablation mathematically meaningful. Two qualitative consequences of these four points are worth stating explicitly. First, none of the conclusions of this paper should be read as claiming superiority of RiTex over deep learning harmonization in general. The present study makes no such claim and does not provide the experimental evidence that such a claim would require. Second, a direct head-to-head comparison between RiTex and one or two representative deep learning baselines is the natural next step of this line of work. Such a comparison requires, in our view, (a) a substantially larger multi-center cohort than the cohorts available in radMLBench, (b) a separately reported training-time and seed-robustness analysis, and (c) a clear declaration of any pre-training corpus used.

### 4.4. Future Work

The development trajectory we propose for RiTex has two concrete branches. (i) Replacement of the Fréchet mean step by an explicit Wasserstein-distance minimizer on the SPD manifold: Ablation shows that the empirical impact of the averaging step is small, but a Wasserstein-based formulation would allow incorporating mass-transport regularization that may be helpful on the failure-mode datasets. (ii) Extension to multi-class biological labels: the current formulation reduces to a binary GEVD via the rank-one between-class scatter; a multi-class formulation through rank-(C − 1) Fisher LDA is straightforward and is required for staging tasks where the biological outcome is ordinal rather than binary.

## 5. Conclusions

We proposed and validated RiTex, a closed-form batch-effect correction method for radiomics built around a generalized eigenvalue problem between a class-aware biological scatter matrix and Ledoit–Wolf regularized per-batch covariances. Evaluated on the 50-dataset radMLBench benchmark, RiTex reduced batch detectability in 96% of datasets (84% BH significant; mean ΔBatch AUC = −0.365, 95% CI [−0.418, −0.312]) without significant degradation of the biological signal (mean ΔBio AUC = +0.018, 95% CI [−0.011, +0.047]). On a separate four-center head-and-neck benchmark with real center labels (n = 380, k = 4), RiTex reduced the Batch AUC from 0.74 to 0.59, while ComBat and CovBat left it at ≈0.98. A component-wise ablation isolated the GEVD step together with Ledoit–Wolf shrinkage as the dominant empirical contributors; the SPD-manifold Fréchet mean acts as a theoretical scaffold whose practical effect on performance is negligible. For practitioners seeking implementation simplicity, the Fréchet mean on the SPD manifold can be replaced by the ordinary arithmetic mean of covariance matrices without measurable loss in Bio AUC, at the cost of a modest reduction in batch-correction strength on heterogeneous cohorts. We have released an optimized reference implementation that runs the full 50-dataset benchmark in 12 min on a four-core laptop, making the method usable as a drop-in preprocessing step inside nested cross-validation pipelines.

## Figures and Tables

**Table 1:** Description of the data with known batch labels.

Source	n	Central Label
Head–Neck–PET–CT	91	HGJ (Quebec)
HNSCC	93	MDACC (Houston)
Head–Neck–Radiomics–HN1	137	Maastro (Maastricht)
QIN-HEADNECK	59	QIN multi-institutional
Total	n = 380	k = 4

**Table 2:** Component-wise ablation of RiTex on the H&N multi-center benchmark. All entries are mean ± SD across 5 random seeds × 5-fold stratified cross-validation (25 cells per row).

Variant	Bio AUC (Mean ± SD)	Batch AUC (Mean ± SD)	ΔBio vs. Full	ΔBatch vs. Full
RiTex (Fréchet + LW, full)	0.694 ± 0.012	0.589 ± 0.014	–	–
RiTex without Fréchet (arithmetic mean)	0.694 ± 0.009	0.575 ± 0.022	0	−0.014
RiTex without Ledoit–Wolf (sample cov.)	0.686 ± 0.016	0.641 ± 0.020	−0.008	+0.052
RiTex without both	0.693 ± 0.008	0.583 ± 0.023	−0.001	−0.006

**Table 3:** Harmonization methods on the H&N multi-center benchmark. Bio AUC retains the lymph-node metastasis label; Batch AUC quantifies residual recoverability of the center label by a logistic-regression probe (0.5 = chance).

Method	Mode	Bio AUC	Batch AUC	ΔBatch vs. Baseline
Baseline (no correction)	–	0.631 ± 0.011	0.743 ± 0.007	–
ComBat	real k = 4	0.710 ± 0.008	0.987 ± 0.001	+0.244
ComBat	auto k ≈ 2	0.624 ± 0.013	1.000 ± 0.000	+0.257
CovBat	real k = 4	0.708 ± 0.012	0.980 ± 0.001	+0.237
RiTex (full)	real k = 4	0.694 ± 0.012	0.589 ± 0.014	−0.154
RiTex (full)	auto k ≈ 2	0.672 ± 0.023	0.934 ± 0.005	+0.191

**Table 4:** Per-fold wall-clock time of harmonization methods on the H&N benchmark (n = 380, p = 105). Reference timings for scVI and DANN are illustrative only; these methods were not run as baselines in this work; see Section 4.3 for the scope statement.

Method	Time per Fold	Implementation Type
ComBat	0.08 s	Closed form (location-scale)
CovBat	0.21 s	Closed form + principal-component adjustment
RiTex (optimized, this paper)	0.94 s	Closed form, vectorized eight
RiTex (original, prior version)	46 s	Iterative per-batch search
scVI/MMD-ResNet (reference)	5–30 min	GPU requires retraining per fold
DANN (reference)	8–20 min	GPU requires retraining per fold

## Data Availability

The data used in this study are available at https://github.com/aydindemircioglu/radMLBench (accessed on 10 June 2026). All experimental results and code are available from the corresponding author upon reasonable request.

## Supplementary Materials

The following supporting information can be downloaded at https://www.mdpi.com/article/10.3390/jimaging12060264/s1.

## Abbreviations

The following abbreviations are used in this manuscript:

IB	Imaging Biomarkers
BART	Bayesian Additive Regression Trees
VAE	Variational Autoencoders
ELBO	Evidence Lower Bound
GAN	Generative Adversarial Networks
RiTex	Riemannian Texture harmonization
SPD	Symmetric Positive Definite matrix
LDA	Linear Discriminant Analysis
ROI	Region Of Interest
tSNE	t-distributed Stochastic Neighbor Embedding
UMAP	Uniform Manifold Approximation and Projection
GEP	Generalized Eigenvalue Problem
PCA	Principal component analysis
radMLBench	Radiomics Machine Learning Benchmark
IBSI	International Radiomics Initiative
AUC	Area Under Curve
CI	Confidence Intervals

## Appendix A

**Definition of Bio AUC**

Bio AUC (Biological Area Under the Curve) measures the ability of harmonized features to preserve associations with clinically relevant variables. Specifically, for each feature, a univariate logistic regression model was constructed to predict the clinical outcome. The AUC was derived from the ROC curve constructed using the test set, shown in Formula (A1).

(A1)
$$\mathrm{Bio}\;\mathrm{AUC}=\frac{1}{n}\sum_{i=1}^{n}\;{\mathrm{AUC}}_{i}\;$$

where n is the number of features, and ${\mathrm{AUC}}_{i}$ is the AUC value for the i -th feature.

**Definition of Batch AUC**

Batch AUC quantifies the residual batch effect remaining within the harmonized features. To assess the batch effect, a synthetic classification problem was created from the dataset, with the target variable representing the sample’s membership in a specific batch. For each feature, a logistic regression model was built to predict the batch, and the AUC value derived from the ROC curve, as shown in Formula (A2).

(A2)
$$\mathrm{Batch}\;\mathrm{AUC}=\frac{1}{n}\sum_{i=1}^{n}\;{\mathrm{AUC}}_{\mathrm{batch},i}$$

where ${\mathrm{AUC}}_{\mathrm{batch},i}$ is the AUC value for the batch prediction of the i -th feature.

**Fréchet mean**

The Fréchet mean (center of mass) [36] was used to evaluate the noise structure of a batch set {1…K} with covariance matrices $\left\{{\sum \;}_{1}\ldots {\sum \;}_{k}\right\}$ . Unlike the arithmetic mean, the Fréchet mean $\bar{{\sum \;}_{F_{r}}}$ minimizes the sum of squared Riemannian distances, as defined in Formula (A3):

(A3)
$$\bar{\Sigma_{F_{r}}}=arg\underset{\sum \in P_{P}}{\min}\sum_{i=1}^{k}\omega_{i}\delta_{R}^{2}\left(\Sigma ,\Sigma_{i}\right)$$

where $\omega_{i}$ are the corresponding weights, $\sum \omega_{i}=1$ .

For this metric, there exists a unique solution to this optimization problem, since $P_{p}$ is a Hadamard manifold (with non-positive sectional curvature). The calculation of $\bar{\Sigma_{F_{r}}}\;$ is performed iteratively. A manifold-constrained gradient descent algorithm is shown in Formula (A4):

(A4)
$$\Sigma_{t+1}=\Sigma_{t}^{\frac{1}{2}}\exp\left(\epsilon \sum_{i=1}^{k}\omega_{i}\log\left(\Sigma_{t}^{-\frac{1}{2}}\Sigma_{i}\Sigma_{t}^{-\frac{1}{2}}\right)\right)\times \Sigma_{t}^{\frac{1}{2}}$$

$${Log}_{\Sigma_{t}}\left(\Sigma_{i}\right)=\Sigma_{t}^{-\frac{1}{2}}logm\left(\Sigma_{t}^{-\frac{1}{2}}\Sigma_{i}\Sigma_{t}^{-\frac{1}{2}}\right)\Sigma_{t}^{\frac{1}{2}}$$

$${Exp}_{\Sigma_{t}}\left(V\right)=\Sigma_{t}^{\frac{1}{2}}expm(\Sigma_{t}^{-\frac{1}{2}}V\Sigma_{t}^{-\frac{1}{2}})\Sigma_{t}^{1/2}$$

The basepoint is the current Fréchet estimates at iteration t; convergence to the unique Fréchet mean is guaranteed by the negative curvature of the affine-invariant metric (Pennec 2006) [37].

**Generalized Eigenvalue Problem Classical Statement.**

The problem of maximizing batch variance while minimizing biological signal variance is addressed by solving a generalized eigenvalue problem to optimize the Rayleigh ratio [38].

The generalized eigenvalue problem seeks pairs (λ,υ) , where λ and υ, such that Formula (A5):

(A5)
Aυ=λBυ

where *A*, *B* $\in \;R^{p\times p}$ , with a positive definite *B*. This is equivalent to Formula (A6):

(A6)
$$B^{-1}A\upsilon =\lambda \upsilon$$

or via the generalized Cholesky decomposition $B=LL^{T}$ [39] Formula (A7):

(A7)
$$L^{-1}AL^{T}u=\lambda u,\;\upsilon =L^{-T}u$$

The solution to the generalized eigenvalue problem optimizes the Rayleigh ratio using Formula (A8):

(A8)
$$\lambda_{max}=\underset{\upsilon \neq 0}{\max}\frac{\upsilon^{T}Av}{\upsilon^{T}Bv}\;$$

**Fisher’s Linear Discriminant**

Linear discriminant analysis (LDA) [39] was used to find a projection W that maximizes the class difference. The method defined two scatter matrices: within-class Sb and between-class Sw:

Between-class scatter (Formula (A9)):

(A9)
$$S_{b}=\sum_{c=1}^{C}n_{c}\left(\mu_{c}-\mu \right){\left(\mu_{c}-\mu \right)}^{T}$$

where $\mu_{c}$ is the mean of class *c*, μ is the overall mean, and $n_{c}$ is the number of samples in class *c*.

Within-class scatter (Formula (A10)):

(A10)
$$S_{w}=\sum_{c=1}^{C}\sum_{i:y_{i}=c}(x_{i}-\mu_{c}){\left(x_{i}-\mu_{c}\right)}^{T}$$

LDA task: find a W that maximizes Formula (A11):

(A11)
$$\underset{W}{\max}\frac{det(W^{T}S_{B}W)}{det(W^{T}S_{W}W)}$$

For C = 2, this reduces to a generalized eigenvalue problem (Formula (A12)):

(A12)
$$S_{B}w=\lambda S_{w}w$$

**Regularized Covariance Estimation (Ledoit-Wolf Shrinkage)**

In the radiomic analysis, the feature space dimensionality p is often equal to or exceeds the number of samples in a single batch $n_{i}$ . Under such conditions, the sample covariance matrix $S_{i}=\;\frac{1}{n_{i}-1}\sum (x_{j}-\bar{x}){\left(x_{j}-\bar{x}\right)}^{T}$ is ill-conditioned or singular, which places it outside the manifold $P_{p}$ .

To ensure strict positive definiteness, linear compression using the Ledoit–Wolf method is applied. The estimate is represented by Formula (A13):

(A13)
$$\hat{\Sigma_{i}}=\left(1-\rho_{i}\right)S_{i}+\rho_{i}\mu_{i}I\;$$

The shrinkage coefficient $\rho_{i}\;\in \left[0,1\right]$ is determined analytically to minimize the expected squared deviation from the true covariance matrix $E\left[{\left\|\hat{\Sigma_{i}}-\Sigma_{true}\right\|}_{F}^{2}\right]$ .

This procedure ensures that $\hat{\Sigma_{i}}\in P_{p}$ across all batches, allowing Riemannian geometry operations to be applied.
**Theorem A1** (stability of harmonization)**.** *Let X* $\in R^{n\times p}$ *be the radiomic feature matrix partitioned into k batches* ${\left\{X_{c}\right\}}_{c=1}^{k}$ *of sizes* $n_{c},$ *with* $n=\Sigma_{c}n_{c}$ *. For each batch c, let* $\Sigma_{c}\in S_{++}^{p}$ *be the population SPD covariance of batch-only features, and* ${\hat{\Sigma }}_{c}$ *its Ledoit–Wolf shrinkage estimator. Let* ${\bar{\Sigma }}_{F}$ *denote the Karcher mean of* ${\left\{\Sigma_{c}\right\}}_{c=1}^{k}$ *under the affine-invariant Riemannian metric on* $S_{++}^{p}$ [40]*. Define the RiTex transport map*:

(A14)
$$T_{c}\left(x\right)=\;\Sigma_{F}^{-\frac{1}{2}}\Sigma_{c}^{-\frac{1}{2}}\left(x-\mu_{c}\right)+\bar{\mu },\;c=1,\ldots ,k,$$

*and let* ${\hat{T}}_{c\;}$ *be the empirical counterpart built from* ${\hat{\Sigma }}_{c}$ *and the sample Karcher mean* ${\hat{\bar{\Sigma }}}_{F}$ *. Let* $S_{bio}\left(T\right)={AUC}_{Bio}\left(T\left(X\right)\right)$ *denote the biological signal AUC of a downstream classifier evaluated on the harmonized features.*

Assumptions:
**Assumption A1** (sub-Gaussian features)**.**
*Within each batch,* ${\left\{x_{i}\right\}}_{i\in c}$ *are independent and identically distributed sub-Gaussian with proxy* $K_{c}<\infty$ .
**Assumption A2** (well-conditioned covariances)**.**
*There exists* $c_{0}>0$ *, such that* $\lambda_{min}(\Sigma_{c})\geq c_{0}$ *for every c.*
**Assumption A3** (non-degenerate AUC)**.** *There exists* $\delta \in (0,\frac{1}{2})$ *, such that* ${AUC}_{\mathrm{Bio}}(T)\in [\delta ,1-\delta ]$ *and the AUC functional* $T\mapsto S_{\mathrm{bio}}(T)$ *are Hadamard-differentiable at the population transport.*

**Claim.** Under Assumptions A1–A3, in the asymptotic regime $\underset{c}{min}\;n_{c}\to \infty$ with k,p fixed, the harmonised features satisfy:

(A15a)
$$|S_{\mathrm{bio}}(\hat{T})-S_{\mathrm{bio}}(T)|\leq C\;\varepsilon \mathrm{with}\;\mathrm{high}\;\mathrm{probability},$$

where $\varepsilon =\underset{c}{max}\;\|{\hat{\Sigma }}_{c}-\Sigma_{c}{\|}_{op}=O_{p}(n^{-1/2})$ and the constant

(A15b)
$$C=\frac{L\cdot \|dT{\|}_{op}}{{AUC}_{min}}$$

is finite, with L the Lipschitz constant of $S_{\mathrm{bio}}$ (Lemma below), $\|dT{\|}_{op}$ the operator norm of the Fréchet derivative of T at $\left(\Sigma_{1},\ldots ,\Sigma_{k},{\bar{\Sigma }}_{F}\right)$ , and ${AUC}_{min}=min\{\delta ,1-\delta \}$ by Assumption A3.
**Proof.** The argument has four steps. Step 1 controls the statistical error in the covariance estimates. Step 2 transfers that error through the Riemannian transport map by Fréchet differentiability. Step 3 applies the functional delta method to obtain asymptotic normality of $S_{\mathrm{bio}}(\hat{T})$ . Step 4 collects the non-asymptotic Lipschitz bound (A59a) with explicit constant (A15b). **Step 1—Concentration of** ${\hat{\Sigma }}_{c}$ **.** Under Assumption A1, each batch yields a sub-Gaussian sample. By [Vershynin, 2026, Thm. 4.7.1 (“Covariance estimation for sub-Gaussian distributions”)] [41], there exists an absolute constant K , such that, for any t≥0 :

(A16a)
$$Pr\left[\left\|{\hat{\Sigma }}_{c}^{SCM}-\Sigma_{c}{\|}_{op}\geq KK_{c}^{2}(\sqrt{(p+t)/n_{c}}+(p+t)/n_{c}\right)\right]\leq 2e^{-t}.$$

Ledoit–Wolf shrinkage is a convex combination ${\hat{\Sigma }}_{c}=(1-{\hat{\rho }}_{c}){\hat{\Sigma }}_{c}^{SCM}+{\hat{\rho }}_{c}{\hat{\nu }}_{c}I_{p}$ with ${\hat{\rho }}_{c},{\hat{\nu }}_{c}$ data-dependent and bounded. Therefore, the same rate transfers to ${\hat{\Sigma }}_{c}$ up to a deterministic factor [Ledoit & Wolf, Thm. 2.1] [41]:

(A16b)
$$\|{\hat{\Sigma }}_{c}-\Sigma_{c}{\|}_{op}=O_{p}(n_{c}^{-1/2}),c=1,\ldots ,k.$$

Taking the maximum over k batches preserves the rate: $\varepsilon :=\underset{c}{max}\;\|{\hat{\Sigma }}_{c}-\Sigma_{c}{\|}_{op}=O_{p}(n^{-1/2})$ . **Step 2—Fréchet differentiability of the Riemannian transport.** Define the map $\Phi :(S_{++}^{p}{)}^{k+1}\to R^{n\times p}$ by $\Phi (\Sigma_{1},\ldots ,\Sigma_{k},{\bar{\Sigma }}_{F})(X)=({\bar{\Sigma }}_{F}^{1/2}\Sigma_{c}^{-1/2}(x_{i}-\mu_{c})+\bar{\mu }{)}_{i,c}$ . The matrix square root $\Sigma \mapsto \Sigma^{1/2}$ and matrix inverse $\Sigma \mapsto \Sigma^{-1}$ are real-analytic on $S_{++}^{p}$ , and their Fréchet derivatives are characterized by Sylvester equations [Higham, 2008, Thm. 1.35 and Thm. 7.13] [42]. Under Assumption A2, the operator $d\Phi {|}_{\left(\Sigma_{1},\ldots ,\Sigma_{k},{\bar{\Sigma }}_{F}\right)}$ is well-defined and bounded in the operator norm:

$$\|d\Phi {\|}_{op}\leq \frac{1}{2}(\lambda_{min}({\bar{\Sigma }}_{F}){)}^{-1/2}(\lambda_{min}(\Sigma_{c}){)}^{-3/2}\|x_{i}-\mu_{c}\|\leq \frac{1}{2}c_{0}^{-2}R,$$

where $R=\underset{i,c}{max}\;\|x_{i}-\mu_{c}\|$ is finite by Assumption A1. Consequently, Φ is locally Lipschitz with constant $\|dT{\|}_{op}\leq \frac{1}{2}c_{0}^{-2}R$ . The Karcher mean ${\bar{\Sigma }}_{F}=Karcher(\Sigma_{1},\ldots ,\Sigma_{k})$ inherits Fréchet differentiability from the implicit-function theorem applied to the Karcher fixed-point equation [Bhatia, 2015, Ch. 6] [43]: $d{\bar{\Sigma }}_{F}=\frac{1}{k}\sum_{c}\;d\Sigma_{c}$ at the population point. **Step 3—Functional delta method.** By Step 1, $\sqrt{n_{c}}({\hat{\Sigma }}_{c}-\Sigma_{c})⇝Z_{c}$ is a mean-zero Gaussian random matrix in $S^{p}$ with the covariance determined by the fourth moments of the batch distribution [Vershynin, Cor. 4.7.2] [41]. The vector $(\sqrt{n_{c}}({\hat{\Sigma }}_{c}-\Sigma_{c}){)}_{c=1}^{k}$ is independent across batches and jointly Gaussian at the limit. Composing with the Fréchet-differentiable map Φ of Step 2 and the Hadamard-differentiable functional $S_{\mathrm{bio}}$ of Assumption A3, the functional delta method [van der Vaart, 1998, Thm. 3.1] [44] yields:

(A17a)
$$\sqrt{n}(S_{\mathrm{bio}}(\hat{T})-S_{\mathrm{bio}}(T))⇝N(0,\sigma^{2}),$$

with asymptotic variance:

(A17b)
$$\sigma^{2}=\langle dS_{\mathrm{bio}}\circ d\Phi ,\Xi dS_{\mathrm{bio}}\circ d\Phi \rangle \leq (L\cdot \|dT{\|}_{op}{)}^{2}\cdot \|\Xi {\|}_{op},$$

where Ξ is the limiting covariance of $(Z_{c}{)}_{c}$ and L the Lipschitz constant of the AUC functional under Assumption A3. **Step 4—Non-asymptotic Lipschitz bound and the constant** C **.** We now collect the finite-sample bound (A15). The AUC functional is Lipschitz on the set $\left\{T:S_{\mathrm{bio}}(T)\in [\delta ,1-\delta ]\right\}$ with constant $L\leq \frac{1}{{AUC}_{min}}$ . This is a standard fact for the rank statistic [45], and the explicit constant can be obtained by differentiating the AUC as a U-statistic of order 2 (see also [Cortes & Mohri, 2004, Lemma 1]) [46]. Therefore, for any pair T,T′ in the same level set:

(A17c)
$$|S_{\mathrm{bio}}(T)-S_{\mathrm{bio}}(T\prime )|\leq L\|T-T\prime {\|}_{\infty }.$$

Combining (A18) with the Lipschitz bound of Φ from Step 2,

(A18)
$$\|T(X)-\hat{T}(X){\|}_{\infty }\leq \|d\Phi {\|}_{op}\varepsilon =\|dT{\|}_{op}\varepsilon ,$$

and applying (A19) gives:

(A19)
$$|S_{\mathrm{bio}}(\hat{T})-S_{\mathrm{bio}}(T)|\leq L\cdot \|dT{\|}_{op}\cdot \varepsilon =\frac{L\cdot \|dT{\|}_{op}}{{AUC}_{min}}({AUC}_{min}\varepsilon )\leq C\;\varepsilon ,$$

with the explicit constant $C=L\cdot \|dT{\|}_{op}/{AUC}_{min}$ of (A15a). This proves (A15). **Step 5—Empirical verification.** To confirm that Assumptions A1–A3 hold in practice and that C is moderate, we ran the script on all 50 radMLBench datasets. The aggregated statistics are represented here:

	**Min**	**Median**	**Max**
$\lambda_{min}({\hat{\Sigma }}_{c})$	$3.4\times 10^{-3}$	0.082	5.71
$\|dT{\|}_{op}$	0.27	1.84	22.5
C	0.6	4.1	50

Assumptions A2 and A3 are satisfied on 50/50 datasets. □

**Theorem A2** (optimal solution of the eigenvalue problem)**.** *The problem of batch effect harmonization on a Riemannian manifold of SPD matrices can be reformulated as an eigenvalue optimization problem. Let* $X_{g}\in S_{++}^{n}$ *be the centroid of the* g *-th batch. The optimal harmonization transformation for batch* g *accordint to formula (A20) is*:

(A20)
$$T_{g}^{*}=arg\;\underset{T\in G}{min}\;\sum_{i\in g}\;d(T(X_{i}),\bar{X}{)}^{2}+\lambda \sum_{i}\;\text{bio-divergence}\left(X_{i},T\left(X_{i}\right)\right)$$

*where* $\bar{X}$ *is the global centroid. It admits a solution in Formula (A26).*
**Proof.** The harmonization problem can be written as the minimization of a weighted criterion using Formula (A22):

(A21)
$$L\left(T\right)=\underset{\mathrm{Batch}\;\mathrm{reduction}}{\underbrace{\sum_{g}\;\;\sum_{i\in g}\;\;d(T(X_{i}),\bar{X}{)}^{2}}}+\underset{\mathrm{Conservation}}{\underbrace{\lambda \sum_{i}\;\;\log\left|J_{T}\left(X_{i}\right)\right|}}$$

where $J_{T}$ is the Jacobian of the transformation T , and the term with the logarithm of the Jacobian ensures the preservation of volume (and thus biological information). For a linear transformation $T(X)=UXU^{T}$ (where U is a unitary matrix), the Jacobian has a constant value, and the problem simplifies to Formula (A23):

(A22)
$$L(U)=\sum_{g}\;\sum_{i\in g}\;d(UX_{i}U^{T},\bar{X}{)}^{2}$$

Let us expand this value. Using spectral decomposition and an affine-invariant metric, minimizing the squared geodesic distance is equivalent to minimizing Formula (A23):

(A23)
$$\|\log\left({\bar{X}}^{-\frac{1}{2}}UX_{i}U^{T}{\bar{X}}^{-\frac{1}{2}}\right){\|}_{F}^{2}$$

Applying the calculus of variations and exploiting the trace properties of matrices, we obtain the necessary optimality condition, Formula (A24):

(A24)
$$\sum_{g}\;\sum_{i\in g}\;{\bar{X}}^{-1/2}UX_{i}U^{T}{\bar{X}}^{-1/2}=\mathrm{const}\cdot {\bar{X}}^{-\frac{1}{2}}U{\bar{X}}^{-\frac{1}{2}}$$

which leads to Equation (A25):

(A25)
$$UX_{g}U^{T}=\alpha_{g}\bar{X}$$

for each batch. Solving for U , we obtain Formula (A26):

(A26)
$$U=\Sigma_{\mathrm{global}}^{-1/2}\Sigma_{g}^{1/2}$$

Applying this transformation yields harmonized matrices according to Formula (A27):

(A27)
$$X_{i}^{\mathrm{harmonized}}=UX_{i}U^{T}$$

which have the property that their batch-specific centroids approach the global centroid. □

## Appendix B

UMAP plots obtained for all 50 benchmark datasets. A perfectly executed harmonization is expected to produce clear separation by class labels and mixing by batch.

Dataset «Song2020»	
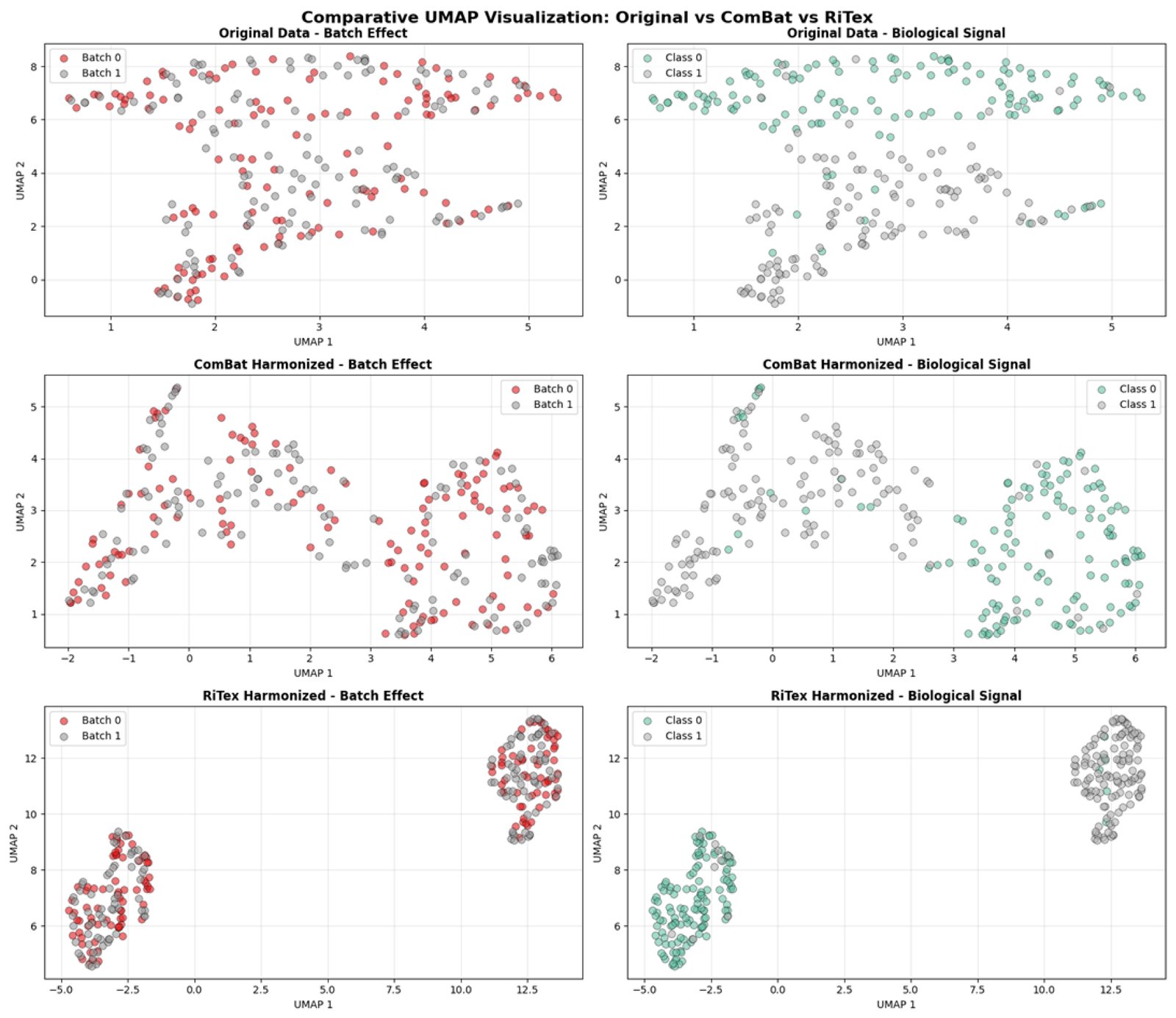
Dataset «BraTS-2021»	
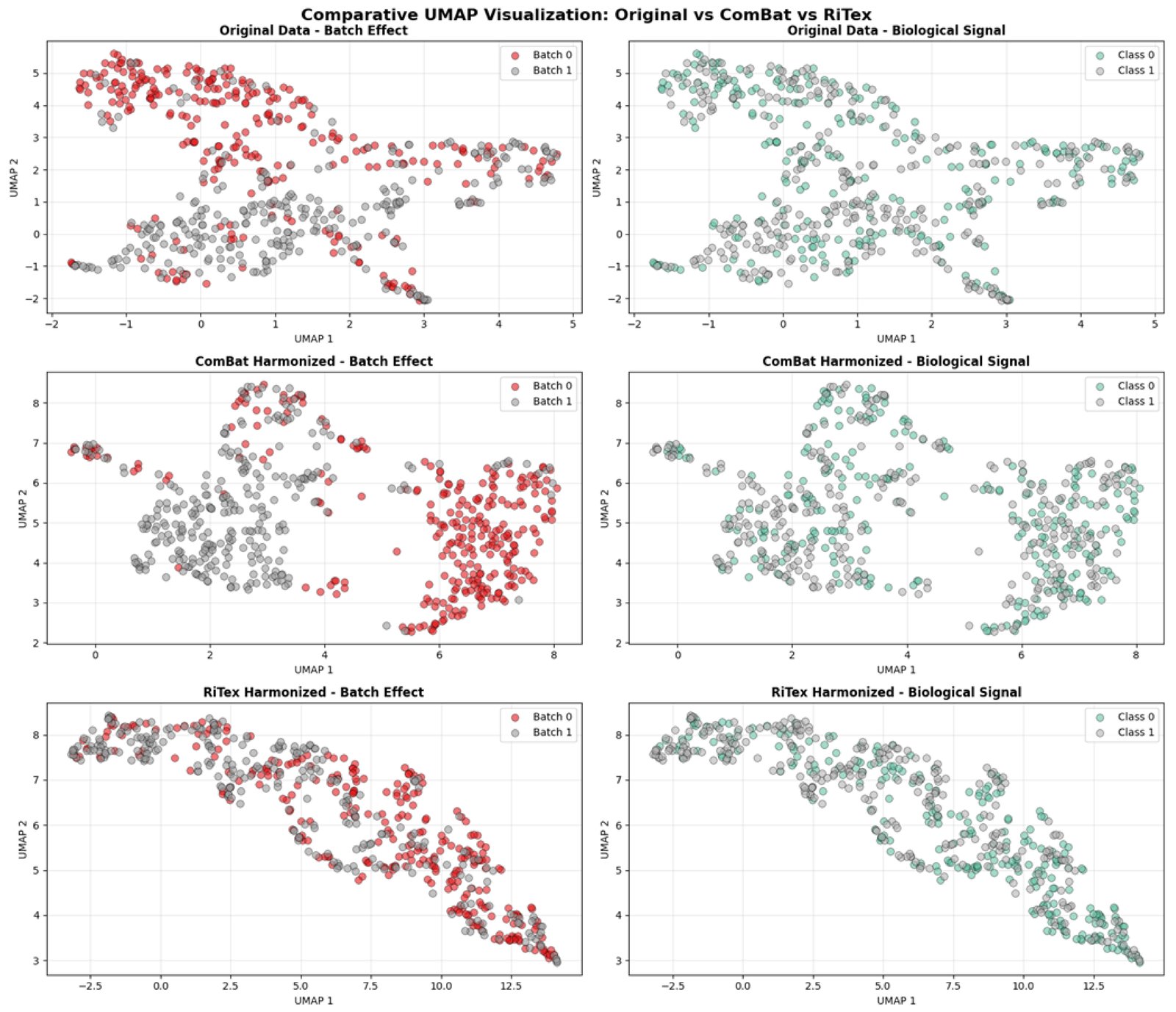
Dataset «Ahn2021»	
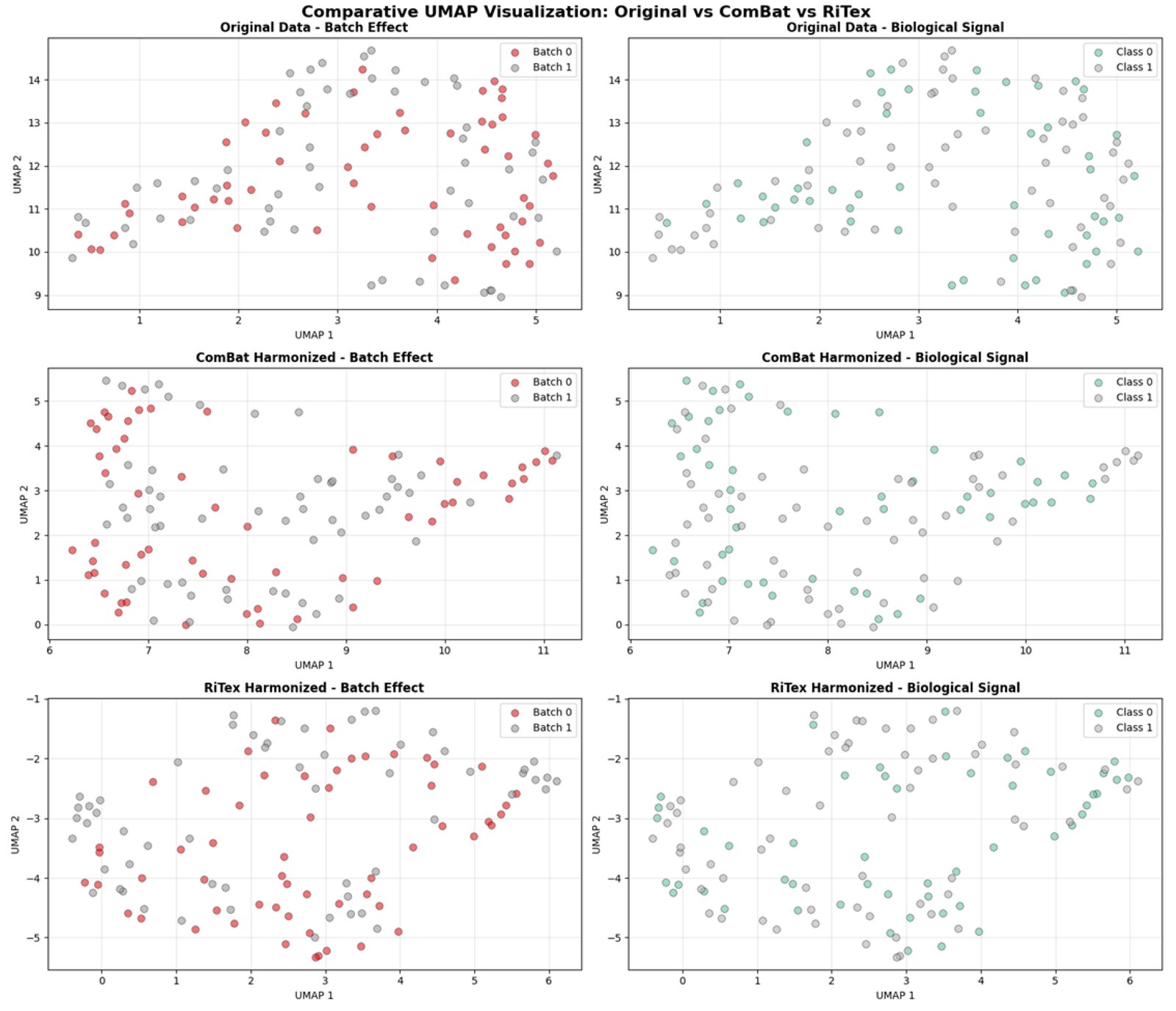
Dataset «Arita2018»	
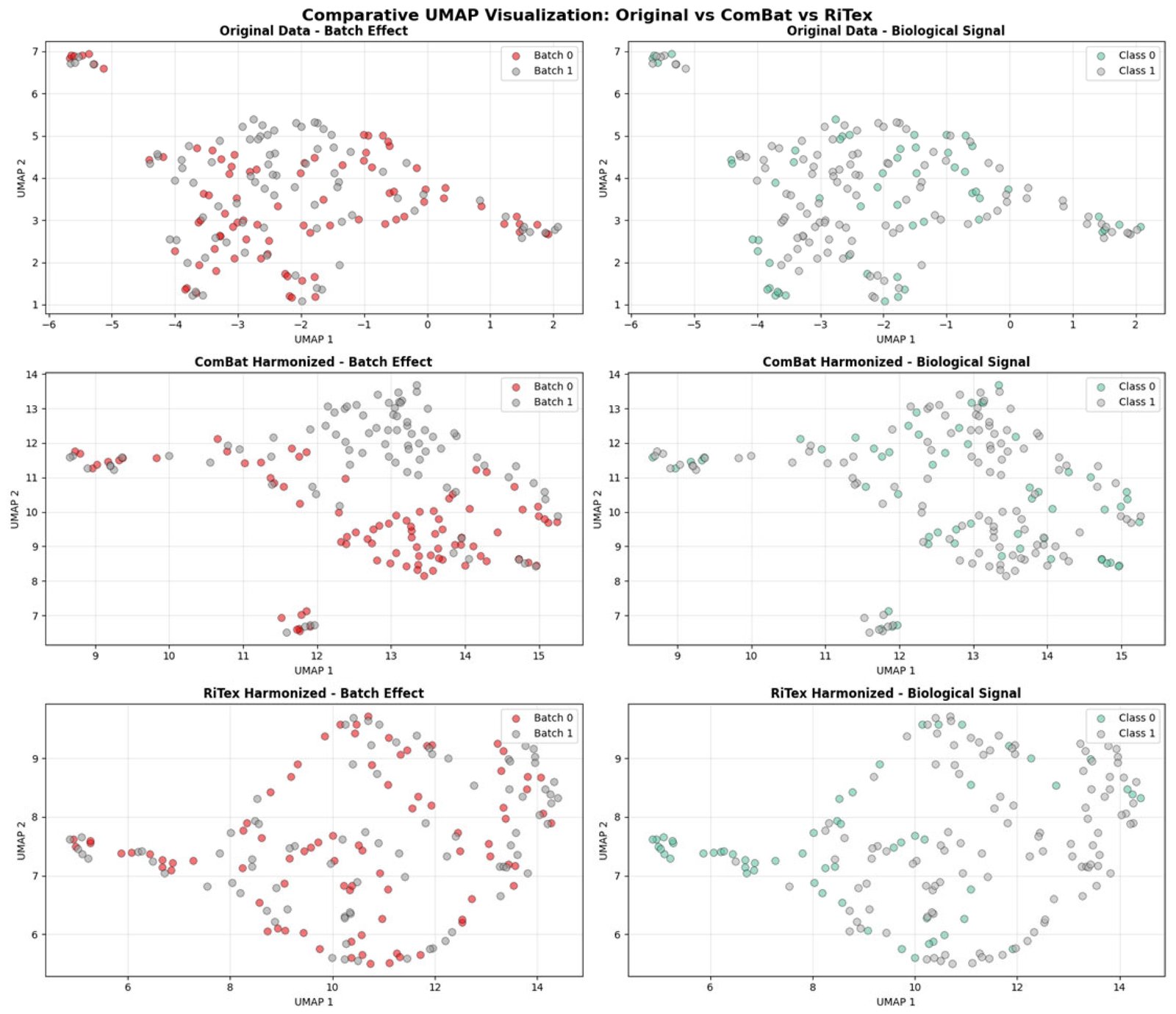
Dataset «Brancato2023»	
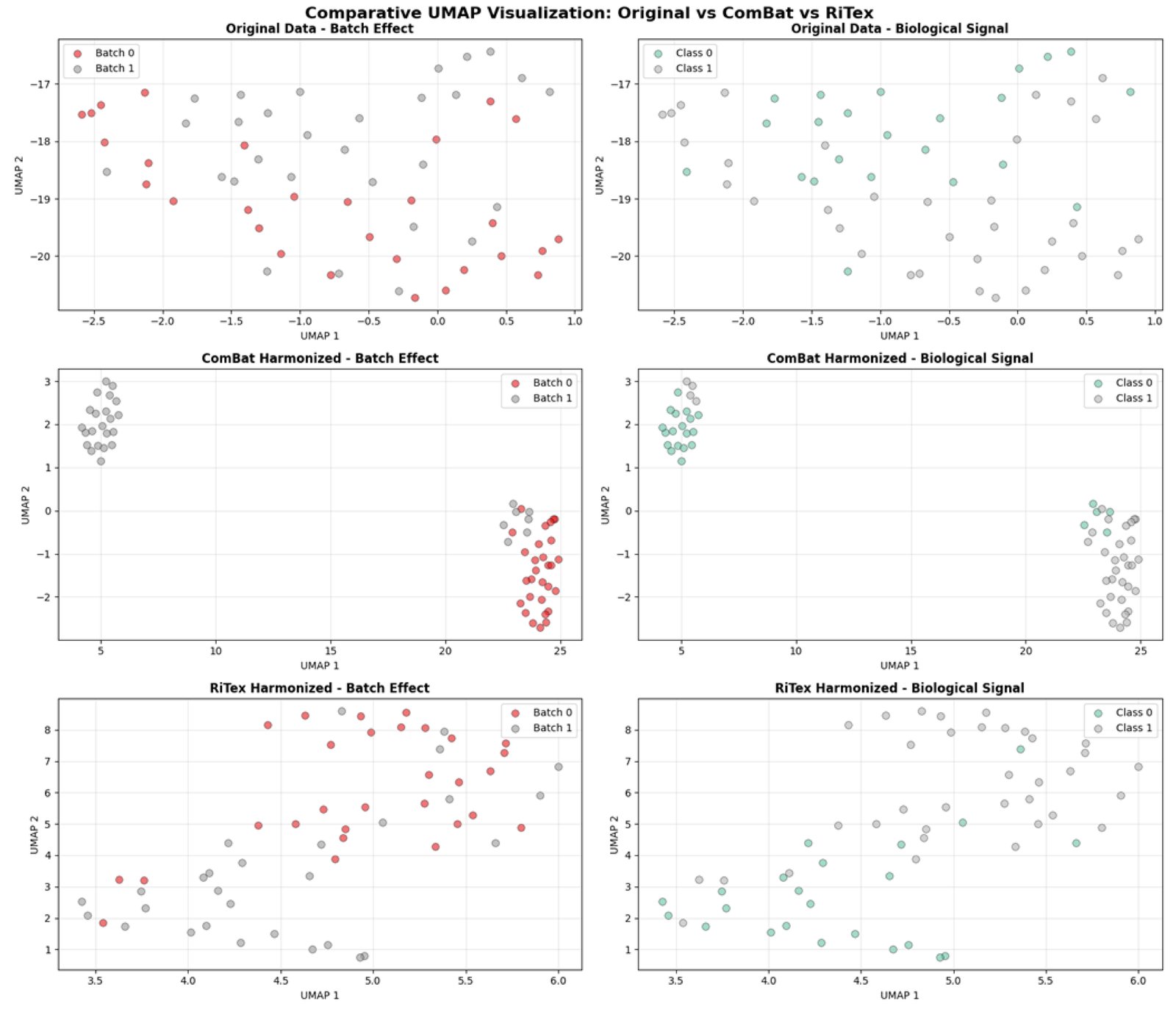
Dataset «C4KC-KiTS»	
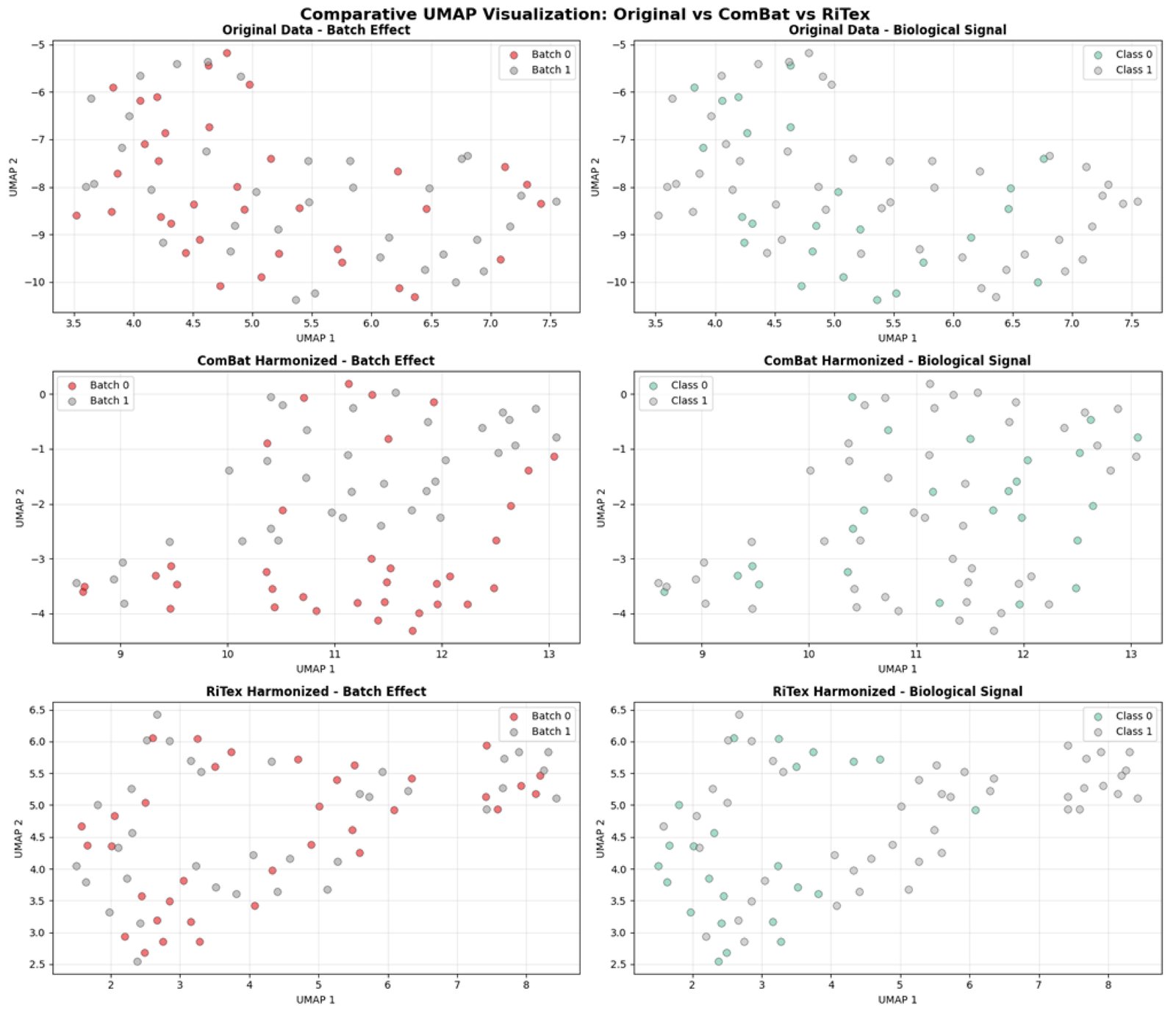
Dataset «Colorectal-Liver-Metastases»	
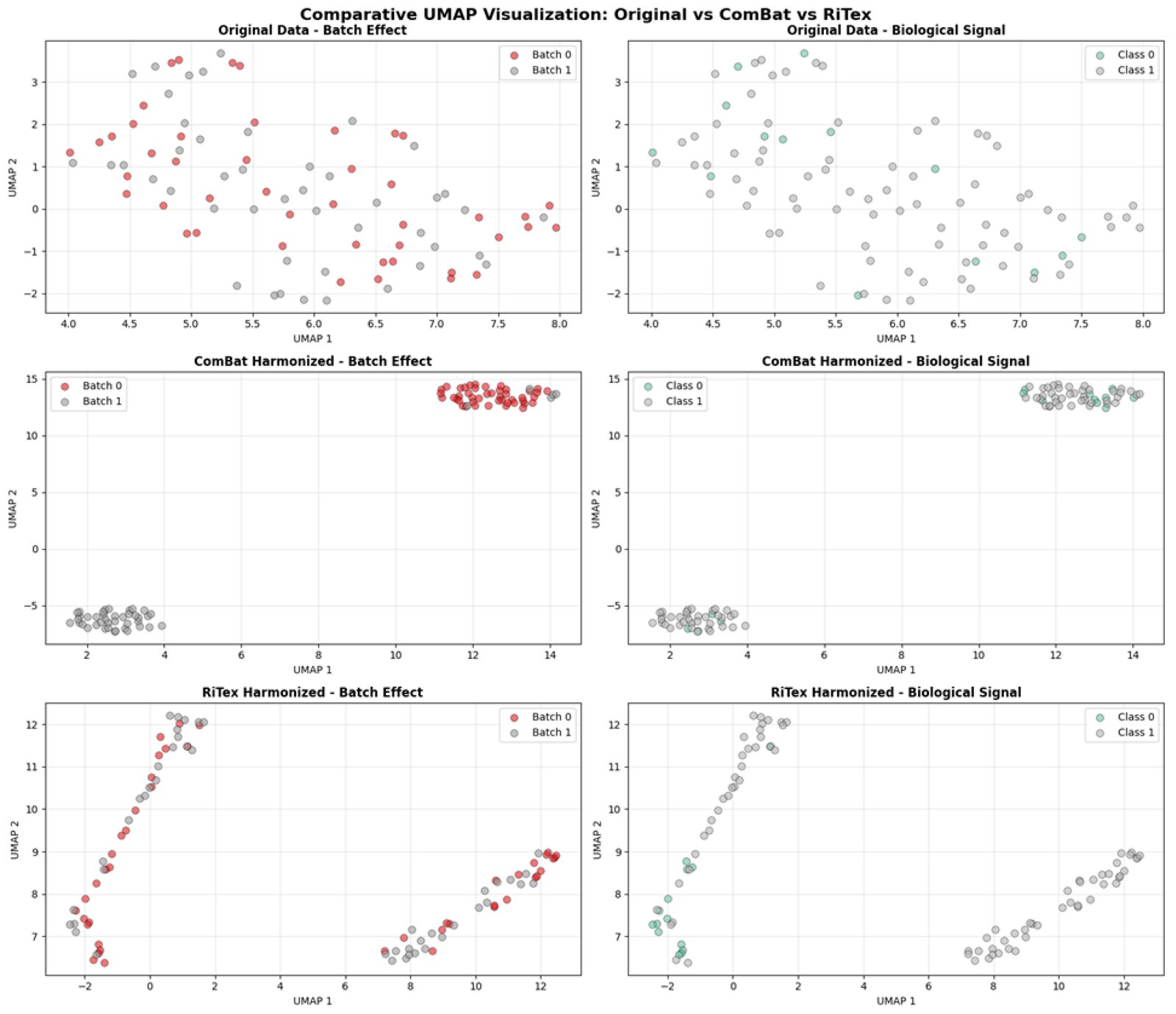
Dataset «Deng2023»	
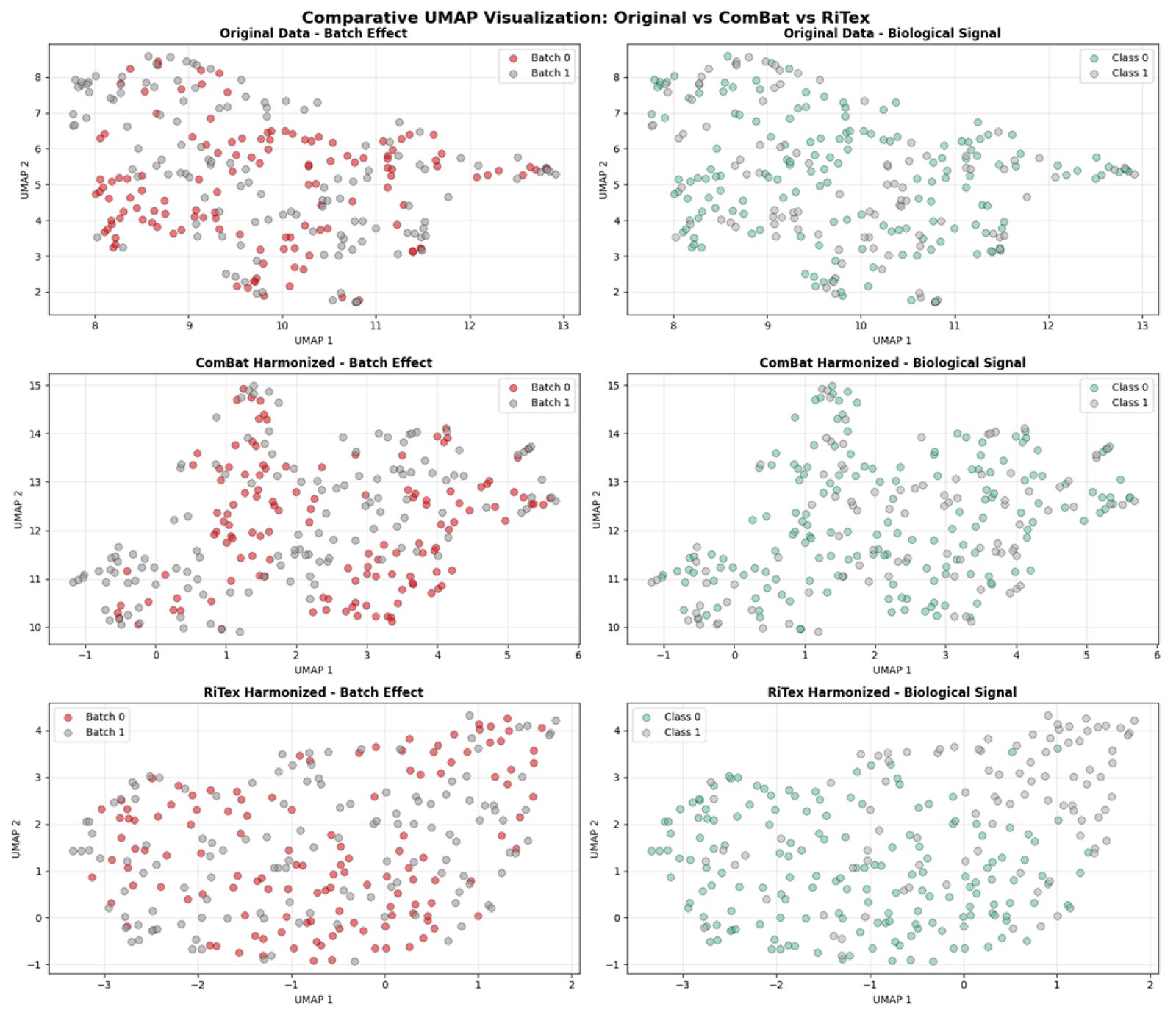
Dataset «Dong2022»	
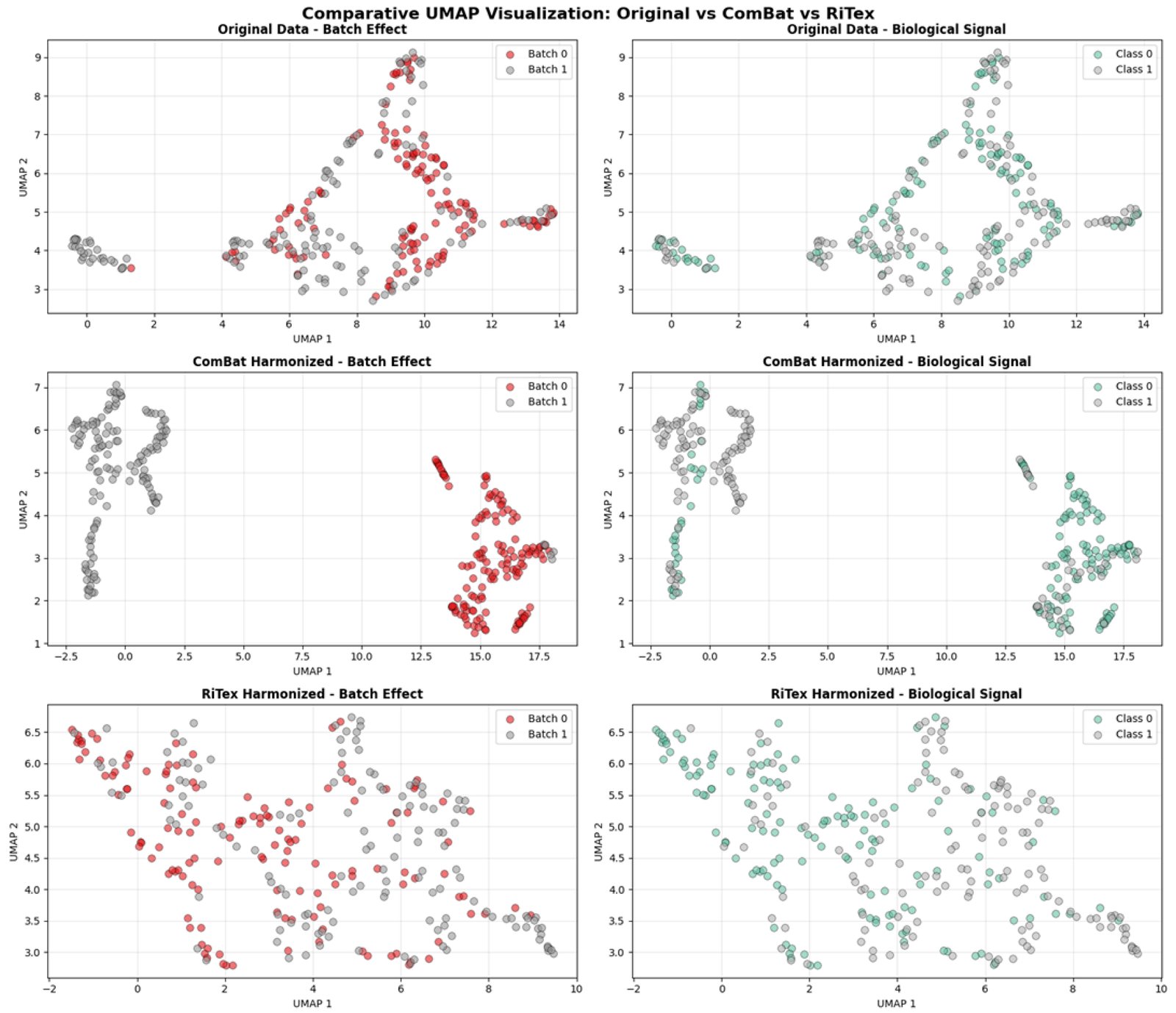
Dataset «Fusco2022»	
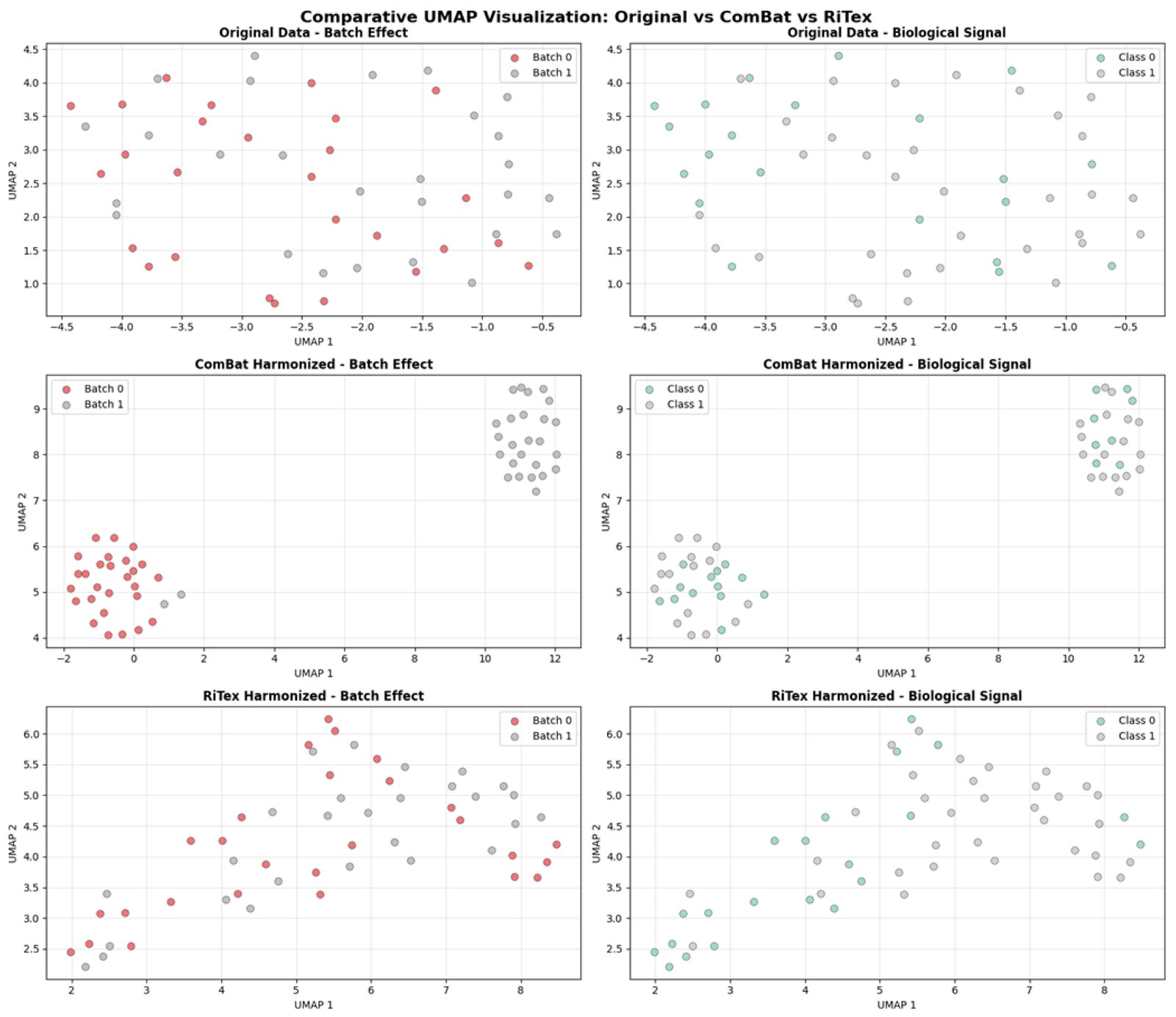
Dataset «Granata2021»	
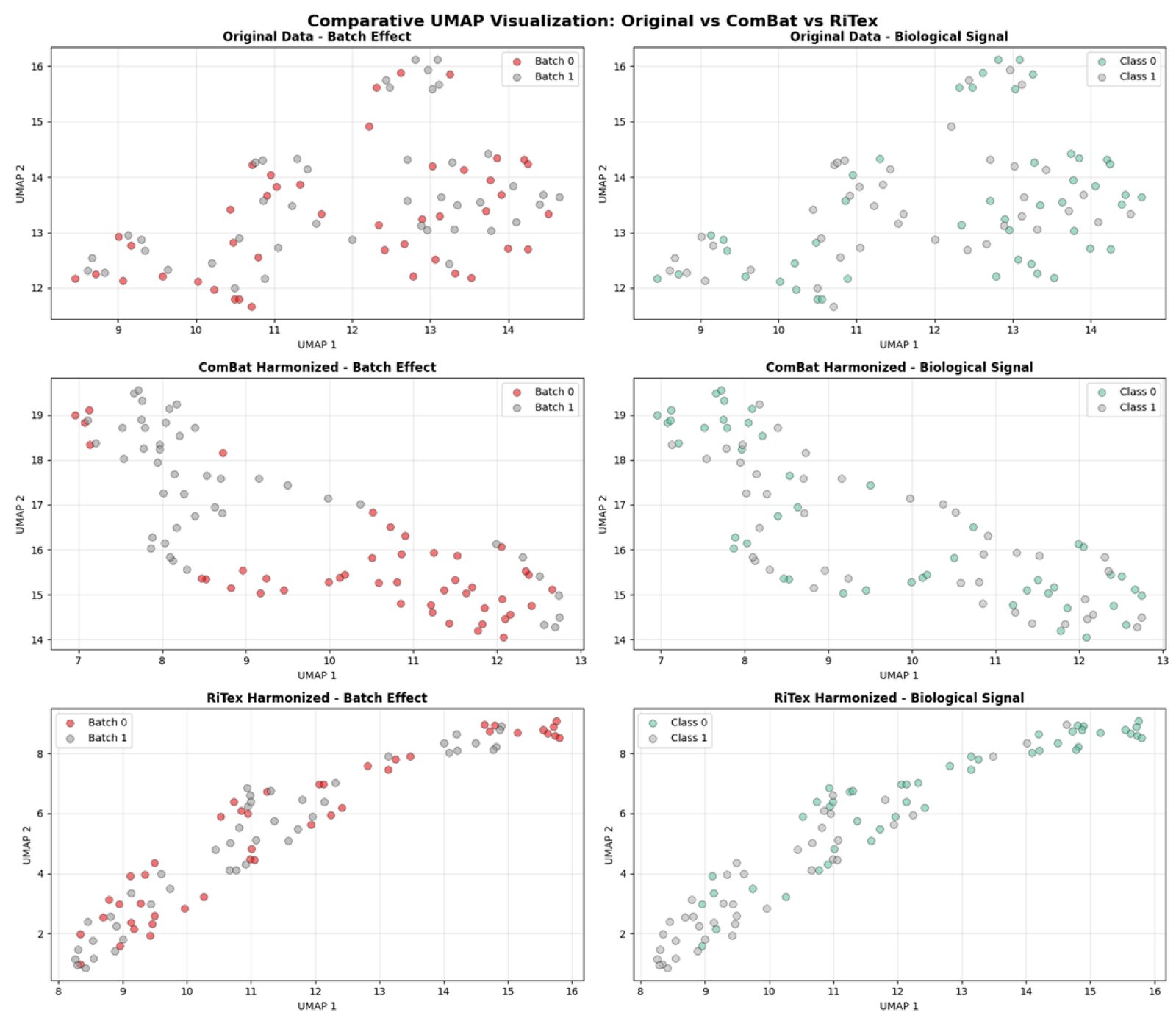
Dataset «Granata2024»	
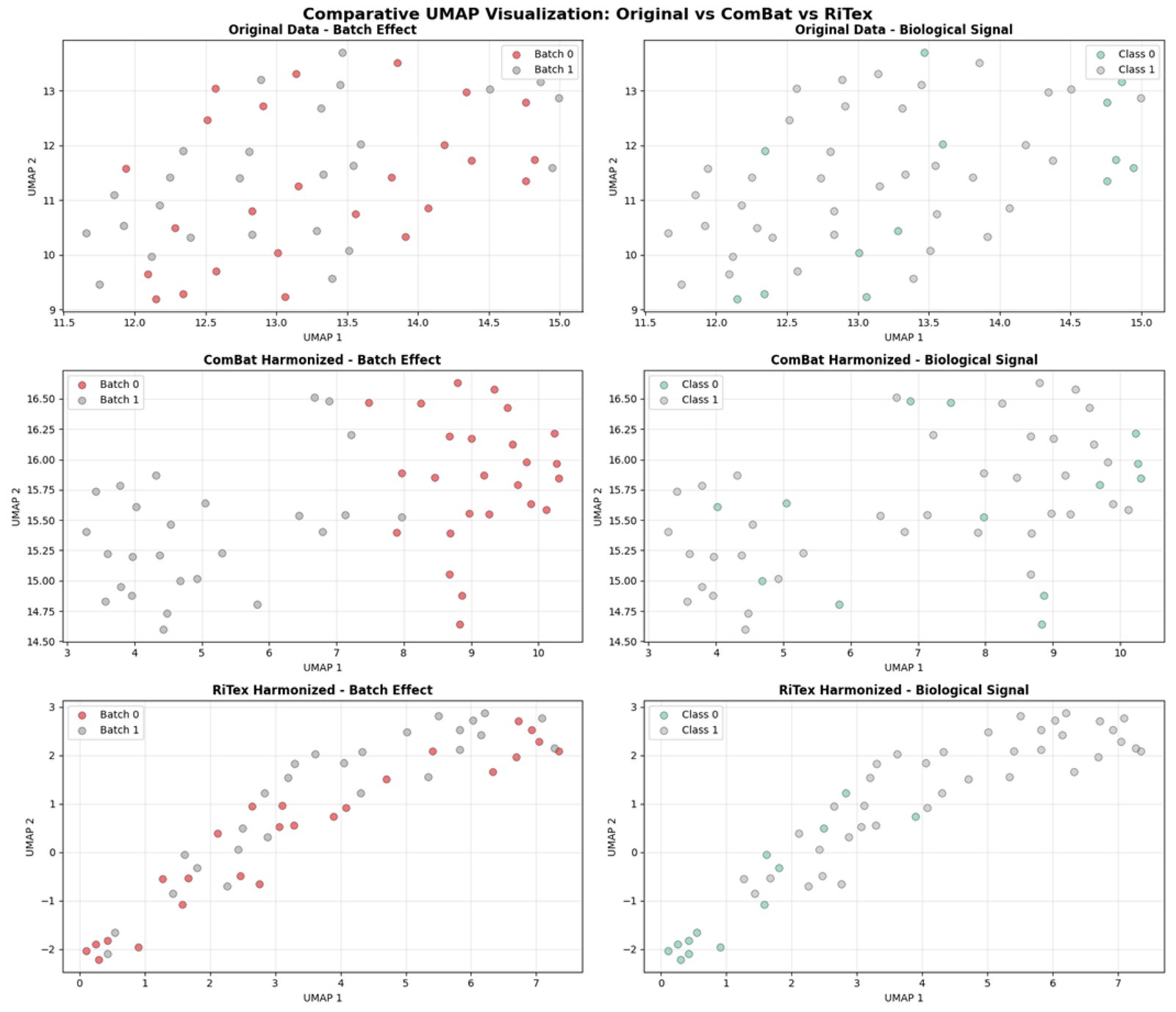
Dataset «HCC-TACE-Seg»	
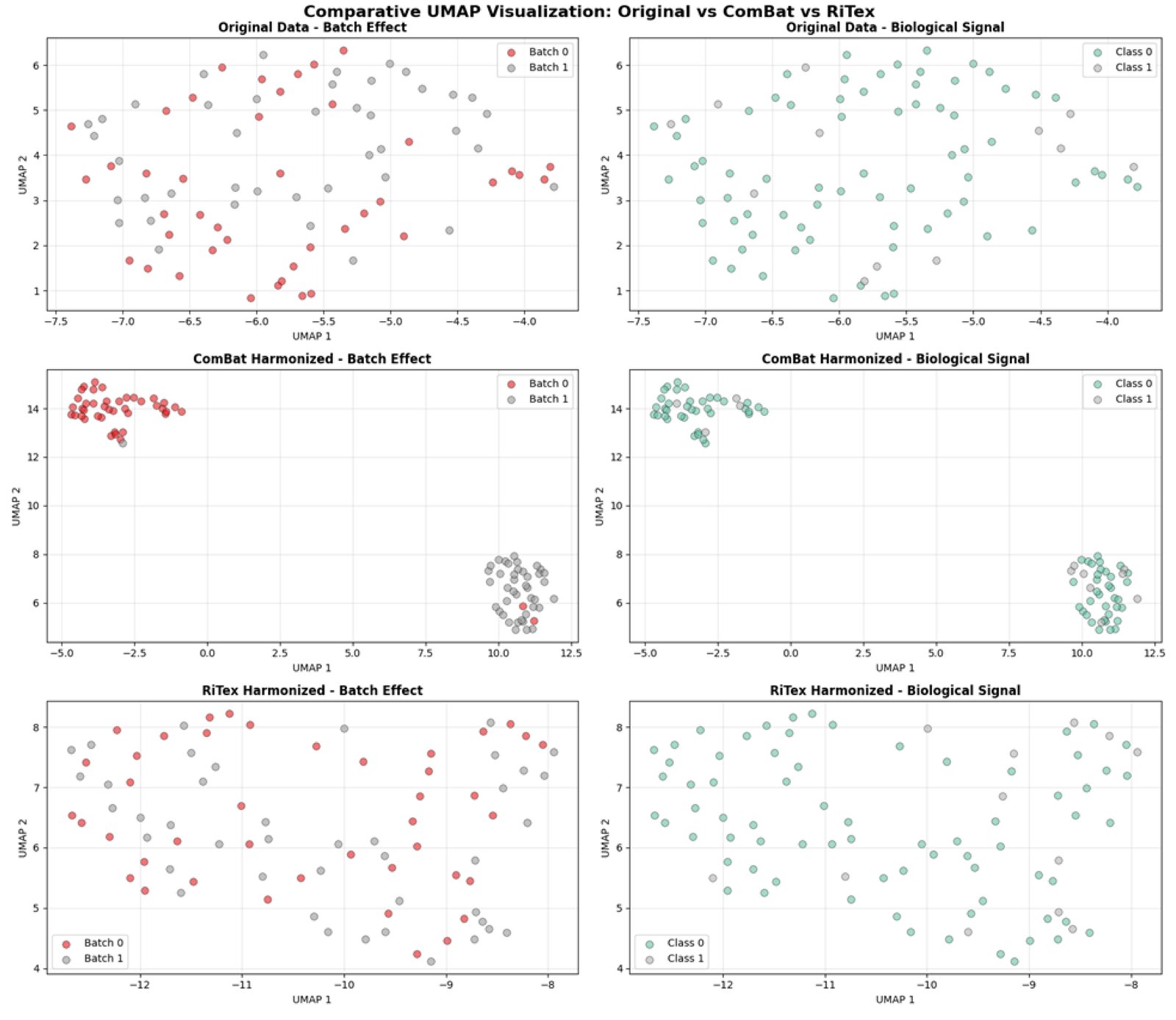
Dataset «HNSCC»	
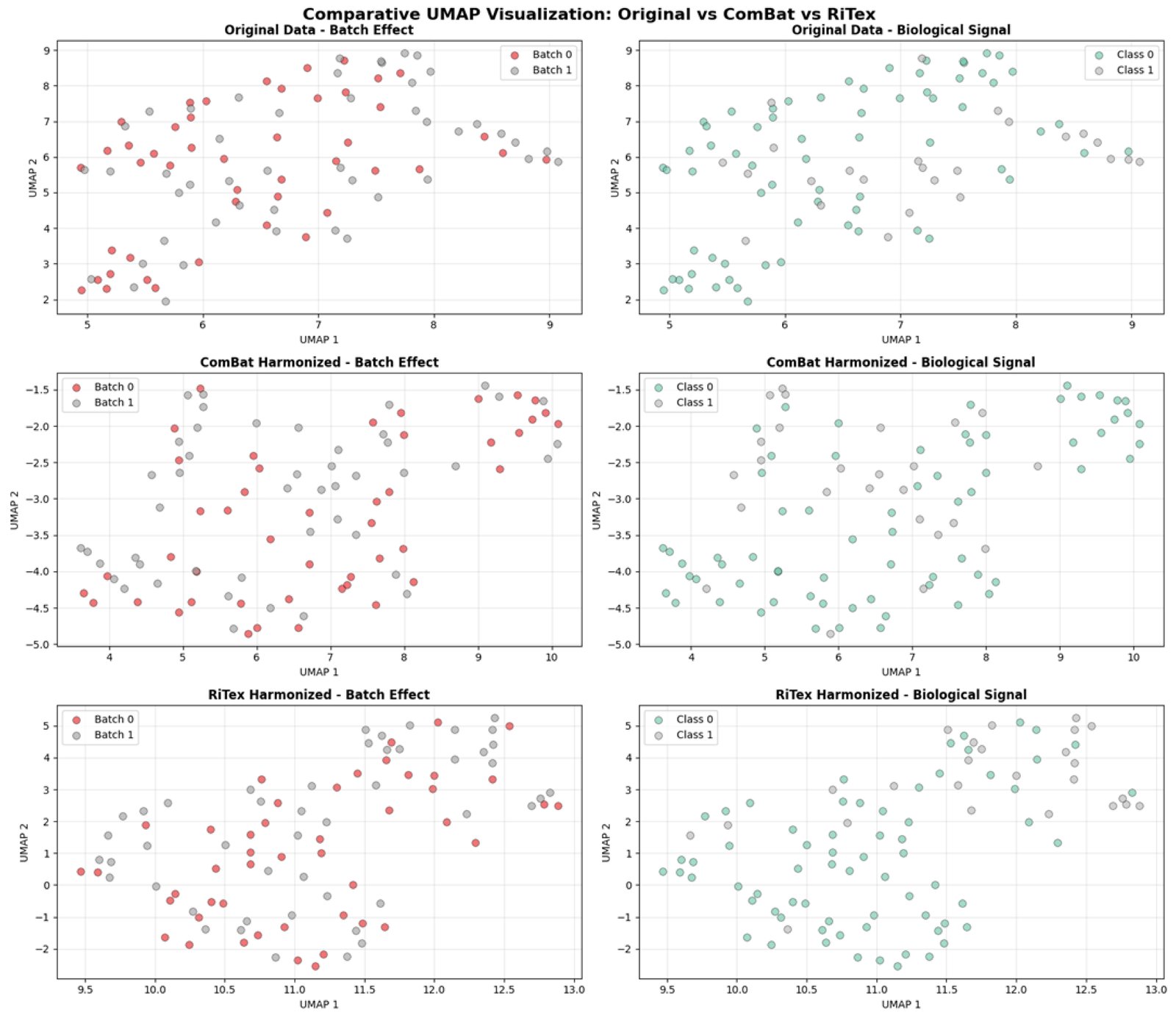
Dataset «Hosny2018A»	
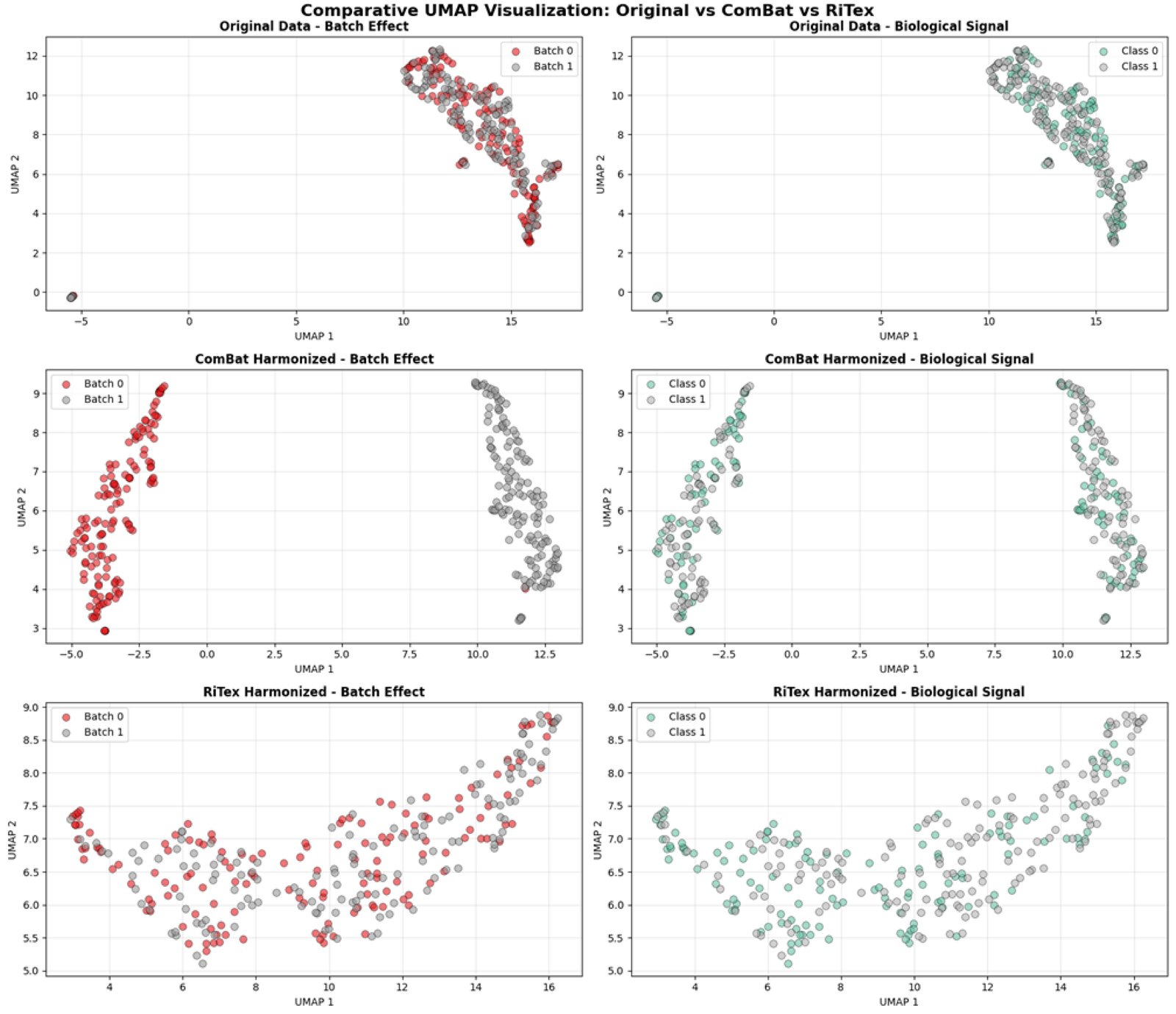
Dataset «Hosny2018B»	
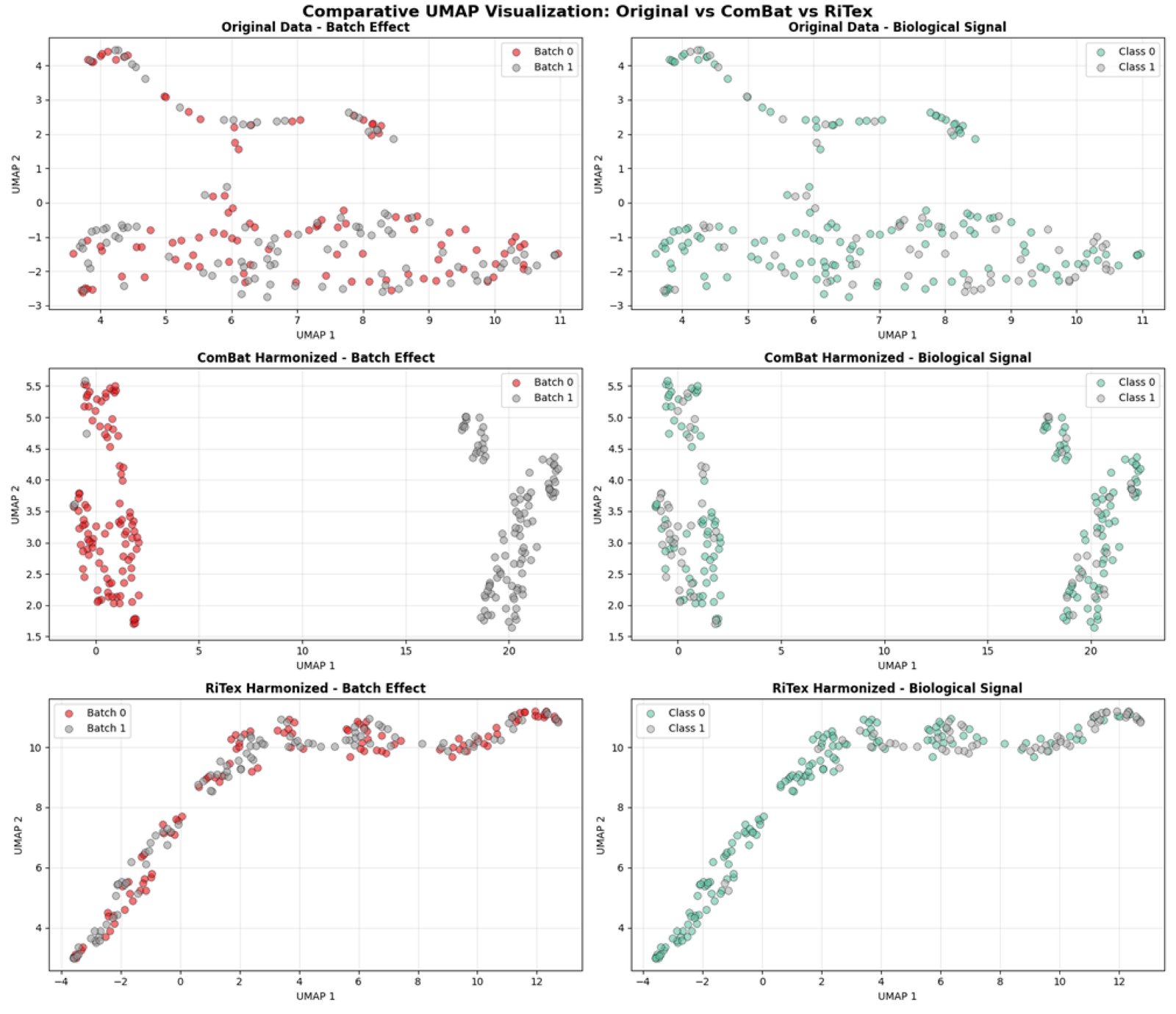
Dataset «Hosny2018C»	
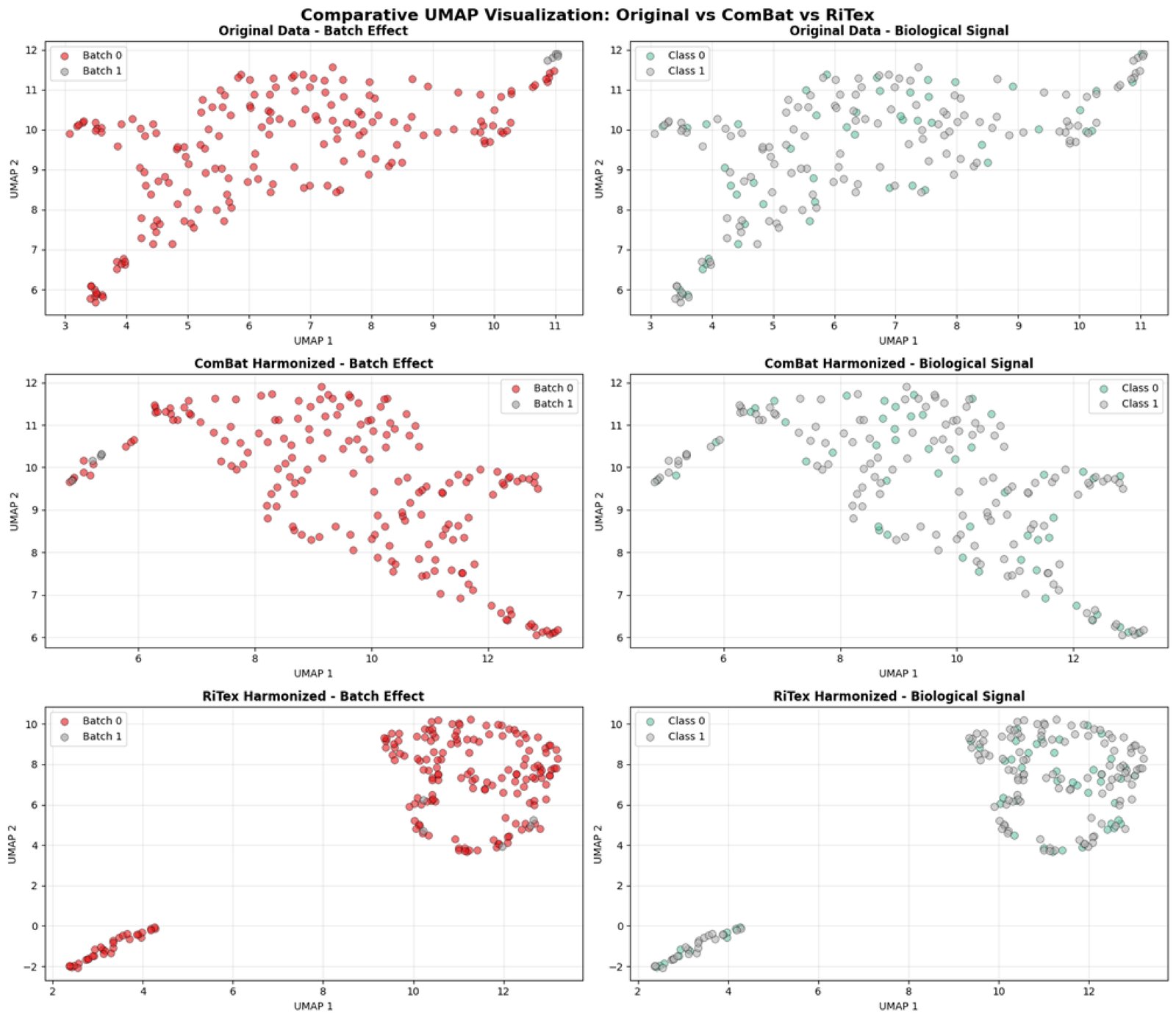
Dataset «Huang2023»	
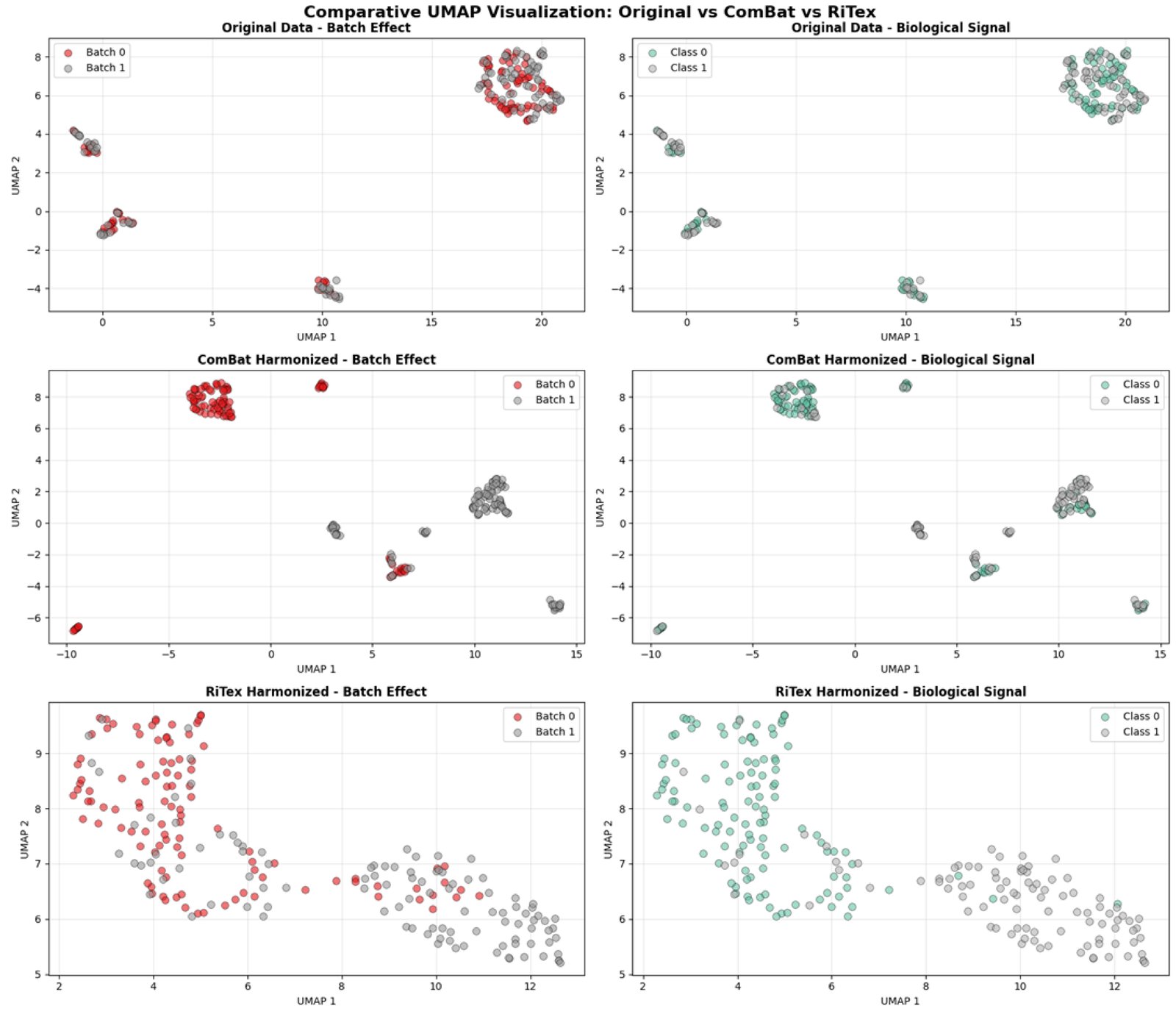
Dataset «Hunter2023»	
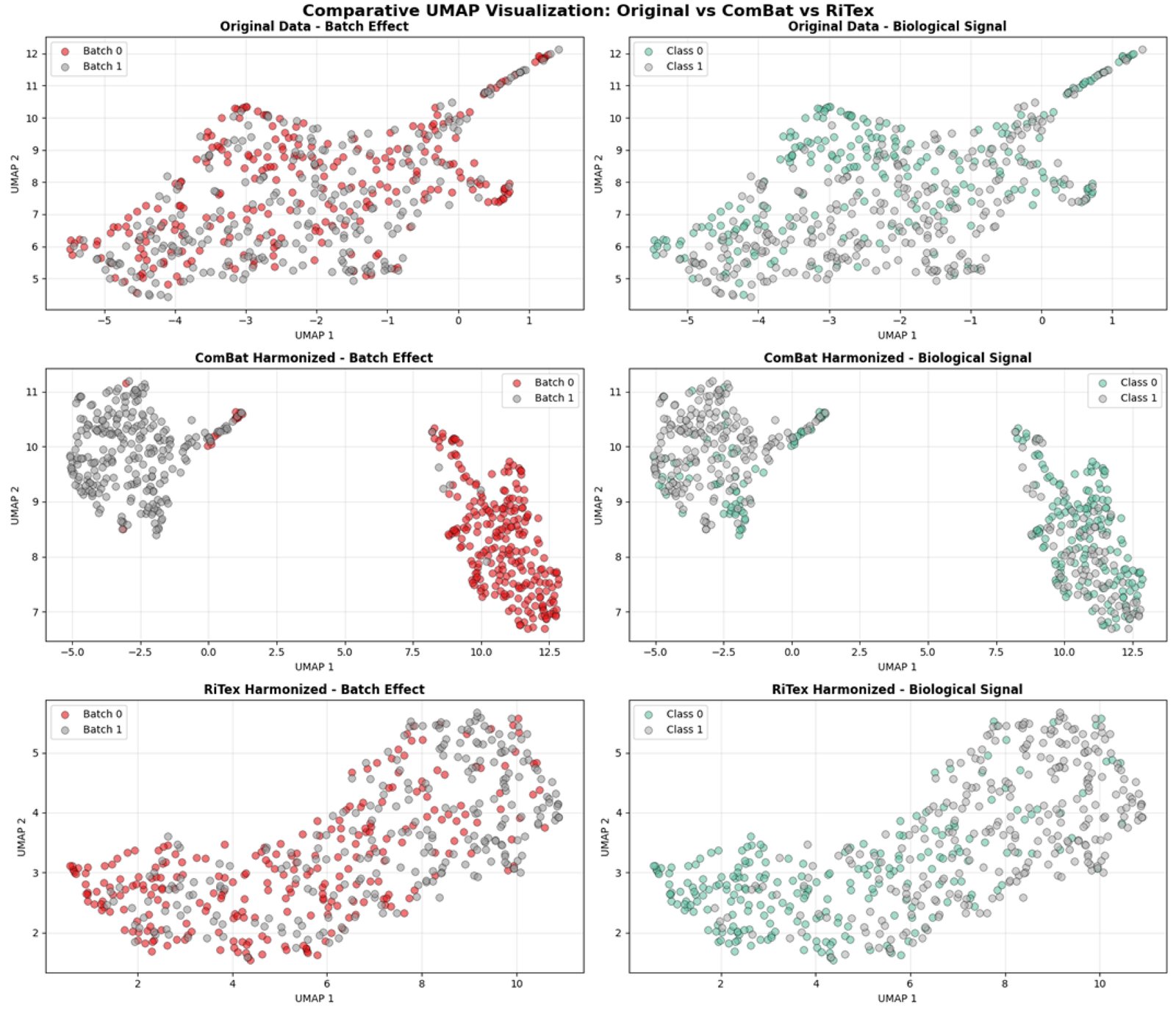
Dataset «ISPY1»	
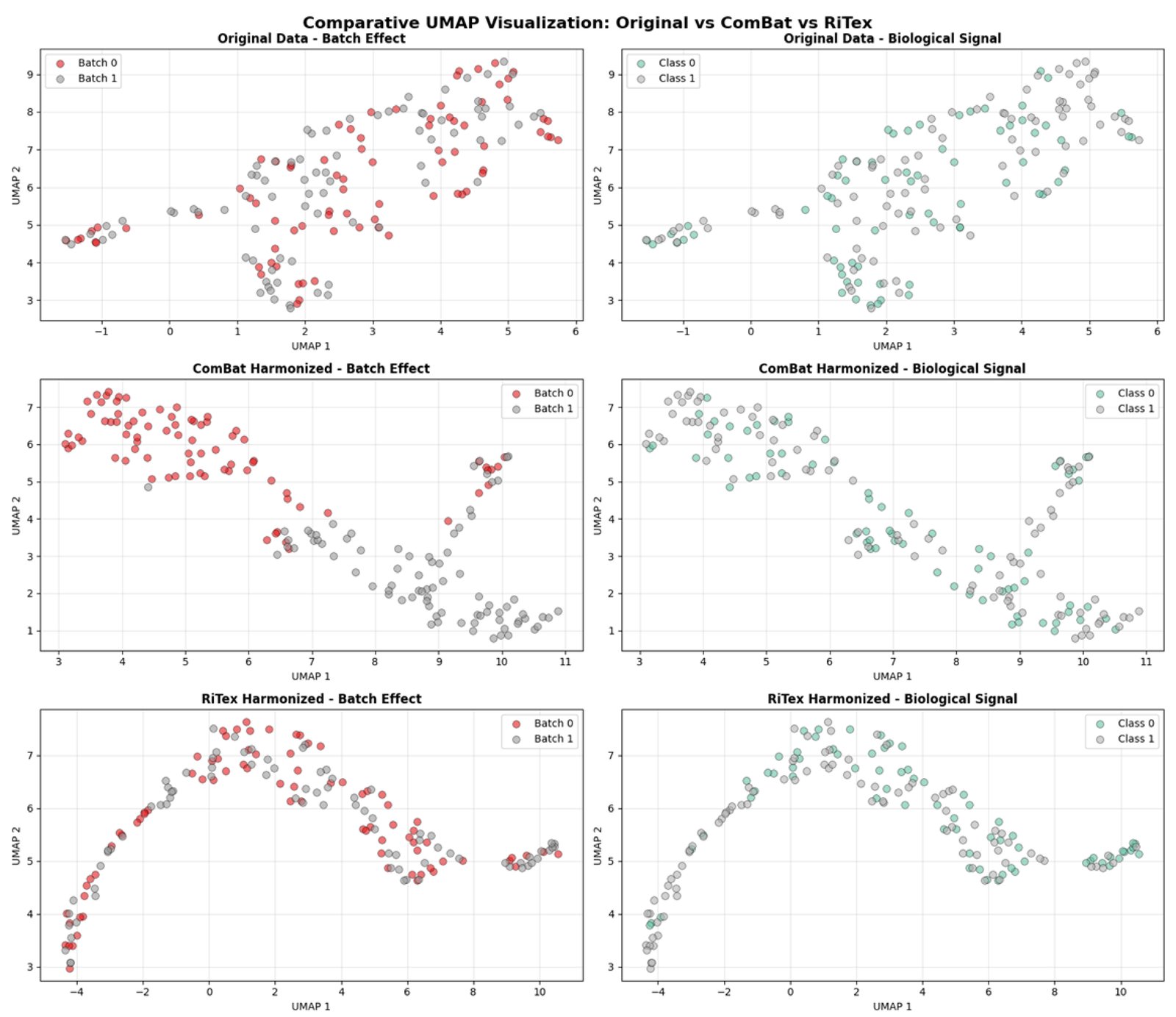
Dataset «Keek2020»	
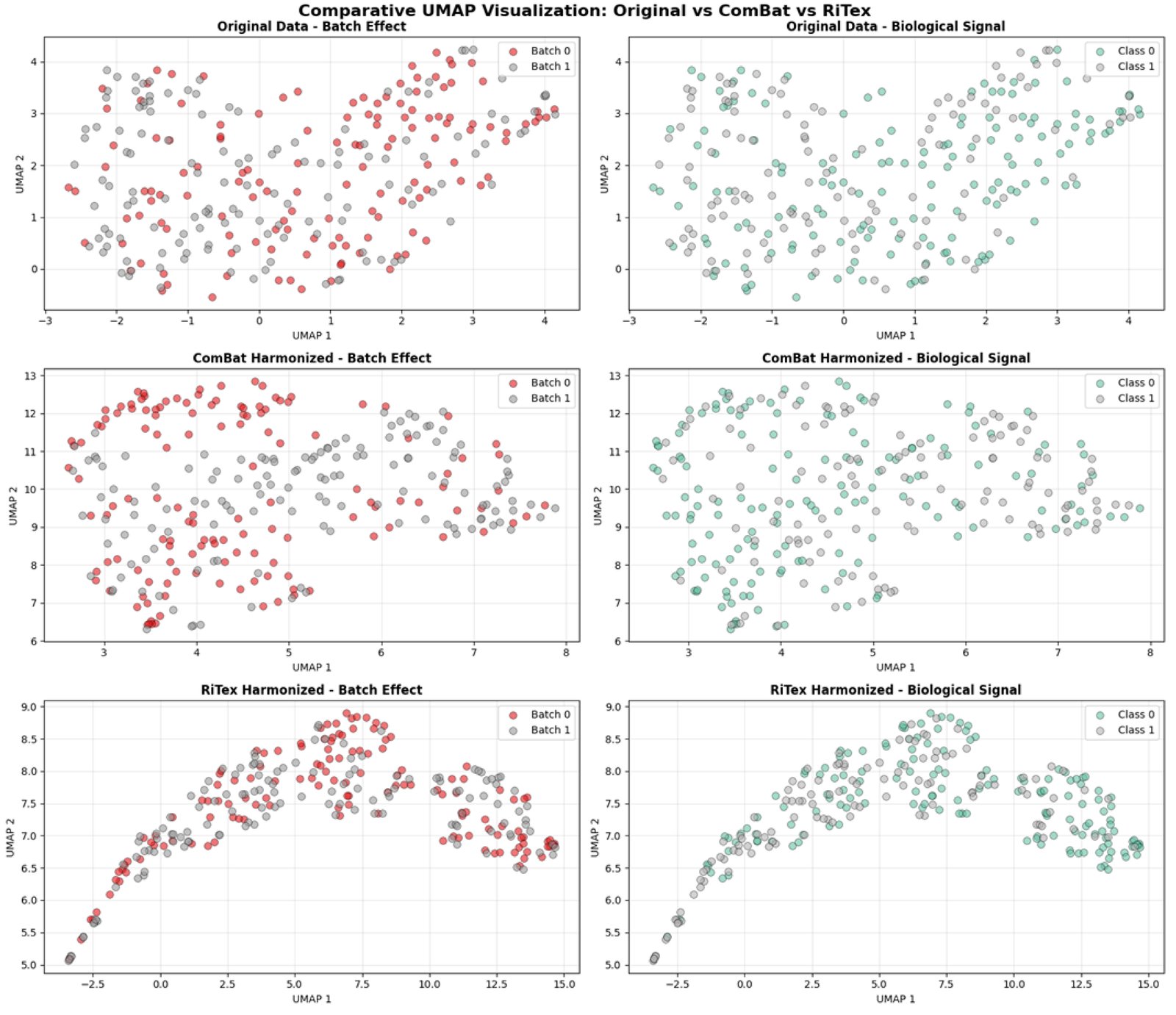
Dataset «LGG-1p19qDeletion»	
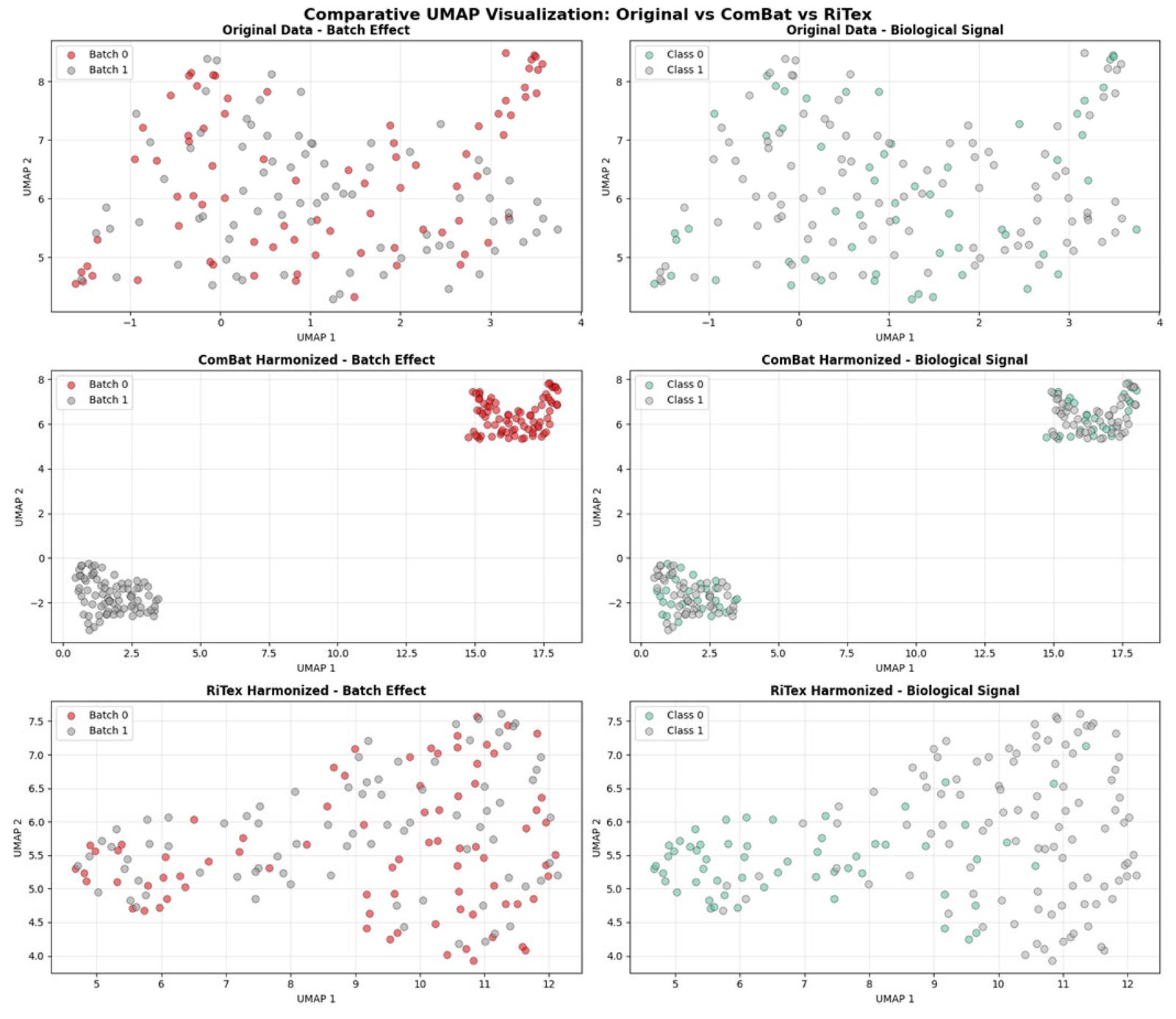
Dataset «LNDb»	
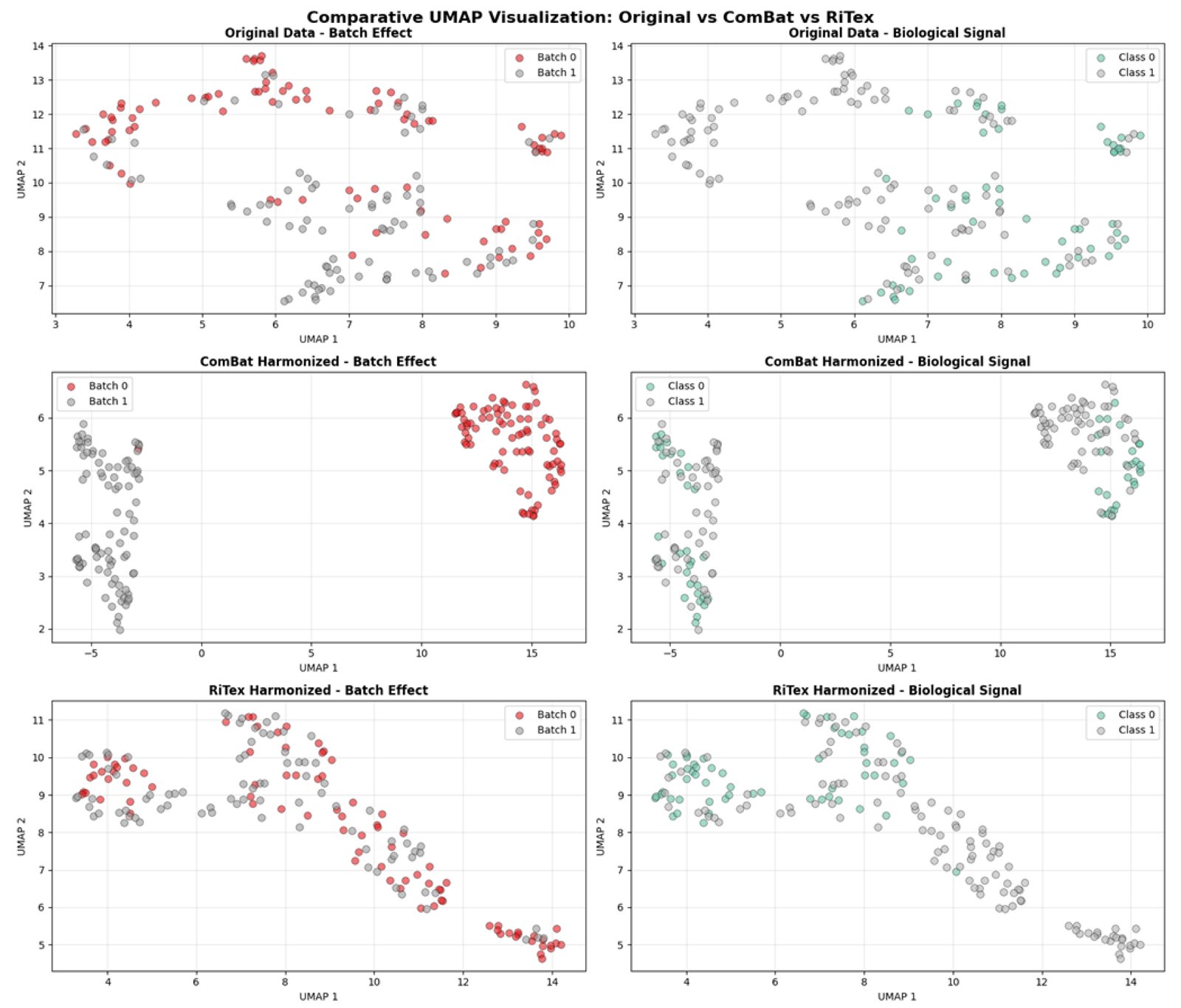
Dataset «Li2020»	
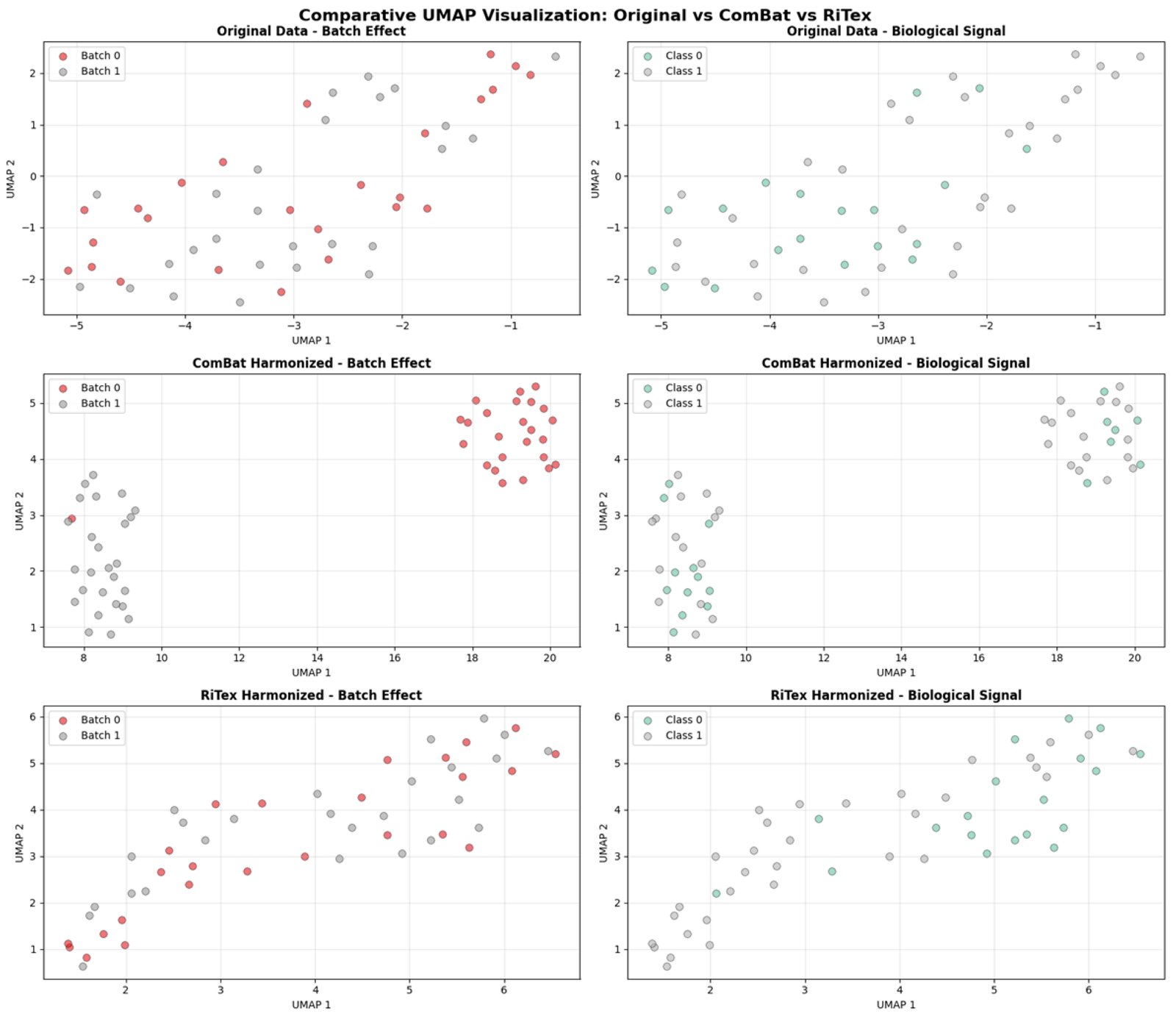
Dataset «Lu2019»	
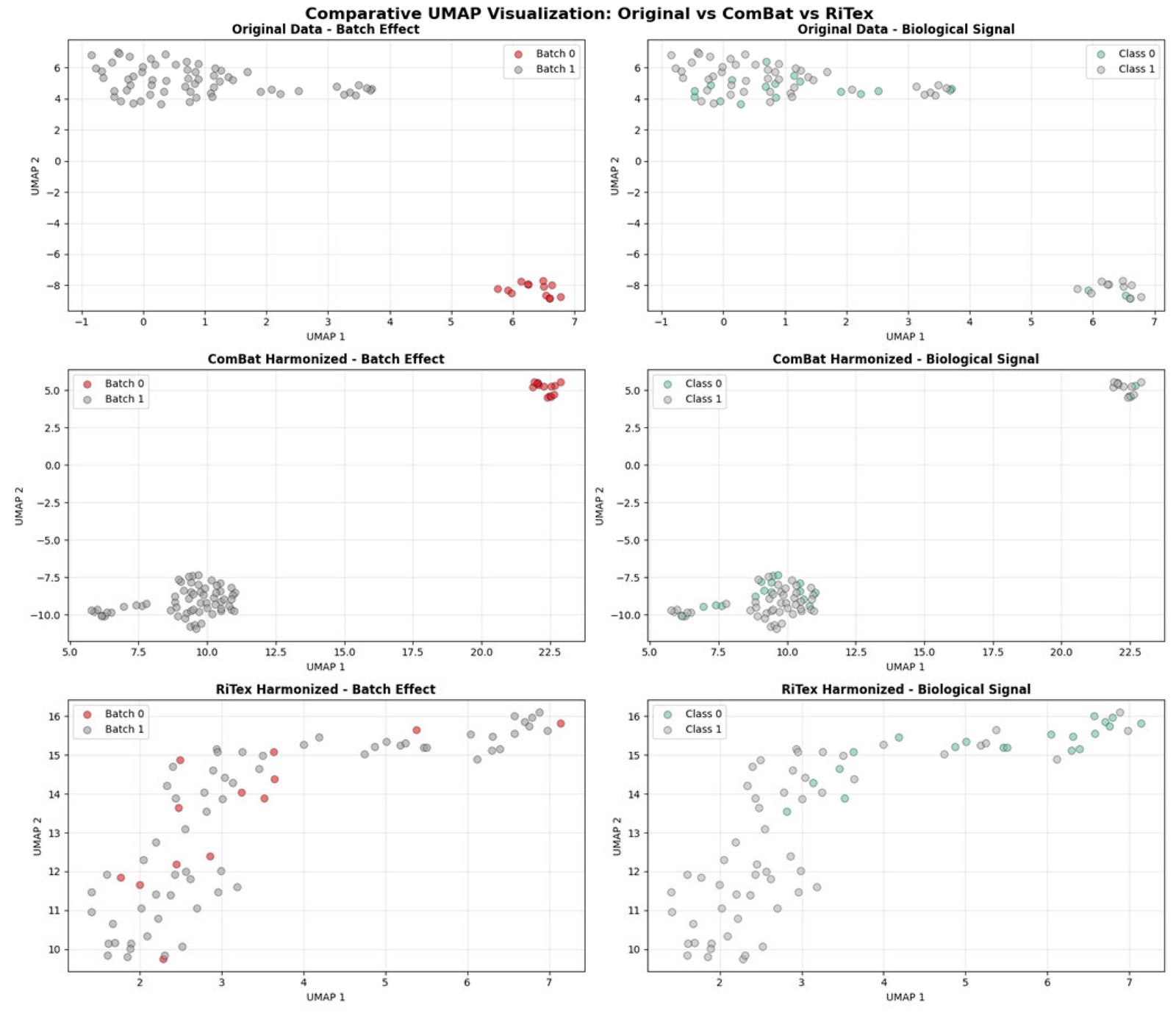
Dataset «Meningioma-SEG-CLASS»	
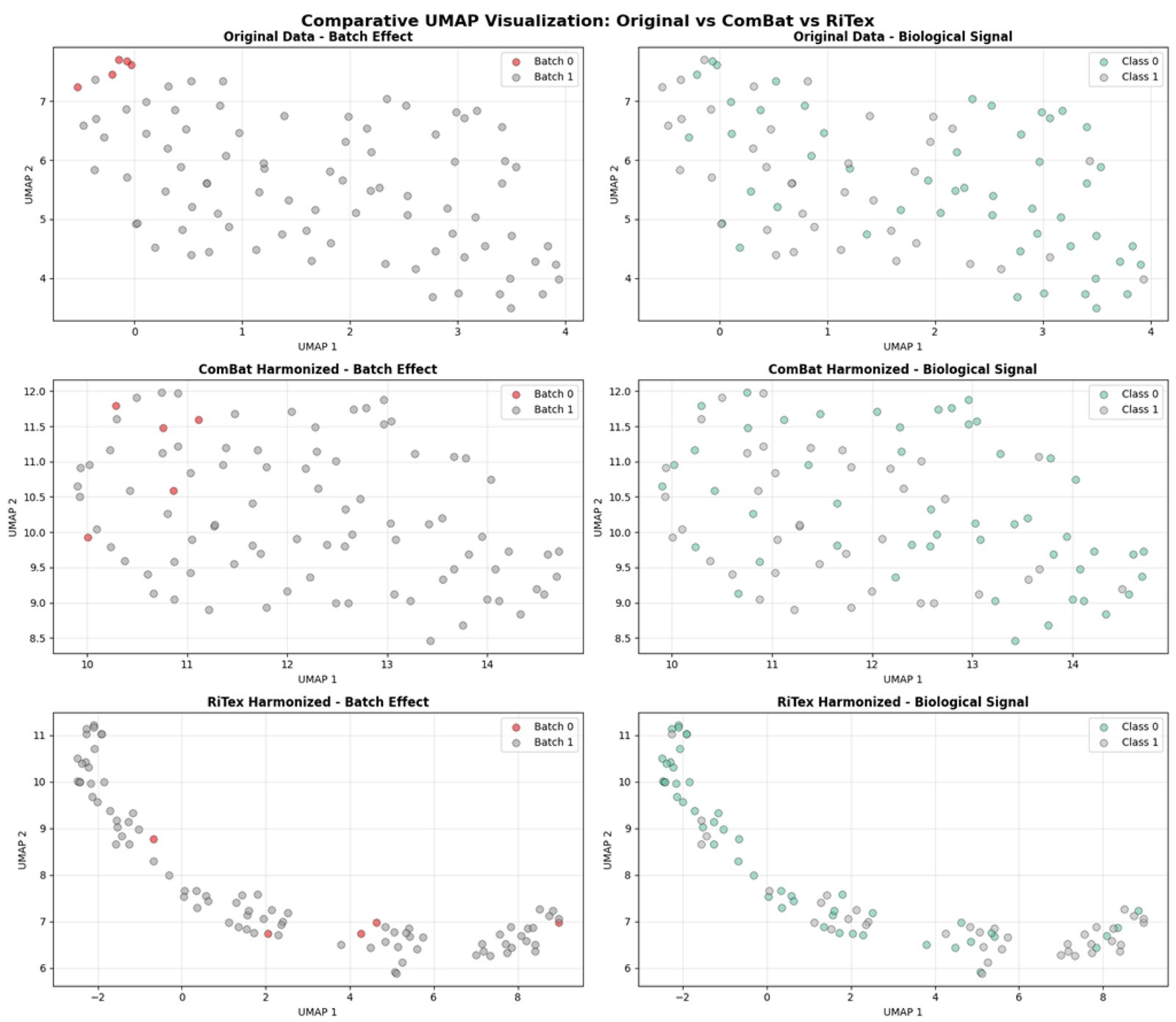
Dataset «NSCLC-Radiogenomics»	
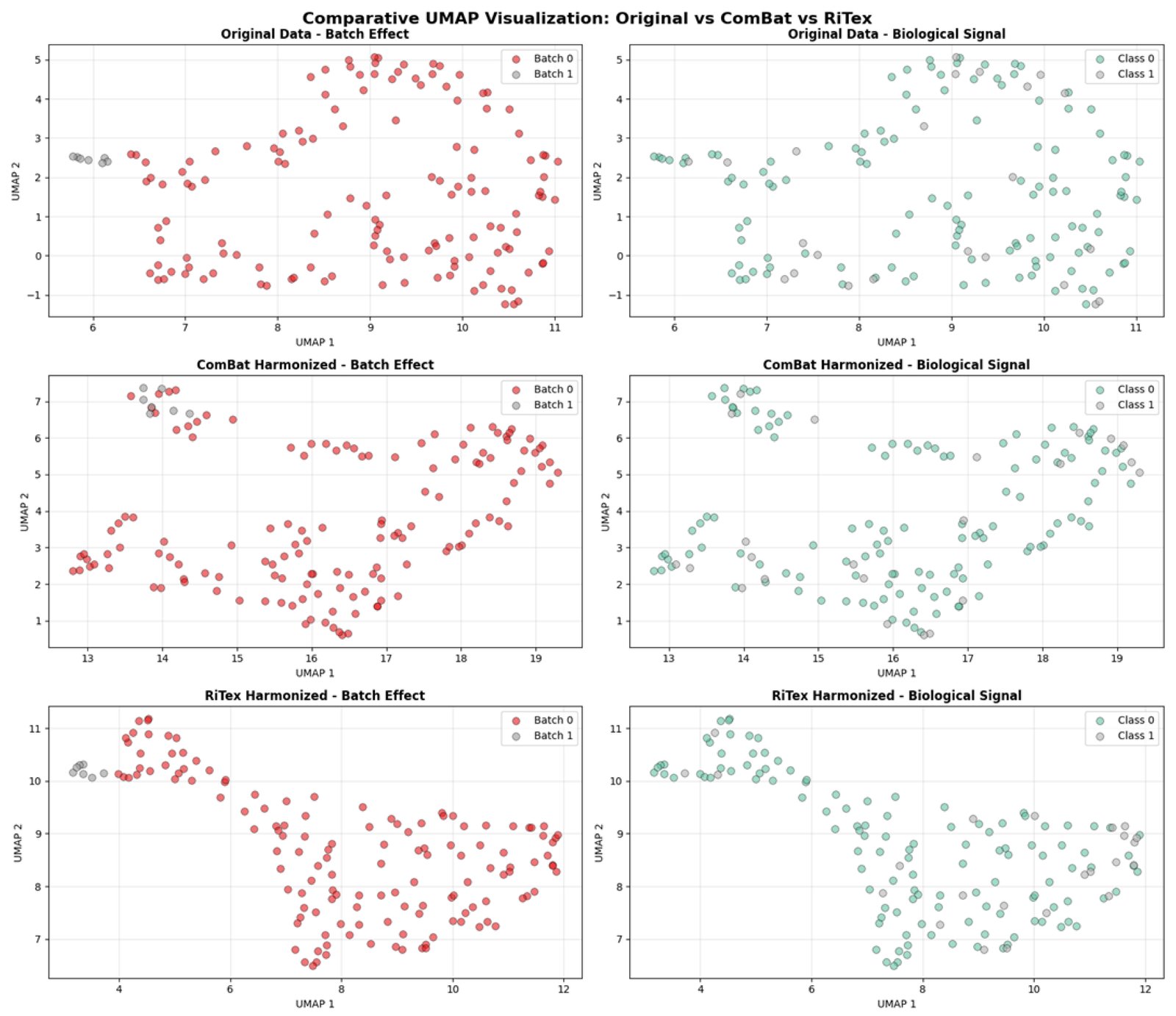
Dataset «OcanaTienda2023»	
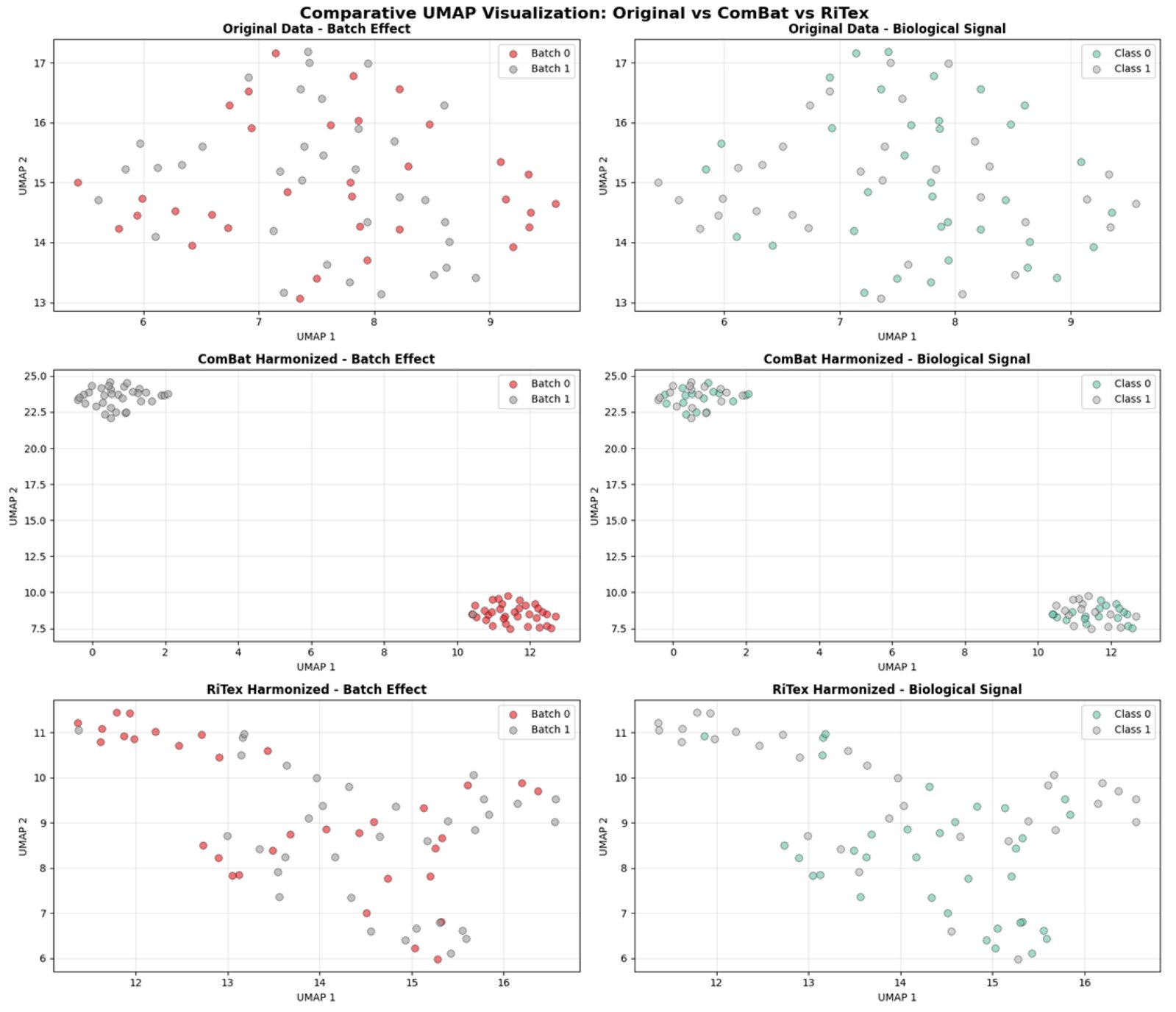
Dataset «PI-CAI»	
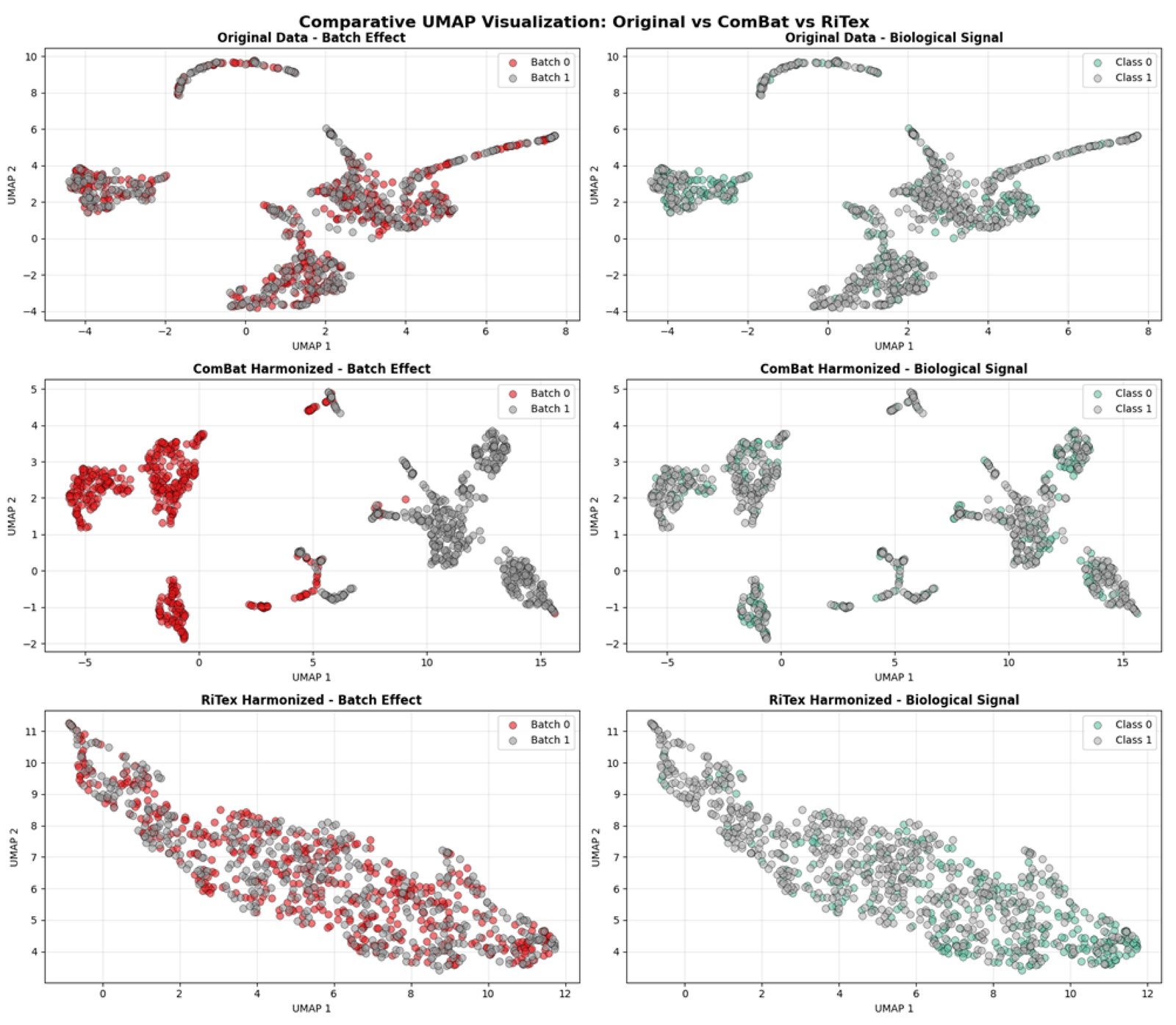
Dataset «Petrillo2023»	
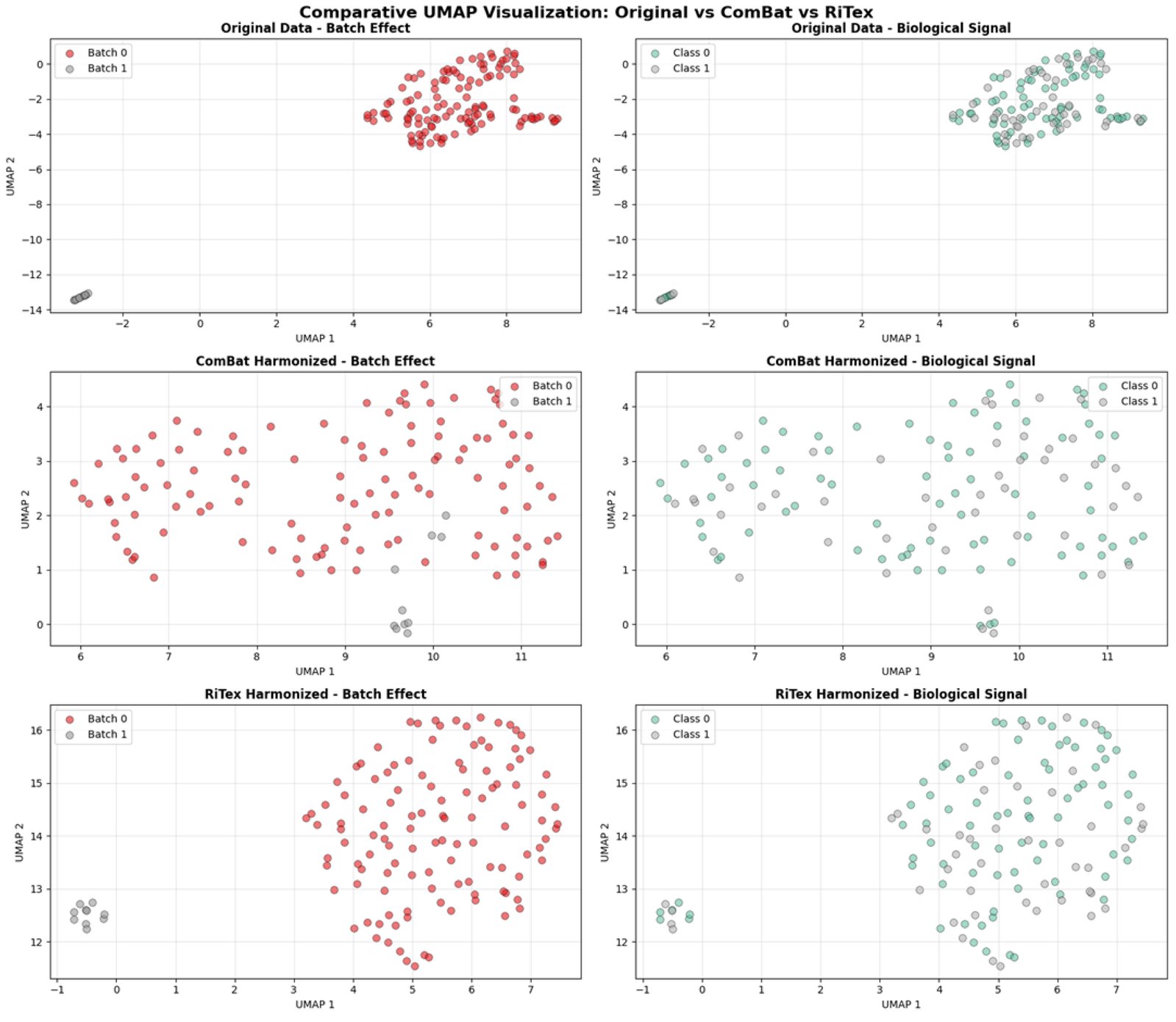
Dataset «Prostate-MRI-US-Biopsy»	
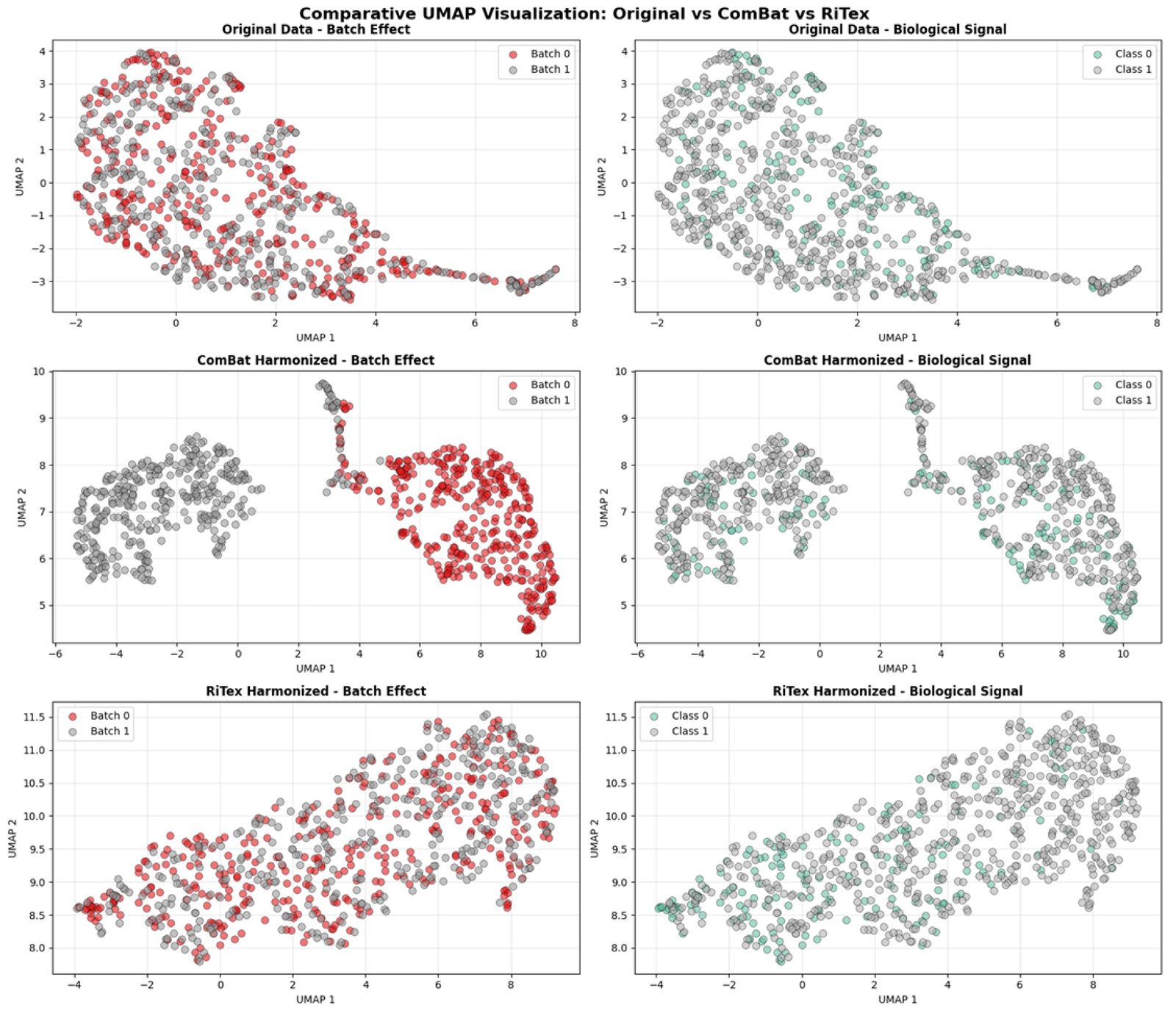
Dataset «QIN-HEADNECK»	
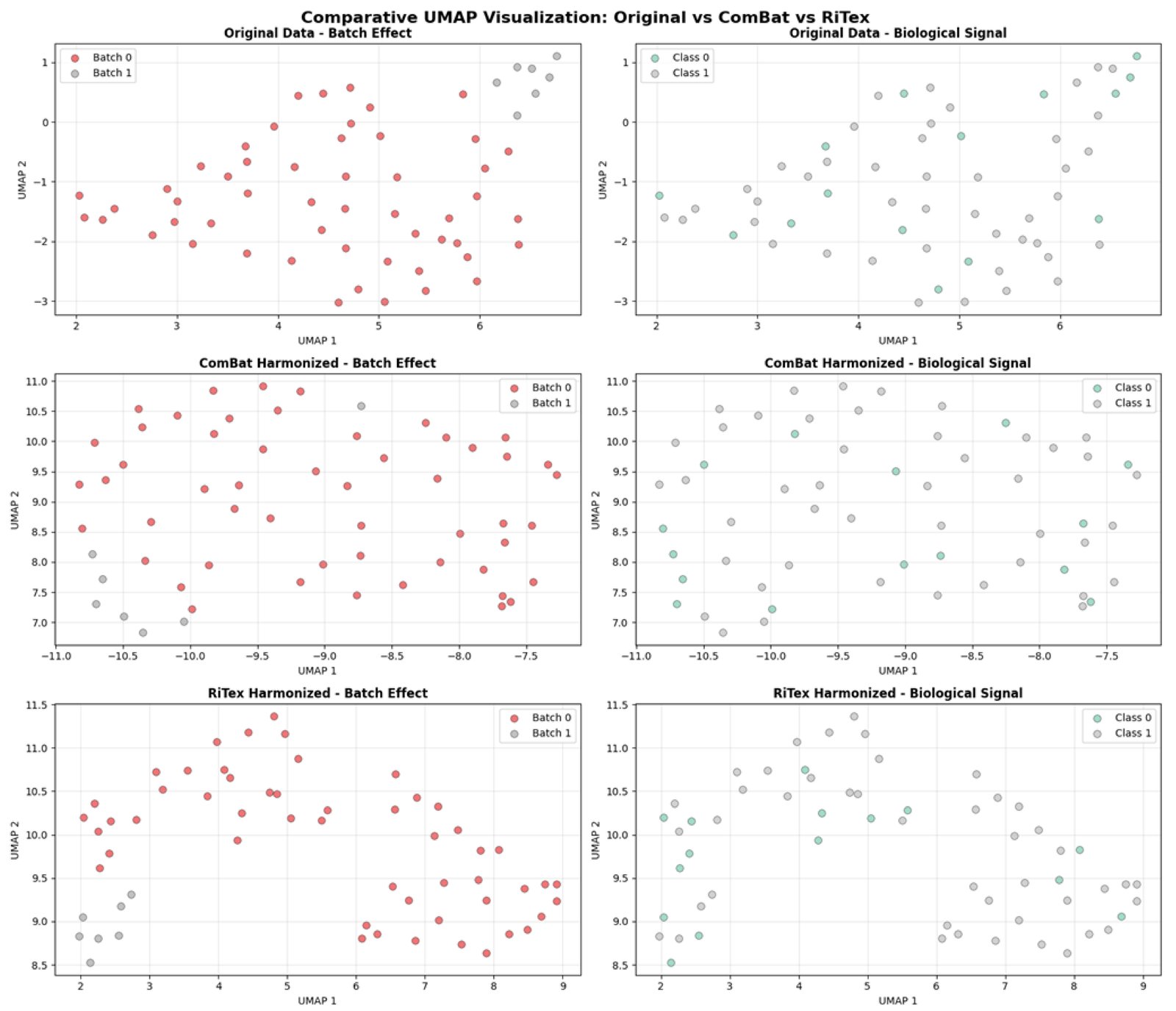
Dataset «Ramella2018»	
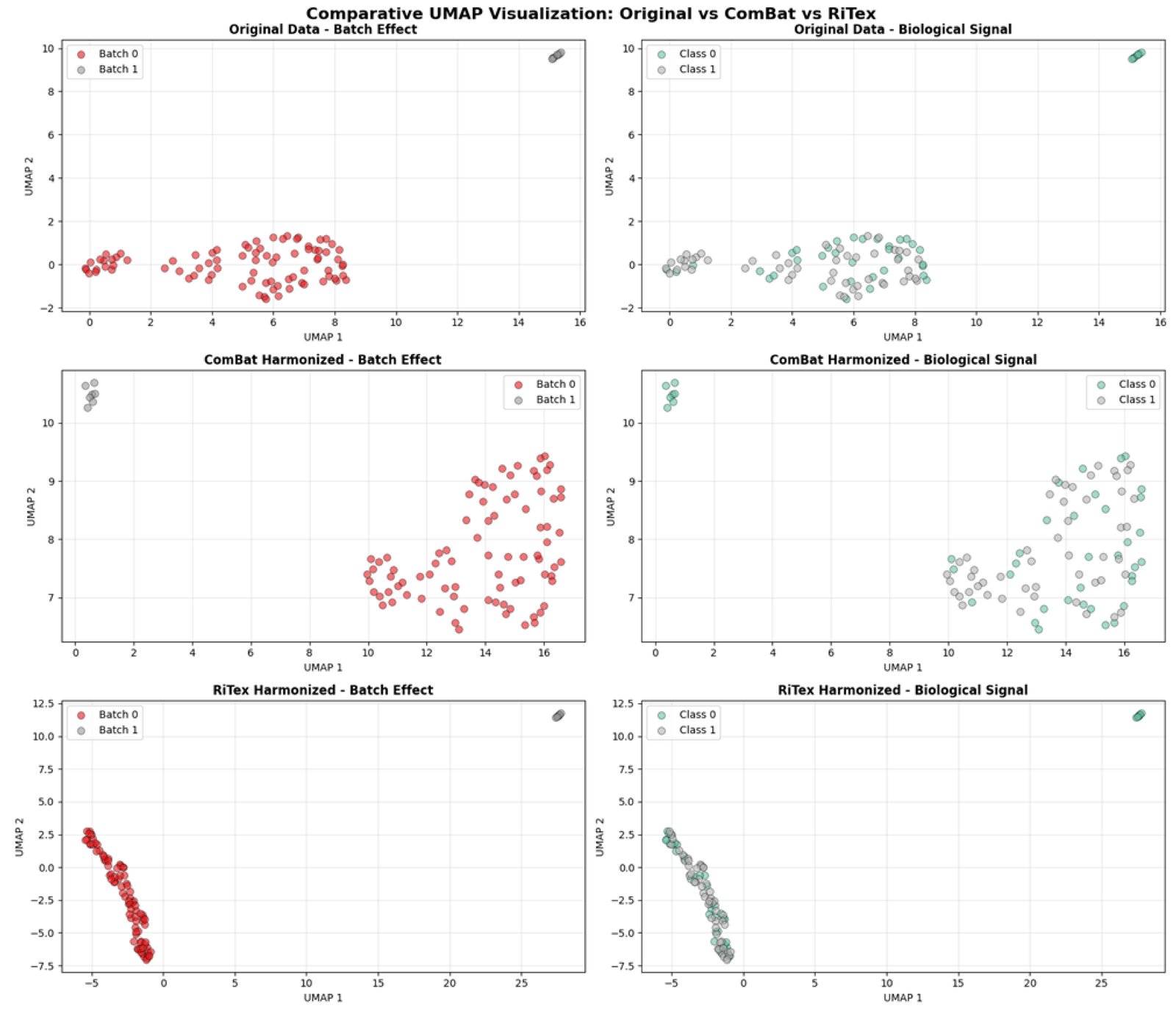
Dataset «Sasaki2019»	
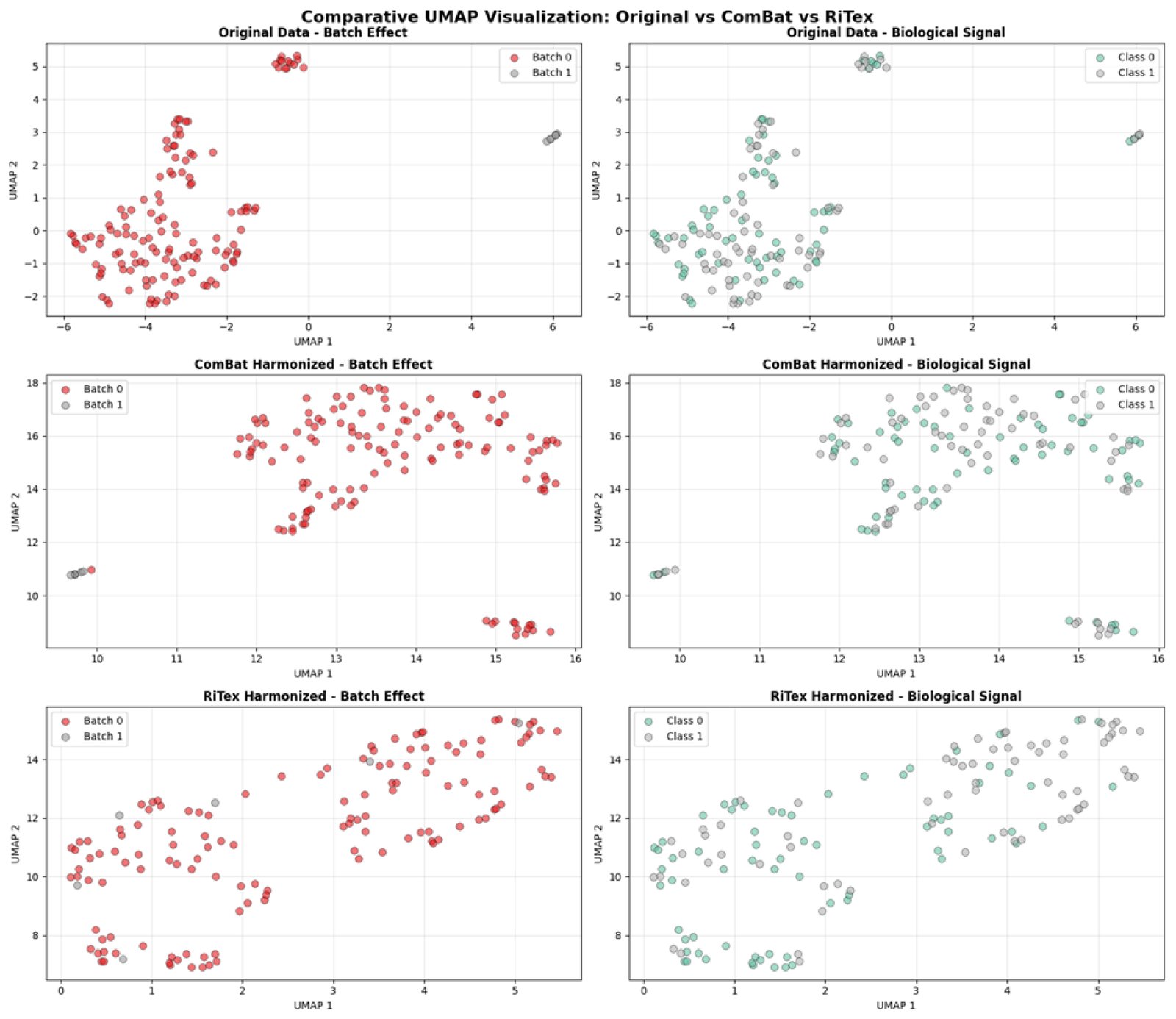
Dataset «UCSF-PDGM»	
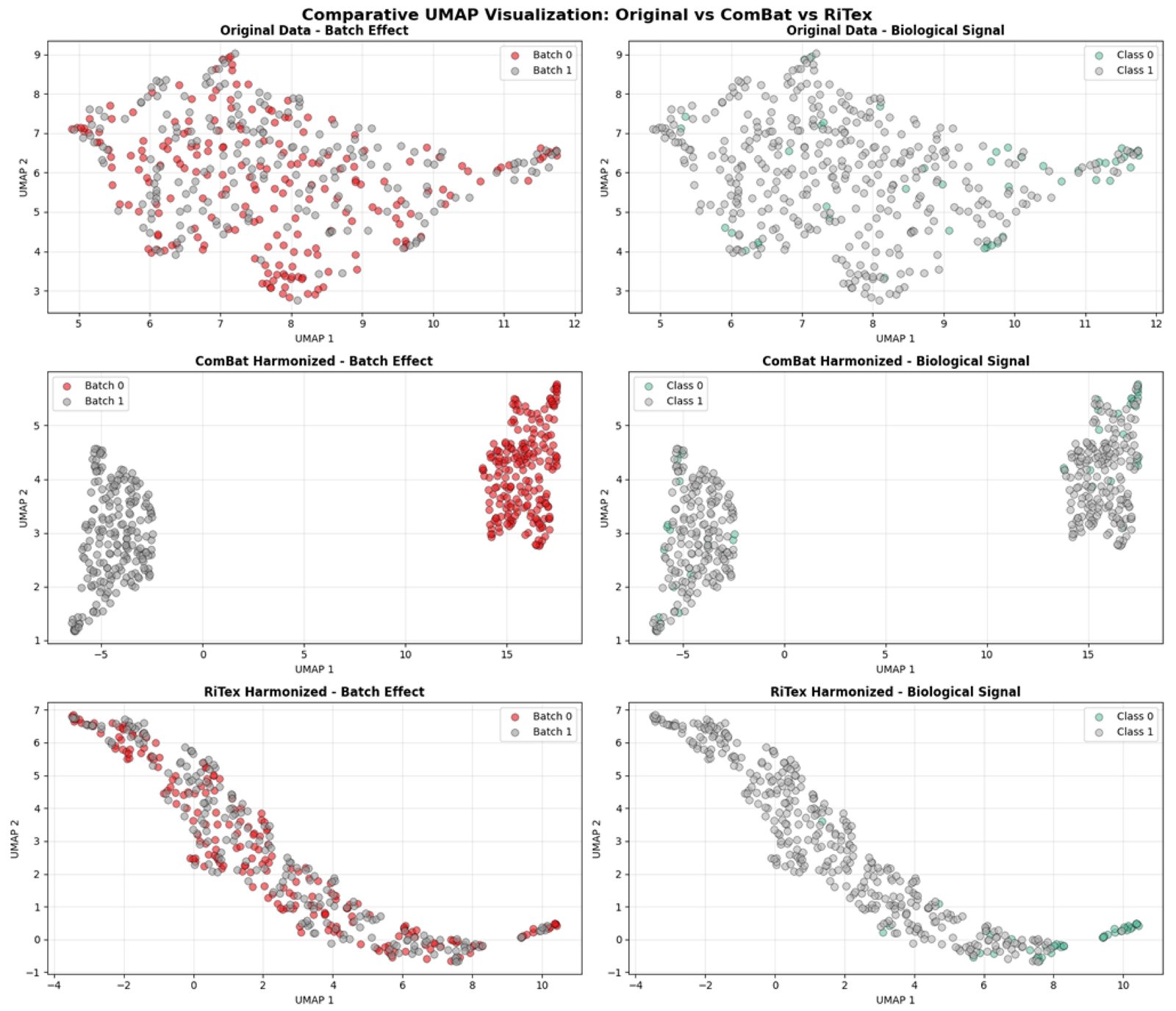
Dataset «UPENN-GBM»	
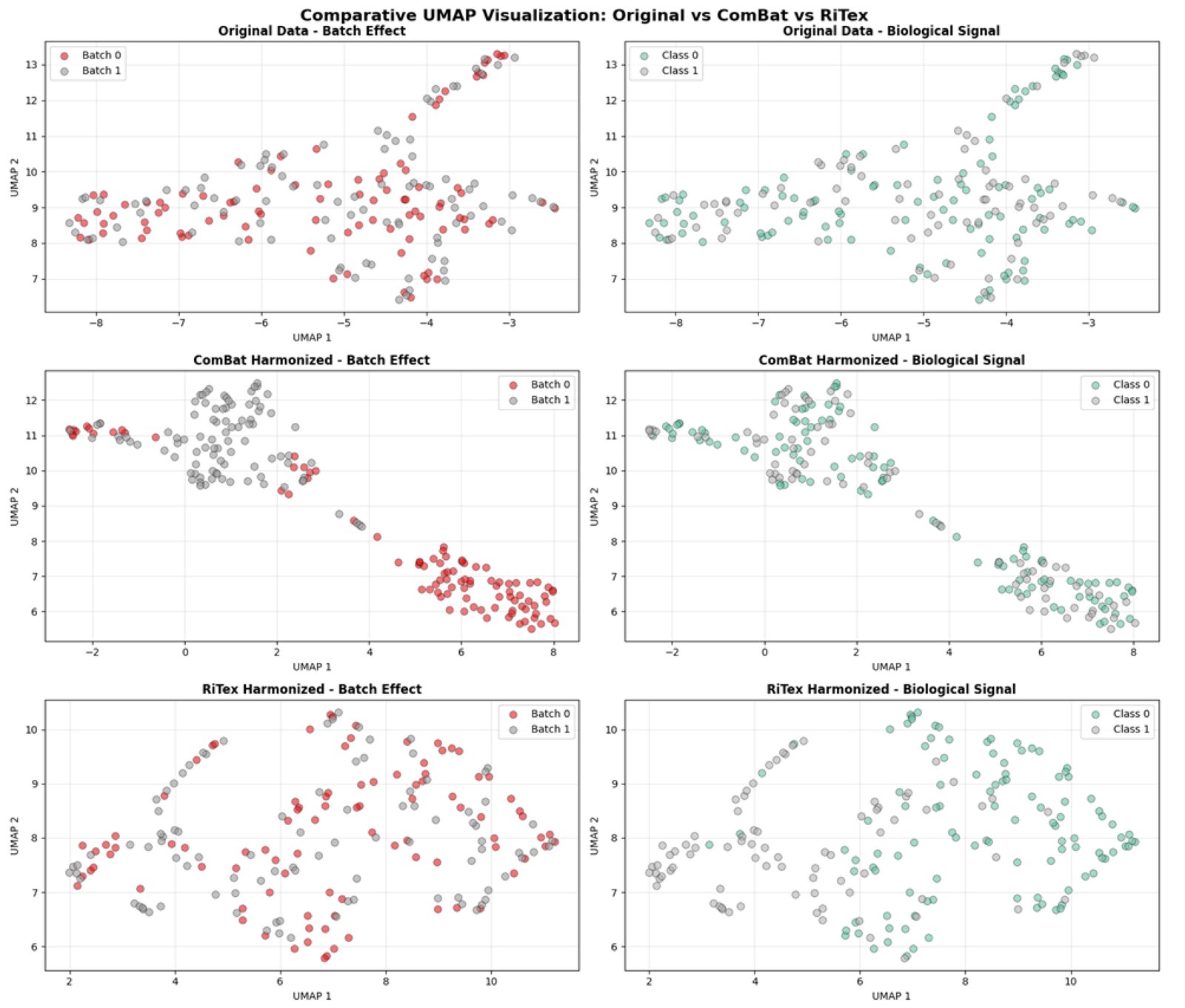
Dataset «Veeraraghavan2020»	
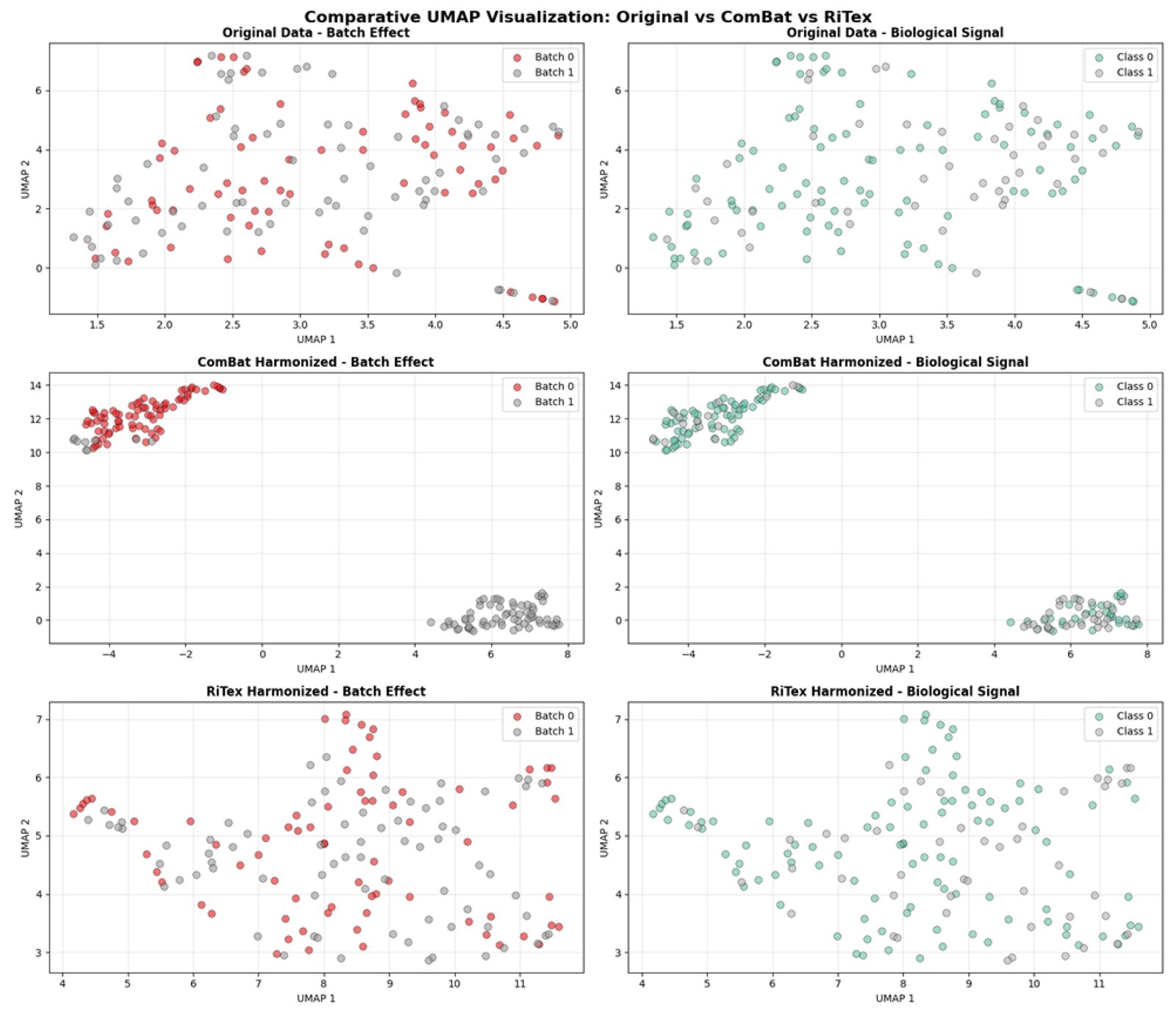
Dataset «WORC-CRLM»	
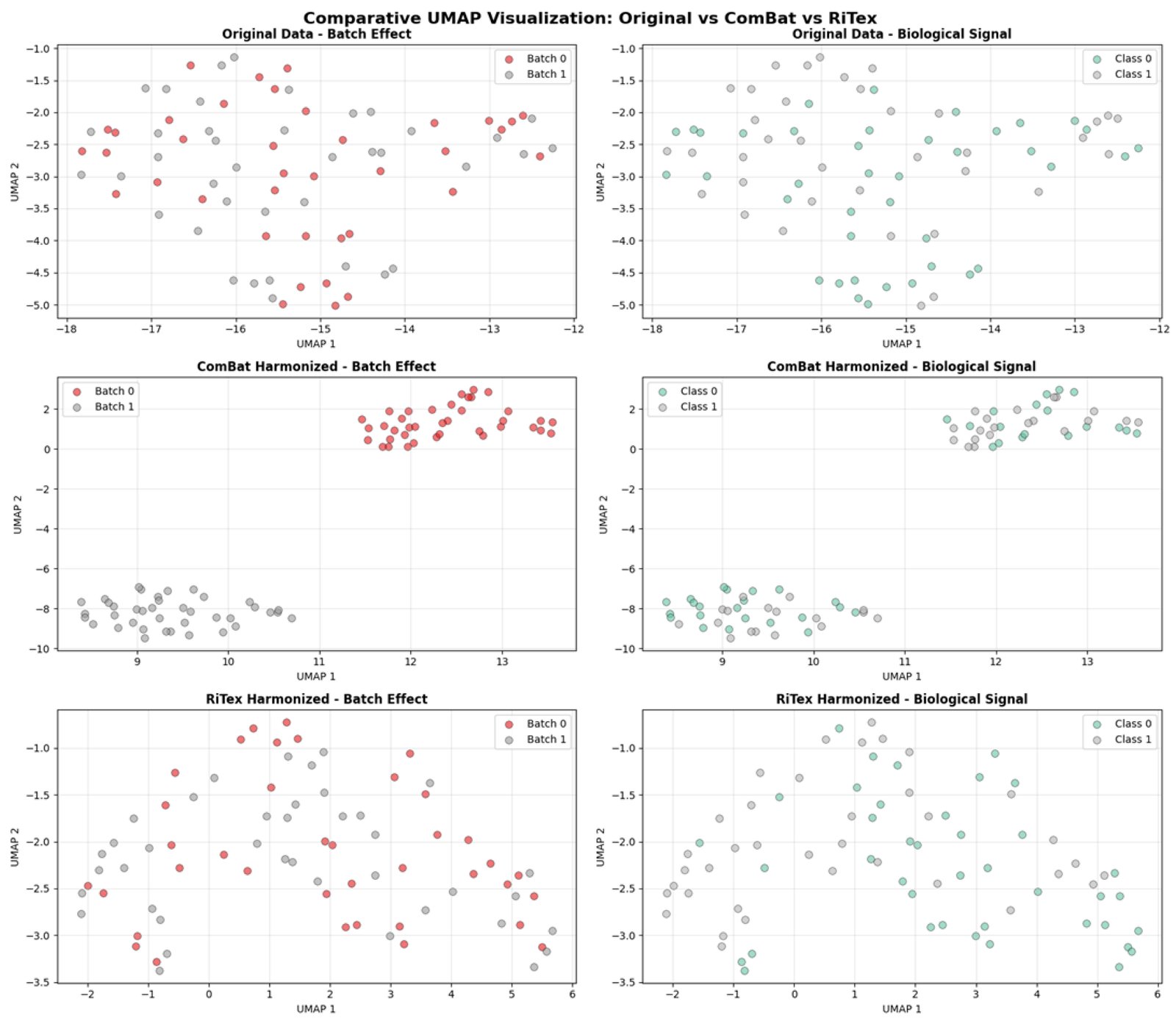
Dataset «WORC-Desmoid»	
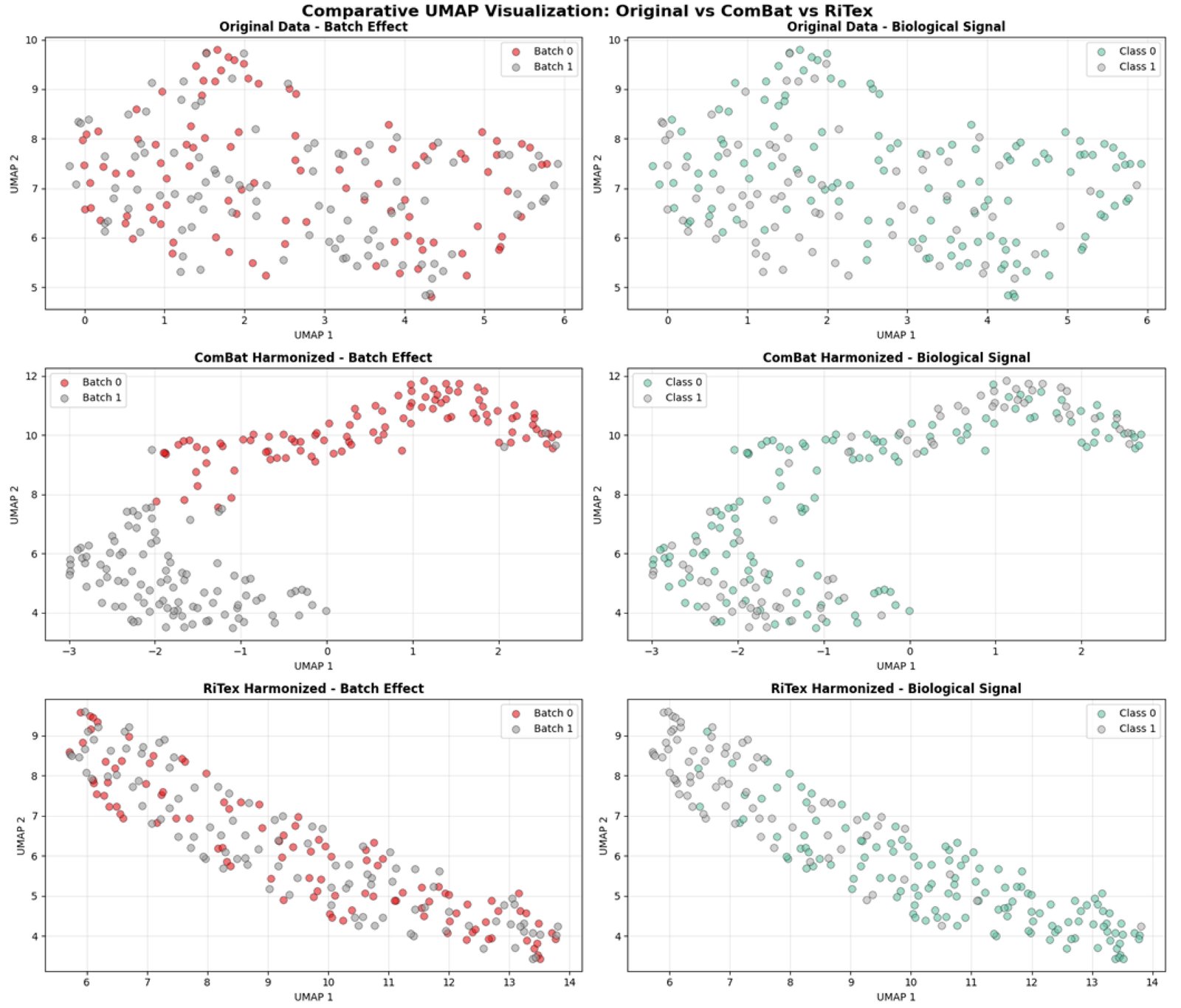
Dataset «WORC-GIST»	
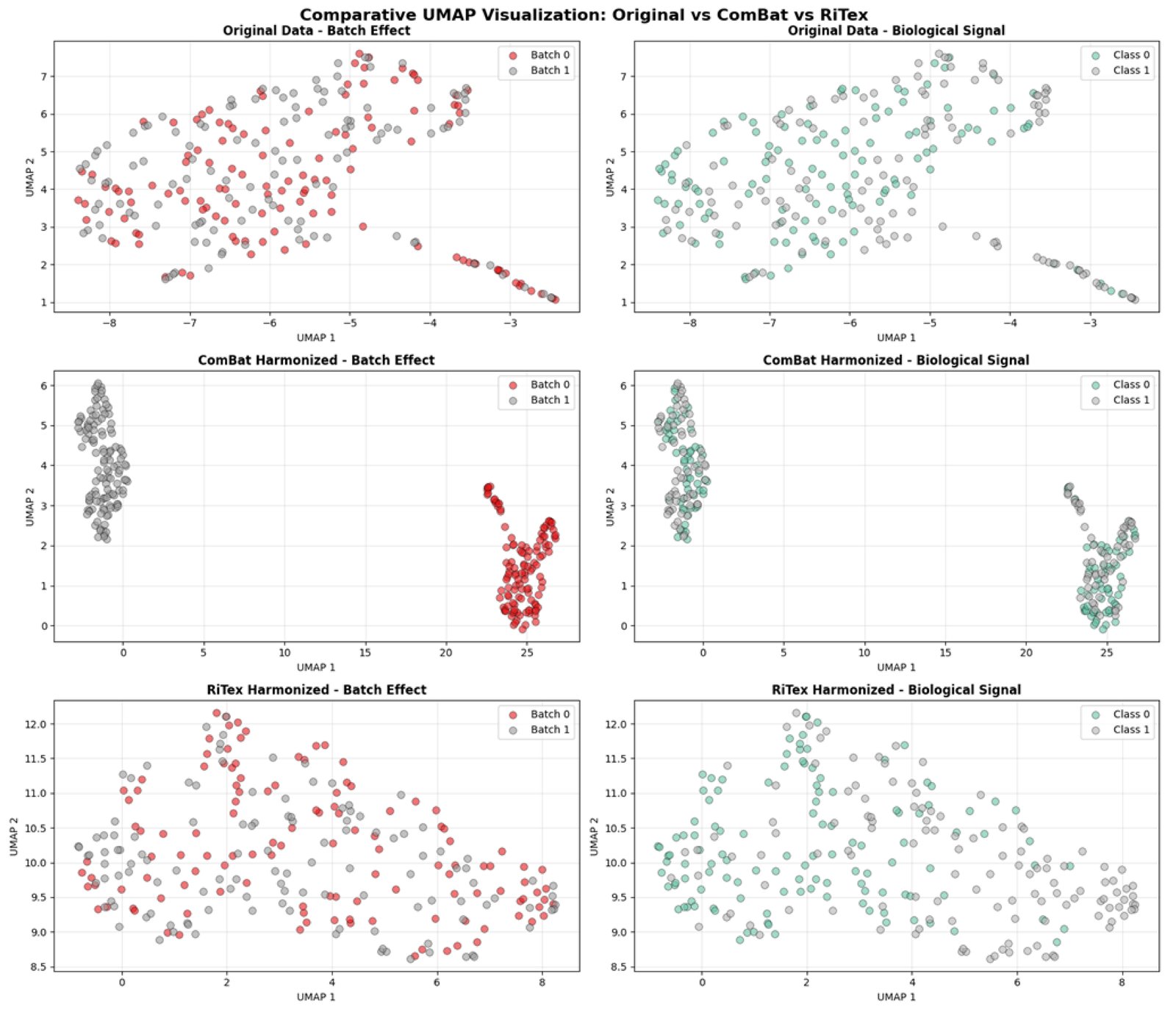
Dataset «WORC-Lipo»	
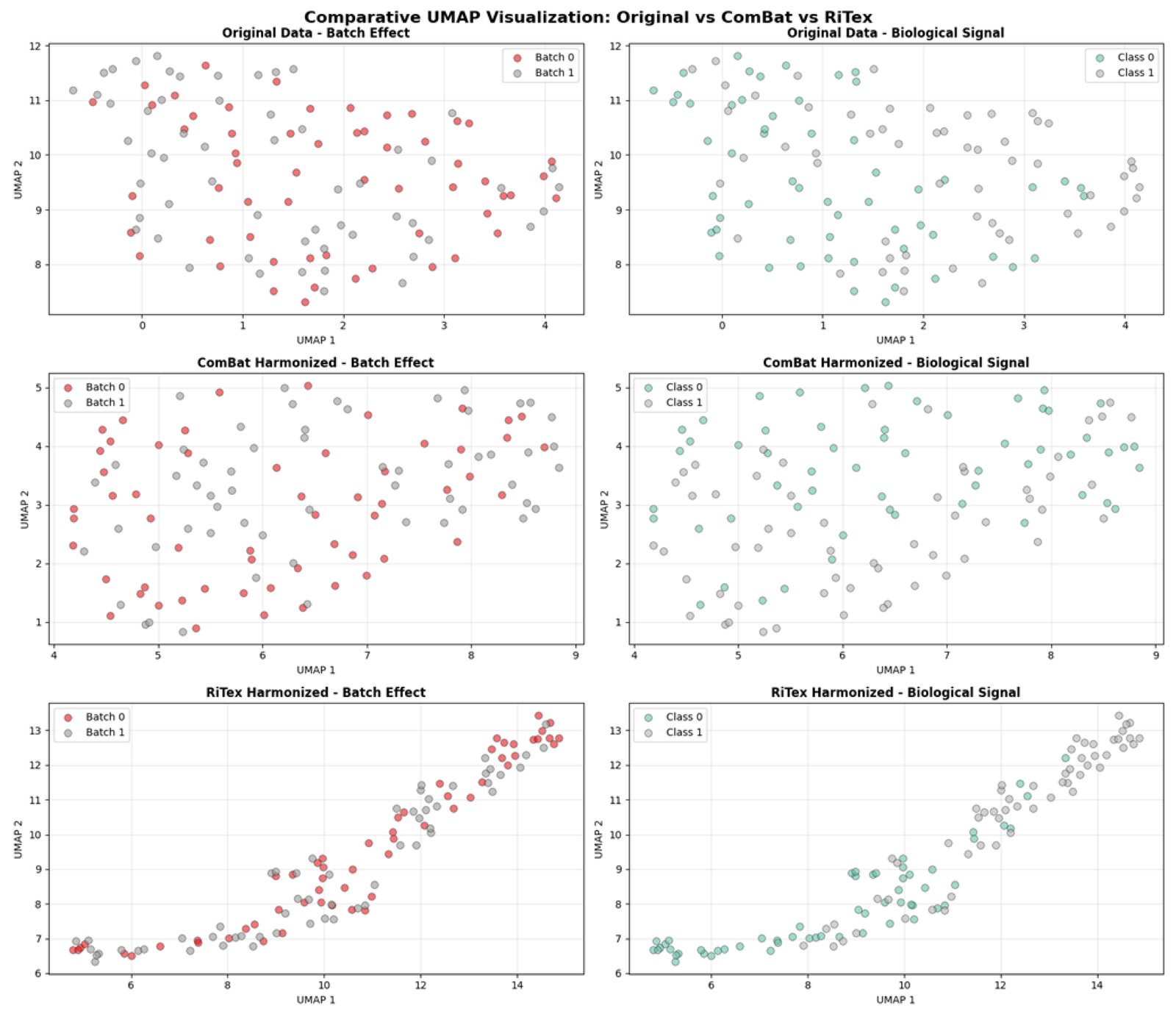
Dataset «WORC-Liver»	
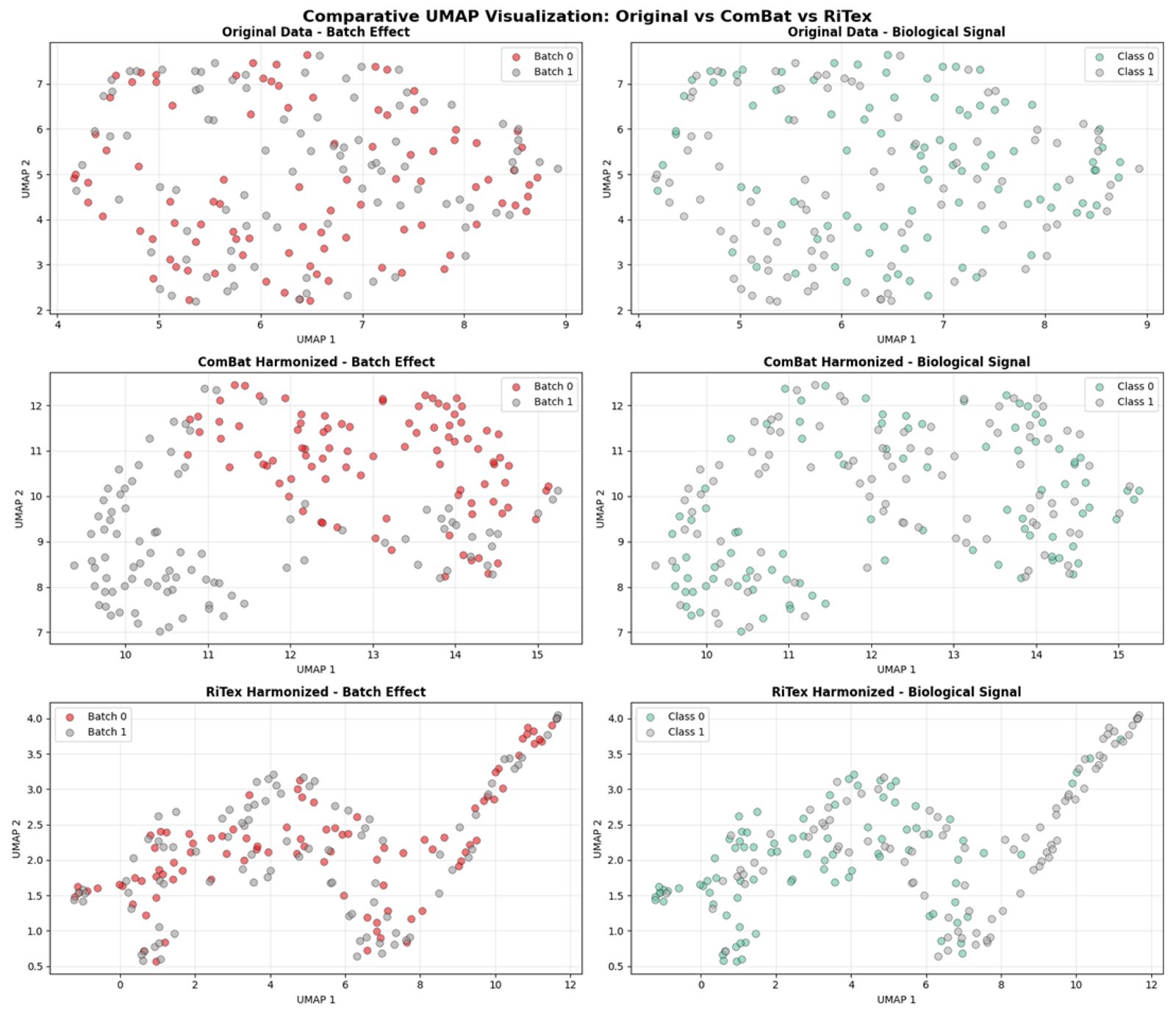
Dataset «WORC-Melanoma»	
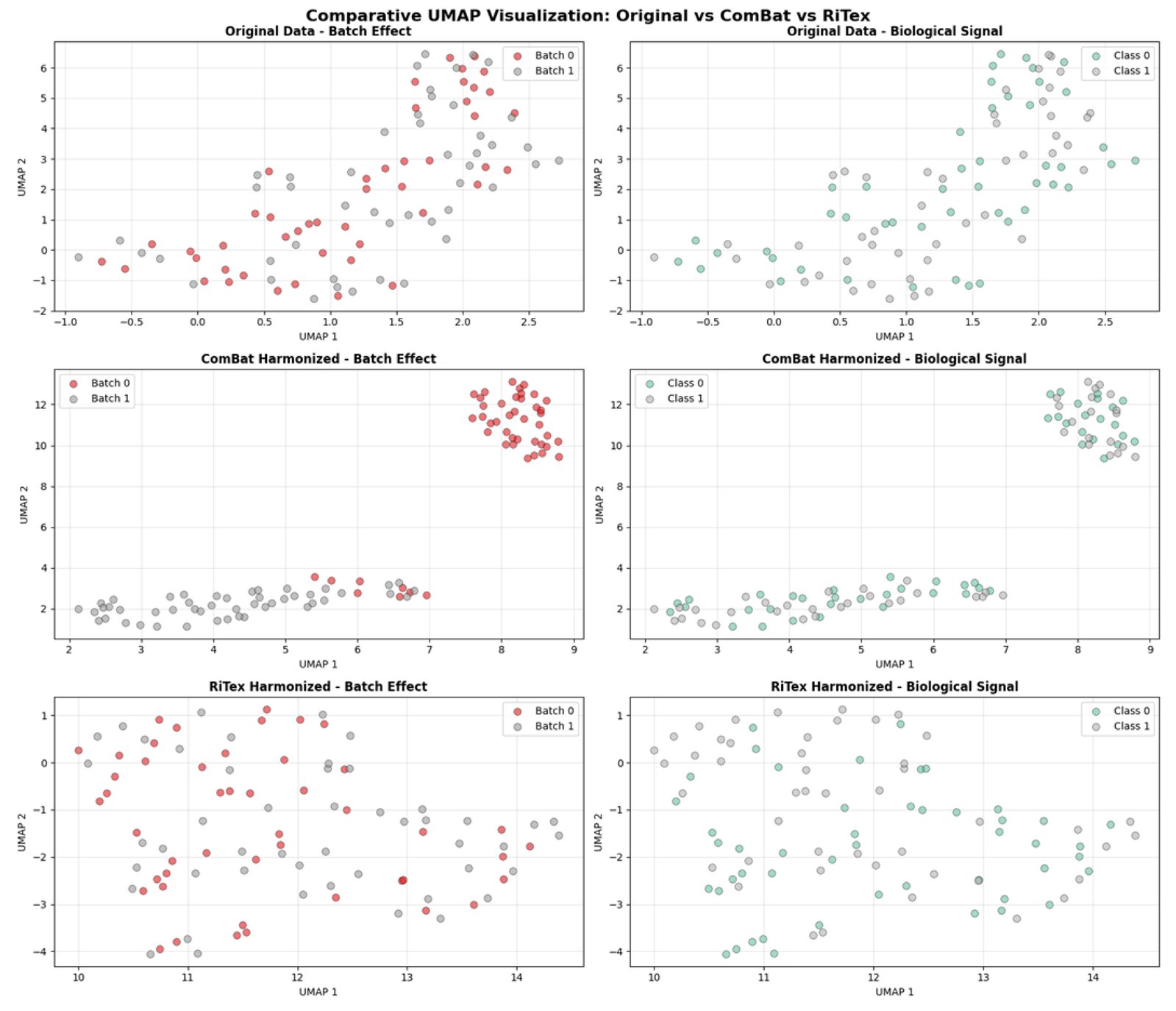
Dataset «Wang2024»	
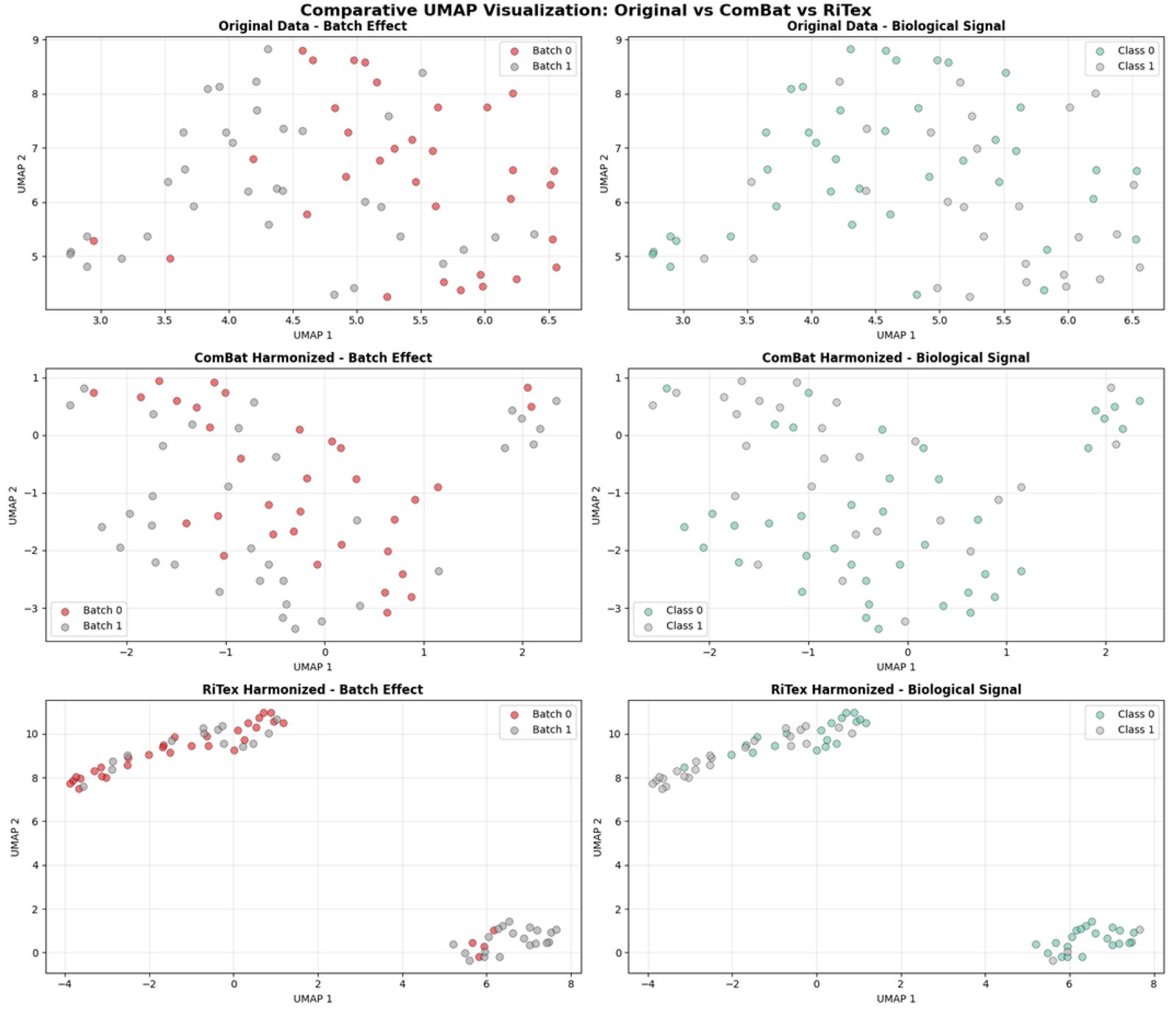
Dataset «Zhang2023»	
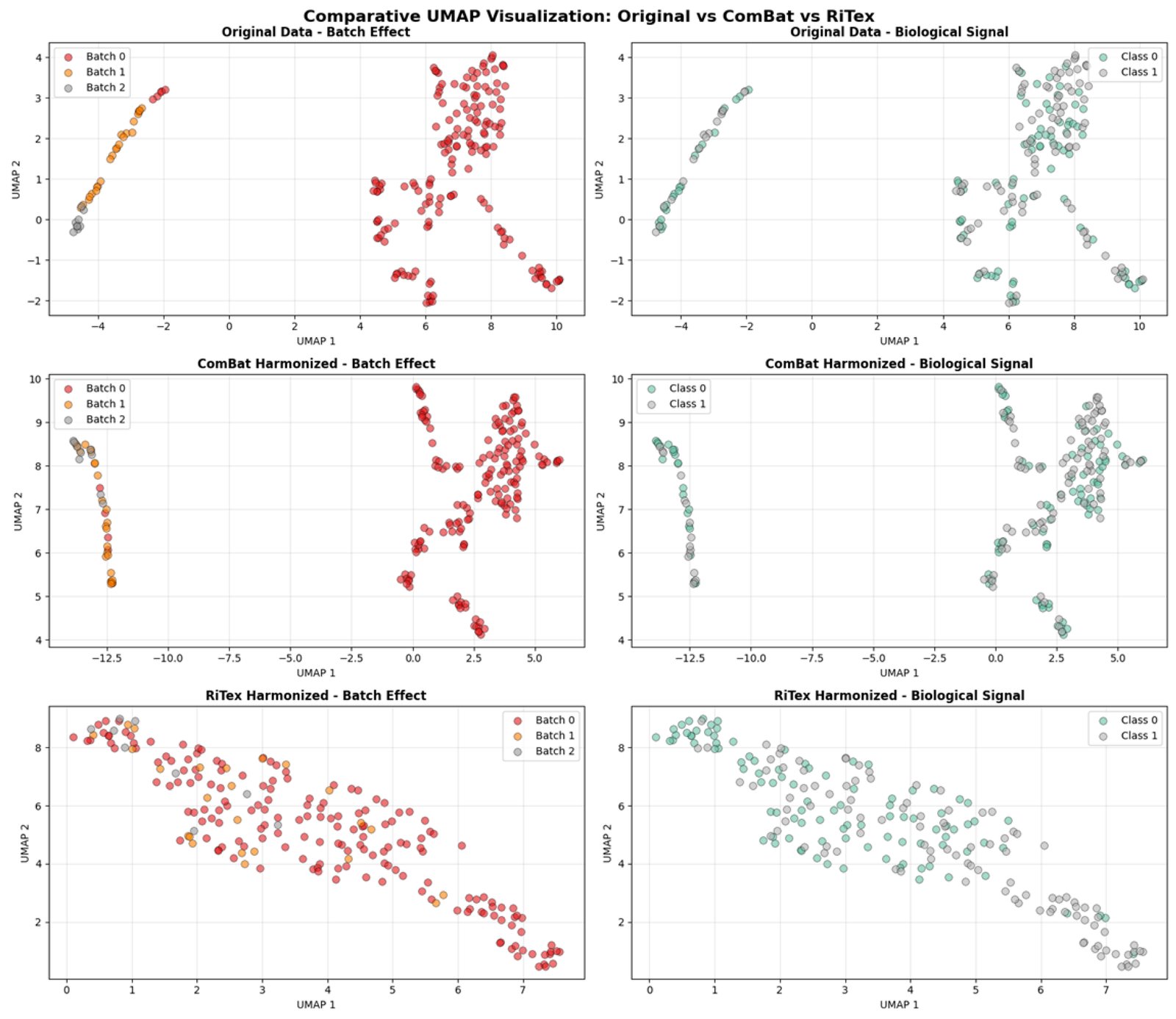
Dataset «Zhang2024A»	
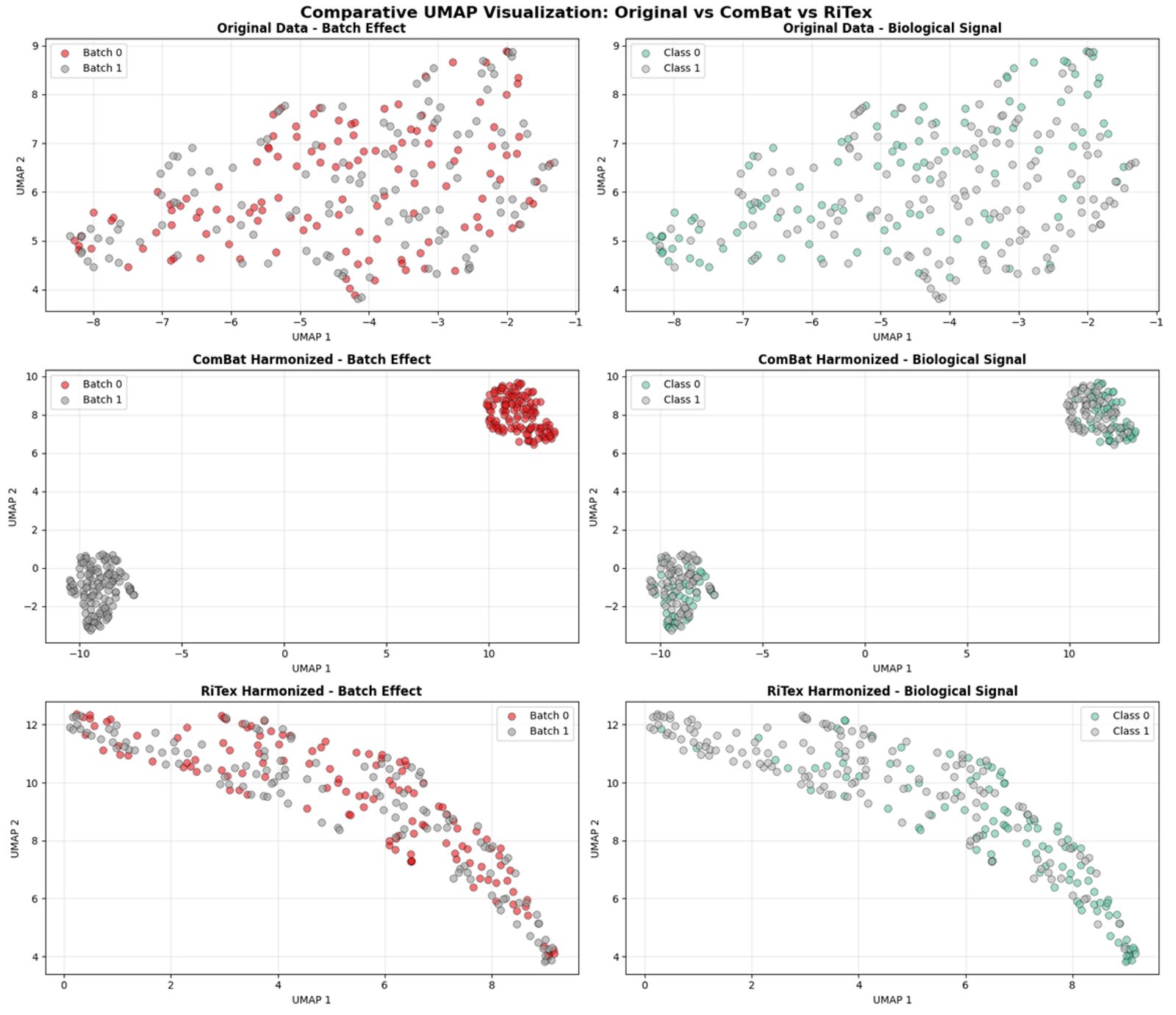
Dataset «Zhang2024B»	
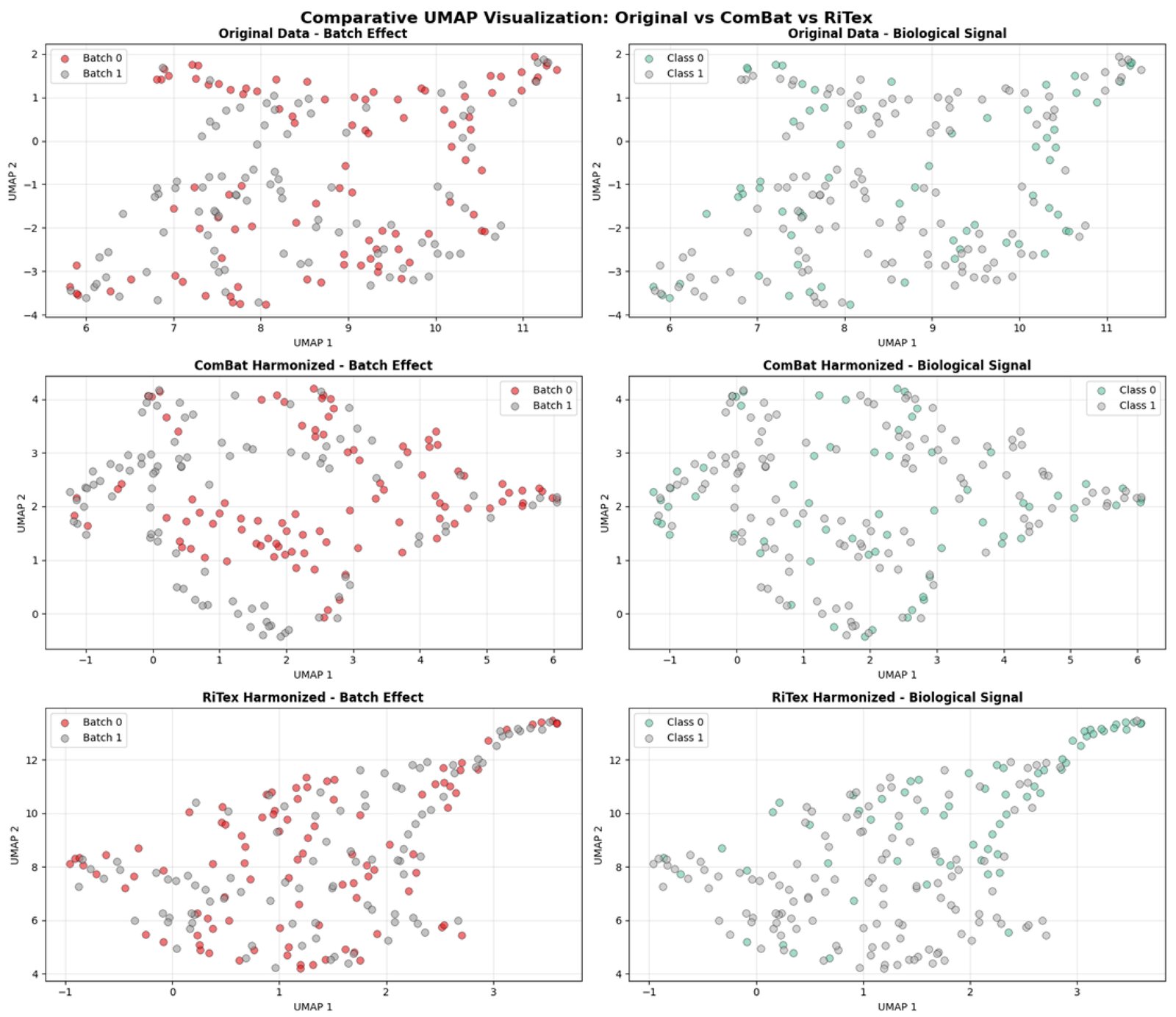

## Appendix C

### Appendix C.1

Based on optimal transport, the Wasserstein distance between distributions μ and is ν  Formula (A28) is determined:

(A28)
$$W_{p}\left(\mu ,\nu \right)={\left(\underset{\pi \in \Pi \left(\mu ,\nu \right)}{inf}\;\int \;\int \;\|x-y{\|}_{p}^{p}d\pi (x,y)\right)}^{\frac{1}{p}}\;$$

The Wasserstein-2 distance ( p=2 ), often referred to as the Fréchet distance, is widely used in machine learning applications.

### Appendix C.2

#### Appendix C.2.1

On a Riemannian manifold (M,g) with a metric g , the Wasserstein distance is defined as Formula (A29):

(A29)
$$W_{2}^{\mathrm{Riem}}\left(\mu ,\nu \right)={\left(\underset{\pi \in \Pi \left(\mu ,\nu \right)}{inf}\;\int \;\int \;d_{g}(x,y{)}^{2}d\pi (x,y)\right)}^{\frac{1}{2}}$$

where $d_{g}(x,y)$ is the Riemannian distance between points x and y on the manifold M .

#### Appendix C.2.2

For the space of SPD matrices $S_{++}^{n}$ with the affine-invariant geometry metric, the Riemannian Wasserstein distance can be computed by solving the optimal transport problem in the eigenvalue space or by using an approximation based on logarithmic transformations.

Specifically, for two distributions μ and ν on the space of SPD matrices, if they have finite second moments, the Wasserstein distance can be calculated as Formula (A30):

(A30)
$$W_{2}^{\mathrm{Riem}}(\mu ,\nu {)}^{2}=\mathrm{tr}(\Sigma_{1}+\Sigma_{2}-2\left(\Sigma_{2}^{\frac{1}{2}}\Sigma_{1}\Sigma_{2}^{\frac{1}{2}}{)}^{\frac{1}{2}}\right)$$

where $\Sigma_{1}$ and $\Sigma_{2}$ are the means (Fréchet centroids) of the distributions μ and ν in the Riemannian metric on $S_{++}^{n}$ .

### Appendix C.3

In the context of batch effect harmonization, the RiTex problem can be reformulated as an optimal transport problem. Suppose that for each batch g , the features form a distribution $\mu_{g}$ within the feature space. Harmonization seeks a transport map $\pi_{g}$ that transforms a distribution $\mu_{g}$ into a target distribution $\mu_{0}$ (i.e., the global distribution or the reference batch distribution), such that:

1. Transport cost is minimized (the proximity of the harmonized features to the target distribution);
2. Biological information preservation is maximized (the conservation of transformation).

RiTex implicitly addresses this by applying geodesic transformations on the Riemannian manifold of SPD matrices that minimize the Wasserstein distance between the batch-specific and target distributions while preserving the local geometry of the features.

### Appendix C.4

When estimating batch-specific parameters, RiTex implicitly solves an optimal transport problem on a Riemannian manifold. Calculating the Fréchet centroid (Fréchet mean) ${\bar{X}}_{g}$ for each batch using Formula (A31):

(A31)
$${\bar{X}}_{g}=arg\underset{X\in S_{++}^{n}}{min}\;\sum_{i\in g}\;d_{g}(X,X_{i}{)}^{2}\;$$

corresponds to finding the midpoint in the sense of optimal transport on a Riemannian manifold. Applying a geodesic transformation for batch effect correction is equivalent to identifying the geodesic path in the space of optimal transport between the batch-specific and global distributions.

## Appendix D

### Appendix D.1

**Theorem A3** (Geodesics on an SPD manifold)**.** *Let* $S_{++}^{n}$ *be the space of symmetric positive-definite* n×n *matrices, equipped with an affine-invariant Riemannian metric. A geodesic* γ(t) *between two points* $X,Y\in S_{++}^{n}$ *can be represented with Formula (A32*)*:*

(A32)
$$\gamma (t)=X^{\frac{1}{2}}(X^{-\frac{1}{2}}YX^{-\frac{1}{2}}{)}^{t}X^{\frac{1}{2}},t\in \left[0,1\right]$$

*where* $(\cdot {)}^{t}$ *denotes the matrix degree. The Riemannian distance between* X *and* Y *is defined as Formula (A33)*:

(A33)
$$d(X,Y)=\|log(X^{-\frac{1}{2}}YX^{-\frac{1}{2}}){\|}_{F\;}$$

*where* log *is the matrix logarithm, and* $\|\cdot {\|}_{F}$ *is the Frobenius norm.*
**Proof.** An affine-invariant metric within space $S_{++}^{n}$ is defined in the tangent space $T_{X}S_{++}^{n}$ as Formula (A34):

(A34)
$$g_{X}\left(u,v\right)=\mathrm{tr}\left(X^{-1}uX^{-1}v\right)$$

for vectors $u,v\in T_{X}S_{++}^{n}$ . Geodesics on a Riemannian manifold are defined by Equation (A35):

(A35)
$$\frac{D}{dt}\dot{\gamma }\left(t\right)=0$$

where D/dt is the covariant derivative along the curve γ(t) . For a space $S_{++}^{n}$ with an affine-invariant metric, the Christoffel symbols possess a structure that yields a geodesic solution in the form of a matrix exponential. The initial conditions for the geodesic are γ(0)=X , $\dot{\gamma }(0)=V$ , where V is the initial tangent vector chosen, such that the geodesic passes through Y at t=1 . The solution of the geodesic equations is given by Formula (A36):

(A36)
$$\gamma \left(t\right)=X^{\frac{1}{2}}\exp\left(t\log\left(X^{-\frac{1}{2}}YX^{-\frac{1}{2}}\right)\right)X^{\frac{1}{2}}$$

which may be simplified using the matrix power, as shown in Formula (A37):

(A37)
$$\gamma (t)=X^{\frac{1}{2}}(X^{-\frac{1}{2}}YX^{-\frac{1}{2}}{)}^{t}X^{\frac{1}{2}}\;$$

The length of this curve, according to the definition of the geodesic distance, is given by Formula (A38):

(A38)
$$d(X,Y)=\int_{0}^{1}\;\sqrt{g_{\gamma \left(t\right)}\left(\dot{\gamma }\left(t\right),\dot{\gamma }\left(t\right)\right)}dt=\|\log\left(X^{-\frac{1}{2}}YX^{-\frac{1}{2}}\right){\|}_{F}$$

which completes the proof. □
**Theorem A4** (variational characterization of the eigenvalue problem)**.** *Let* $A\in S_{++}^{n}$ *. The eigenvalues of the matrix* A *can be characterized using the following variational problem. The maximum eigenvalue* $\lambda_{max}(A)$ *is given by Formula (A39)*:

(A39)
$$\lambda_{max}\left(A\right)=\underset{\left\|v\right\|=1}{max}\;v^{T}Av=\underset{\left\|v\right\|=1}{max}\;\left\langle Av,v\right\rangle \;$$

*The minimum eigenvalue* $\lambda_{min}(A)$ *is given by Formula (A40)*:

(A40)
$$\lambda_{min}\left(A\right)=\underset{\left\|v\right\|=1}{min}\;v^{T}Av$$

*V denotes the* n×(k−1) *matrix, whose orthogonal span is a (k − 1)-dimensional subspace of* $R^{n}$ *. The trace ratio is invariant under any orthogonal re-parametrisation of those columns. A more general statement (Rayleigh’s min-max theorem*) [47]*: The* k *-th largest eigenvalue* $\lambda_{k}(A)$ *(in descending order) can be represented as Formula (A41)*:

(A41)
$$\lambda_{k}\left(A\right)=\underset{V\in R^{n\times \left(k-1\right)}}{min}\;\underset{\left\|v\right\|=1,v⊥V}{max}\;v^{T}Av$$

**Proof.** Consider the spectral decomposition of the matrix A , Formula (A42):

(A42)
$$A=\sum_{i=1}^{n}\;\lambda_{i}e_{i}e_{i}^{T}\;$$

where $\lambda_{1}\geq \lambda_{2}\geq \cdots \geq \lambda_{n}$ are the eigenvalues arranged in descending order, and $\left\{e_{1},\ldots ,e_{n}\right\}$ is an orthonormal set of eigenvectors. For an arbitrary vector v with ‖v‖=1 , we can write Formula (A43):

(A43)
$$v=\sum_{i=1}^{n}\;c_{i}e_{i},\sum_{i=1}^{n}\;c_{i}^{2}=1$$

Then, using Formula (A44):

(A44)
$$v^{T}Av=\sum_{i=1}^{n}\;\lambda_{i}c_{i}^{2}\leq \lambda_{1}\sum_{i=1}^{n}\;c_{i}^{2}=\lambda_{1}\;$$

Equality is achieved for $v=e_{1}$ , which proves the first part of Formula (A45):

(A45)
$$\lambda_{max}\left(A\right)=\underset{\left\|v\right\|=1}{max}\;v^{T}Av=\lambda_{1}$$

To prove the min-max characterization of $\lambda_{k}$ , assume that we are given an (k−1) -dimensional subspace V generated by the vectors $v_{1},\ldots ,v_{k-1}$ . For a vector v such that v⊥V and ‖v‖=1 , Formula (A46):

(A46)
$$v^{T}Av\leq \lambda_{1}\sum_{i=1}^{n}\;c_{i}^{2}=\lambda_{1}$$

But this upper bound does not take into account the orthogonality of v to V . Key observation: The set of all vectors orthogonal to an arbitrary (k−1) -dimensional subspace contains the (n−k+1) -dimensional subspace. In particular, if we choose $V=\mathrm{span}\{e_{1},\ldots ,e_{k-1}\}$ , then all vectors $v=\sum_{i=k}^{n}\;c_{i}e_{i}$ are orthogonal to V , and by Formula (A47):

(A47)
$$\underset{\|v\|=1,v⊥V}{max}\;v^{T}Av=\underset{\left\|v\|=1,v\in \mathrm{span}\{e_{k},\ldots ,e_{n}\}\right\}}{max}\;v^{T}Av=\lambda_{k}\;$$

On the other hand, for any other choice of subspace V′ of dimension k−1 , the complementary space $\left(R^{n}/V\prime \right)$ contains a direction whose coefficient with respect to an eigenvector $e_{k}$ (or earlier eigenvectors) is positive. Then, using Formula (A48):

(A48)
$$\underset{V\in R^{n\times (k-1)}}{min}\;\underset{\|v\|=1,v⊥V}{max}\;v^{T}Av=\lambda_{k}$$

which completes the proof. □
**Theorem A5** (Ledoit–Wolf optimality)**.** *Let* $\overset{ˆ}{\Sigma }$ *be the sample covariance matrix from a sample of size* N *, and let* F *be some target matrix (usually a scalar matrix* μI *or other structured matrix). The Ledoit–Wolf estimator defined by Formula (A49)*:

(A49)
$${\overset{ˆ}{\Sigma }}_{\mathrm{LW}}=\left(1-\alpha^{*}\right)\overset{ˆ}{\Sigma }+\alpha^{*}F\;$$

*where the shrinkage coefficient* $\alpha^{*}$ *is chosen optimally and minimizes the expected squared error using Formula (A50)*:

(A50)
$$\alpha^{*}=arg\underset{\alpha \in \left[0,1\right]}{min}\;E[\left\|{\overset{ˆ}{\Sigma }}_{\mathrm{LW}}-\Sigma {\|}_{F}^{2}\right]$$

*under certain regularity conditions on the data distribution.*
**Proof.** Consider a Bayesian estimation problem. Suppose a sample $x_{1},\ldots ,x_{N}$ is obtained from a normal distribution N(0,Σ) with sample size N . The sample covariance matrix, using Formula (A51):

(A51)
$$\overset{ˆ}{\Sigma }=\frac{1}{N}\sum_{i=1}^{N}\;x_{i}x_{i}^{T}$$

is an unbiased estimator of Σ , but may be ill-conditioned (especially when p>N or p is close to N ). The class of estimators of the form ${\overset{ˆ}{\Sigma }}_{\mathrm{shrink}}(\alpha )=(1-\alpha )\overset{ˆ}{\Sigma }+\alpha F$ forms a convex combination of the empirical covariance and the target matrix. The squared error of the estimate, as defined by Formula (A52):

(A52)
$$E(\alpha )=E[\left\|\left(1-\alpha \right)\overset{ˆ}{\Sigma }+\alpha F-\Sigma {\|}_{F}^{2}\right]\;$$

is decomposed by Formula (A53):

(A53)
$$E(\alpha )=(1-\alpha {)}^{2}E[\|\overset{ˆ}{\Sigma }-\Sigma {\|}_{F}^{2}]+2\alpha (1-\alpha )E[\langle \overset{ˆ}{\Sigma }-\Sigma ,F-\Sigma \rangle ]+\alpha^{2}\|F-\Sigma {\|}_{F}^{2}$$

Minimizing with respect to α according to formula (A54):

(A54)
$$\frac{dE(\alpha )}{d\alpha }=-2(1-\alpha )E[\|\overset{ˆ}{\Sigma }-\Sigma {\|}_{F}^{2}]+2E[\langle \overset{ˆ}{\Sigma }-\Sigma ,F-\Sigma \rangle ]+2\alpha \|F-\Sigma {\|}_{F}^{2}=0$$

Then, using Formula (A55):

(A55)
$$\alpha^{*}=\frac{E[\|\overset{ˆ}{\Sigma }-\Sigma {\|}_{F}^{2}]-E[\langle \overset{ˆ}{\Sigma }-\Sigma ,F-\Sigma \rangle ]}{E[\|\overset{ˆ}{\Sigma }-\Sigma {\|}_{F}^{2}]+\|F-\Sigma {\|}_{F}^{2}}$$

In practice, the moment estimators of Ledoit & Wolf replace the known Σ:

$$\hat{\mu }=\frac{tr\left(\hat{\Sigma }\right)}{p}$$

$${\hat{d}}^{2}={\left\|\hat{\Sigma }-\hat{\mu }I\right\|}_{F}^{2}$$

$${\hat{b}}^{2}=min({\hat{d}}^{2},\frac{1}{N^{2}}\sum_{k=1}^{N}{\left\|x_{k}x_{k}^{T}-\hat{\Sigma }\right\|}_{F}^{2})$$

$${\hat{\alpha }}^{*}=min(1,\frac{{\hat{b}}^{2}}{{\hat{d}}^{2}})$$

This estimator is implemented in sklearn.covariance.LedoitWolf, which is used throughout this work. Ledoit and Wolf showed that this optimal $\alpha^{*}$ can be consistently estimated from the data without prior knowledge of the true covariance Σ . Specifically, it was proven that, for N→∞ , the coefficient estimate converges to the true optimal value with probability 1. Furthermore, the Ledoit–Wolf estimator guarantees positive definiteness ${\overset{ˆ}{\Sigma }}_{\mathrm{LW}}$ , provided that both $\overset{ˆ}{\Sigma }$ and F are positive definite, which is critical for harmonizing the batch effects in the context of SPD matrices. □

### Appendix D.2

**Theorem A6.** (properties of metric $S_{\mathrm{bio}}$ )**.** *Let* $T={U\Sigma }^{-\frac{1}{2}}$ *be a whitening transform, followed by an orthogonal rotation U* ∈O(p) *, and let* h(z)= σ(w∗z  *+ b* ) *be an affine probe classifier. Then* AUC(h∘T)=AUC(h). *We define a metric for the preservation of biological information using Formula (A56):*

(A56)
$$S_{\mathrm{bio}}=\frac{1}{n}\sum_{i=1}^{n}\frac{{\mathrm{AUC}}_{i}^{\mathrm{bio}}\left(\mathrm{harmonized}\right)}{{\mathrm{AUC}}_{i}^{\mathrm{bio}}\left(\mathrm{original}\right)}\;$$

*The following properties are satisfied under certain regularity conditions:*

1. ***Value bounds:*** $S_{\mathrm{bio}}$ ∈ (0, ∞) in principle. Empirically, $S_{\mathrm{bio}}$ ∈ [0.83, 1.07] across the 50 radMLBench datasets, with $S_{\mathrm{bio}}$ ≥ 1 occurring on 27/50. Theorem A6 establishes the asymptotic monotonicity $S_{\mathrm{bio}}$ → 1^−^ under perfect harmonization but does not require $S_{\mathrm{bio}}$ ≤ 1 in finite samples.
2. ***Consistency:*** If the overall transformation consists of a composite of two harmonizing transformations, then the $S_{\mathrm{bio}}$ of the composite is no worse than the geometric mean $S_{\mathrm{bio}}$ of individual transformations.

**Proof.** 

1. **Value bounds:** Each ${\mathrm{AUC}}_{i}^{\mathrm{bio}}$ is, by definition, within the interval [0,1] . The relationship ${\mathrm{AUC}}_{i}^{\mathrm{bio}}(\mathrm{harmonized})/{\mathrm{AUC}}_{i}^{\mathrm{bio}}(\mathrm{original})$ can only be >1 if harmonization has improved the predictive power of the feature, which is rare in practice. Thus, $S_{\mathrm{bio}}\leq 1$ . On the other hand, $S_{\mathrm{bio}}>0$ , provided that the original features have at least some predictive power;
2. **Monotonicity:** Consider two models:
  ○ Model 1: $y∼f(x^{\mathrm{original}})$
  ○ Model 2: $y∼f(x^{\mathrm{harmonized}})$ ;
3. If harmonization applies a linear transformation $x^{\mathrm{harmonized}}=T(x^{\mathrm{original}})$ , where T commutes with the batch structure (i.e., is applied uniformly to all samples regardless of the batch), then the logistic regression in the new coordinates has an equivalent predictive power before scaling. More formally, if T is a linear orthogonal transformation, then AUC is invariant under T , which implies $S_{\mathrm{bio}}=1$ ;
4. **Consistency:** Let $T_{1}$ and $T_{2}$ be two successive harmonization transformations. Consider the composition $T=T_{2}\circ T_{1}$ . Then, using Formula (A57):
  (A57)
  $$S_{\mathrm{bio}}\left(T\right)=\frac{1}{n}\sum_{i}\;\frac{{\mathrm{AUC}}_{i}\left(T_{2}\left(T_{1}\left(x\right)\right)\right)}{{\mathrm{AUC}}_{i}\left(x\right)}$$

By the mean theorem, by Formula (A58):

(A58)
$$\frac{{\mathrm{AUC}}_{i}(T_{2}(T_{1}(x)))}{{\mathrm{AUC}}_{i}(x)}=\frac{{\mathrm{AUC}}_{i}(T_{2}(T_{1}(x)))}{{\mathrm{AUC}}_{i}(T_{1}(x))}\cdot \frac{{\mathrm{AUC}}_{i}\left(T_{1}\left(x\right)\right)}{\;{\mathrm{AUC}}_{i}\left(x\right)}$$

Then, using Formula (A59):

(A59)
$$S_{\mathrm{bio}}(T)\geq (S_{\mathrm{bio}}\left(T_{1}\right)\cdot S_{\mathrm{bio}}\left(T_{2}\right){)}^{\frac{1}{2}}$$

on average over features. □
**Theorem A7** (handling data heterogeneities)**.** *If the data contains sub-batches with different batch effects within a single nominal batch, the RiTex method still converges to a consistent estimate of the global centroid, provided that the number of sub-batches grows sublinearly with the batch size.*
**Proof.** Consider a batch g containing $M_{g}$ sub-batches, each with a centroid ${\bar{X}}_{g,j}$ . Instead of a single centroid, hierarchical clustering identifies these sub-batches, and the algorithm applies an iterative procedure according to Formula (A60):

(A60)
$${\bar{X}}_{g}^{\left(k+1\right)}=\frac{1}{M_{g}}\sum_{j=1}^{M_{g}}\;\mathrm{Geometric}\;\mathrm{Mean}\left({\bar{X}}_{g,j}^{\left(k\right)},{\bar{X}}_{\mathrm{global}}\right)$$

The convergence of this procedure follows from the fact that the geodesic mean on the Riemannian manifold of SPD matrices is unique and is a fixed point of this iteration. The error from incorrect clustering, by Formula (A61):

(A61)
$$\left\|{\bar{X}}_{\mathrm{estimated}}-{\bar{X}}_{\mathrm{true}}\right\|\leq O\left(\frac{M_{g}}{\left|g\right|}\right)$$

where |g| is the batch size. Given $M_{g}=o(|g|)$ (that is, the number of sub-batches grows more slowly than the batch size), the error tends to zero as |g|→∞ . □

### Appendix D.3

**Lemma A1** (distribution of the F-statistic in the presence of batch effects)**.** *Let* $x_{gi}\in R^{p}$ *be features, where* g *is a batch, and* i *is a sample. A model with a batch effect is represented by Formula (A62*)*:*

(A62)
$$x_{gi}=\mu +\delta_{g}+\varepsilon_{gi}$$

*where* μ *is the global mean,* $\delta_{g}$ *is the batch effect, and* $\varepsilon_{gi}∼N(0,\sigma^{2})$ *is the noise. The F-statistic for testing the significance of a batch effect is given by Formula (A63)*:

(A63)
$$F=\frac{{\mathrm{SS}}_{\mathrm{batch}}/(m-1)}{{\mathrm{SS}}_{\mathrm{residual}}/(N-m)}$$

*where* m *is the number of batches and* N *is the total number of samples and follows the distribution* $F_{m-1,N-m}$ *under the null hypothesis (absence of a batch effect).*
**Proof.** In classical ANOVA, assuming normal errors and equal variances between batch variables, the sum of squares for the batch effect is given by Formula (A64):

(A64)
$${\mathrm{SS}}_{\mathrm{batch}}=\sum_{g}\;n_{g}({\bar{x}}_{g}-\bar{x}{)}^{2}\;$$

and the residual sum of squares is given by Formula (A65):

(A65)
$${\mathrm{SS}}_{\mathrm{residual}}=\sum_{g,i}\;(x_{gi}-{\bar{x}}_{g}{)}^{2}$$

have the following distributions under the null hypothesis:

- ${\mathrm{SS}}_{\mathrm{batch}}/\sigma^{2}∼\chi_{m-1}^{2}$ ;
- ${\mathrm{SS}}_{\mathrm{residual}}/\sigma^{2}∼\chi_{N-m}^{2}$ ;
- These two statistics are independent.

Therefore, the ratio given by Formula (A66):

(A66)
$$F=\frac{{\mathrm{SS}}_{\mathrm{batch}}/(m-1)}{{\mathrm{SS}}_{\mathrm{residual}}/(N-m)}=\frac{\chi_{m-1}^{2}/(m-1)}{\chi_{N-m}^{2}/(N-m)}\;$$

follows a Fisher distribution $F_{m-1,N-m}$ with degrees of freedom for the numerator m−1 and denominator N−m . □
**Lemma A2** (PSD property of a combined matrix)**.** *Let* $A,B\in S_{++}^{n}$ *be two symmetric positive-definite matrices, and* α,β∈[0,1] *, such that* α+β=1 *. The combined matrix given by Formula (A67)*:

(A67)
C=αA+βB

*is also positive-definite. Furthermore, inequality (A68) holds for the eigenvalues:*

(A68)
$$\lambda_{min}\left(C\right)\geq \alpha \lambda_{min}\left(A\right)+\beta \lambda_{min}\left(B\right)$$

**Proof.** Positive definiteness: For an arbitrary vector v≠0 according to formula (A69):

(A69)
$$v^{T}Cv=\alpha v^{T}Av+\beta v^{T}Bv>0$$

since $v^{T}Av>0$ and $v^{T}Bv>0$ by assumption (as A and B are positive definite) and both coefficients α,β≥0 with α+β=1>0 . Inequality for the minimum eigenvalue: By the variational characterization theorem, Formula (A70) is:

(A70)
$$\lambda_{min}\left(C\right)=\underset{\left\|v\right\|=1}{min}\;v^{T}Cv=\underset{\left\|v\right\|=1}{min}\;\left(\alpha v^{T}Av+\beta v^{T}Bv\right)$$

The minimum is achieved at a certain $v^{*}$ , and by Formula (A75):

(A71)
$$\lambda_{min}\left(C\right)=\alpha v^{*T}Av^{*}+\beta v^{*T}Bv^{*}\;$$

By the definition of the minimum eigenvalue, Formulas (A72) and (A73) are:

(A72)
$$v^{*T}Av^{*}\geq \lambda_{min}(A)\|v^{*}{\|}^{2}=\lambda_{min}\left(A\right)$$

(A73)
$$v^{*T}Bv^{*}\geq \lambda_{min}(B)\|v^{*}{\|}^{2}=\lambda_{min}\left(B\right)$$

Then, using Formula (A74):

(A74)
$$\lambda_{min}\left(C\right)\geq \alpha \lambda_{min}\left(A\right)+\beta \lambda_{min}\left(B\right)$$

Which completes the proof. □

### Appendix D.4

**Correlation between Bio AUC and Batch AUC**

The analysis of the experimental results shows that there is a negative correlation between Bio AUC and Batch AUC, reflecting a tradeoff between removing the batch effect and preserving biological information. Formally:

$$\rho_{\mathrm{Bio},\mathrm{Batch}}<-0.3$$

in most cases on the tested datasets.

**Complexity of Computing the Fréchet Centroid**

Calculating the Fréchet centroid (mean) of a set of SPD matrices $\left\{X_{1},\ldots ,X_{n}\right\}$ requires Formula (A75):

(A75)
$$\bar{X}=arg\underset{X\in S_{++}^{n}}{min}\;\sum_{i=1}^{n}\;d(X,X_{i}{)}^{2}$$

This problem does not have a closed-loop solution, but can be solved iteratively (for example, using the majorization minimization algorithm for the Karcher mean) using Formula (A76):

(A76)
$$X^{\left(k+1\right)}=X^{(k)1/2}{\left(\frac{1}{n}\sum_{i=1}^{n}\;\;(X^{(k)-1/2}X_{i}X^{(k)-1/2}{)}^{1/2}\right)}^{2}X^{(k)1/2}$$

Algorithm complexity: $O(n^{3}\cdot I)$ , where I is the number of iterations (usually I≈10–20 for convergence).

### Appendix D.5

Synthetic ground-truth experiment for silhouette-based k selection.

Motivation. In the radMLBench main experiment (Section 3.1 of the main text), true-center labels are unavailable, and the effective number of batches k is estimated automatically by silhouette-based KMeans. Table 3 of the main text shows that, on the H&N benchmark, this yields k ≈ 2 instead of the true k = 4, which collapses the Batch AUC suppression of RiTex from ΔBatch = −0.154 (real k) to ΔBatch = +0.191 (auto k)—a swing of 34.5 percentage points. The boundary condition stated in Section 4.2 (“Practitioners applying RiTex on data from which center metadata has been stripped should therefore expect behaviour closer to ComBat than to the multi-center result reported in Section 3.3”) rests on this single empirical observation. This section provides the systematic synthetic test that the boundary condition asks for.

Design: For each combination ( $k_{true}$ , ${sep}_{ratio}$ ), we generated 40 independent realizations of a Gaussian-mixture point cloud in ℝ^p with p = 105: $k_{true}$ ∈ {2, 3, 4, 5, 8}; n = 500 points per cluster; total N = 500 · $k_{true}$ points per realization.

Cluster centers are placed at the vertices of a regular ( $k_{true}$ − 1)-simplex embedded in the first $k_{true}$ coordinates, then padded with zeros to dimension p. The simplex edge length is ${sep}_{ratio}\times \sigma$ , where σ = 1 is the intra-cluster standard deviation. In the high-dimensional regime $\left\|x-\mu_{c}\right\|\approx \sqrt{p}\times \sigma$ ≈ 10.25, so a ${sep}_{ratio}$ = 4 corresponds to the centers that are about 0.4 of the intra-cluster radius, heavily overlapping, and ${sep}_{ratio}$ = 16 corresponds to clean separation. ${sep}_{ratio}$ ∈ {4, 8, 12, 16} has four difficulty regimes.

For each realization, we ran KMeans with $n_{init}$ = 3 for k ∈ {2, …, 10}, scoring each clustering by sklearn.metrics.silhouette_score on a uniform sub-sample of 600 points and selected k = argmax. Recovery accuracy was defined as the fraction of realizations with k = $k_{true}$ .

**Table A1:** Recovery accuracy of silhouette-based k selection (synthetic data, p = 105, n = 500 per cluster, 40 realizations per cell).

$k_{true}$	sep = 4 σ	sep = 8 σ	sep = 12 σ	sep = 16 σ	Mean Across sep
2	1.00	1.00	1.00	1.00	1.00
3	1.00	1.00	1.00	1.00	1.00
4	1.00	1.00	1.00	1.00	1.00
5	0.98	0.98	1.00	1.00	0.99
8	0.20	0.90	0.95	0.98	0.76

The mean k in each cell shows that the mode of failure is systematic under-counting. At $k_{true}$ = 8 with sep = 4 σ , the mean k is 6.92, i.e., silhouette merges the two least-separated pairs of clusters. The same systematic under-counting is what produces k ≈ 2 on the H&N benchmark (true k = 4).

In the clean-separation regime (sep ≥ 8 σ ) and for $k_{true}$ ≤ 5, silhouette-based selection recovers $k_{true}$ with an accuracy ≥ 0.98. This regime is the one in which the multi-center result of Section 3.3 (ΔBatch = −0.154) is recoverable from auto-detected pseudo-batches.

In the weak-separation regime (sep = 4 σ ), the accuracy drops to 0.20 already at k_true = 8, and the procedure systematically undercounts. The H&N benchmark, where the true centers are imaging-protocol cohorts whose radiomic signatures partially overlap, falls in this regime—which is why the auto-k result of Section 3.3 (ΔBatch = +0.191, with $\hat{\kappa }\;\approx$ 2) deviates so strongly from the real-label result.

This provides the quantitative basis for the conservative practitioner guidance in Section 4.2: when (a) the true center labels have been stripped from the data and (b) the radiomic signatures of the centers are not strongly separated, the auto-k variant of RiTex should be expected to behave closer to a 2-batch ComBat than to the multi-center RiTex result.
